# MARS: Machine-Learned Force Field Framework for Automated Conformational Sampling, Vibrational Spectroscopy, and Microsolvation

**DOI:** 10.1021/acs.jctc.6c01588

**Published:** 2026-10-21

**Authors:** Sergio Suárez-Dou, Miguel Gallegos, Florian N. Brünig, Alexandre Tkatchenko

**Affiliations:** Department of Physics and Materials Science, 81872 University of Luxembourg, L-1511 Luxembourg City, Luxembourg

## Abstract

We present MARS, an open-source Python package for automated conformational ensemble generation, infrared spectrum prediction, and explicit microsolvation of molecular systems. Built on JAX for GPU acceleration and automatic differentiation, MARS is carried out with machine-learned force fields (MLFFs), including SO3LR and MACE, with a four-phase conformational sampling workflow combining metadynamics, multitemperature molecular dynamics, and genetic algorithms. The package provides automated vibrational mode classification using Wilson B-matrix projections and functional group identification via graph-based pattern matching. We benchmark MARS on chemically diverse systems, including alkanes, halogenated aromatics, amino acids, peptides, drug-like molecules, phosphamides, host–guest complexes, and microsolvated biomolecular models. Semiempirical quantum-chemical methods have until now provided the practical state of the art for automated conformational sampling. Our benchmarks show that modern MLFFs can improve upon this paradigm by combining greater accuracy for high-level relative conformer energies with lower computational cost and more favorable scaling with molecular size. IR benchmarks against experimental gas-phase spectra additionally show performance comparable to quantum-chemical methods, with chemically interpretable mode assignments. Applications to alanine peptides, proline/β-cyclodextrin inclusion complexes, alanine dipeptide, and 2-methoxycyclohexanone further demonstrate that MARS captures conformational reorganization, noncovalent binding motifs, solvent-induced stability shifts, and spectroscopic signatures. These results establish MARS as a transferable and accessible platform for ensemble-based molecular modeling with machine-learned potentials. MARS is freely available at github.com/tcpunilu/mars.

## Graphical abstract

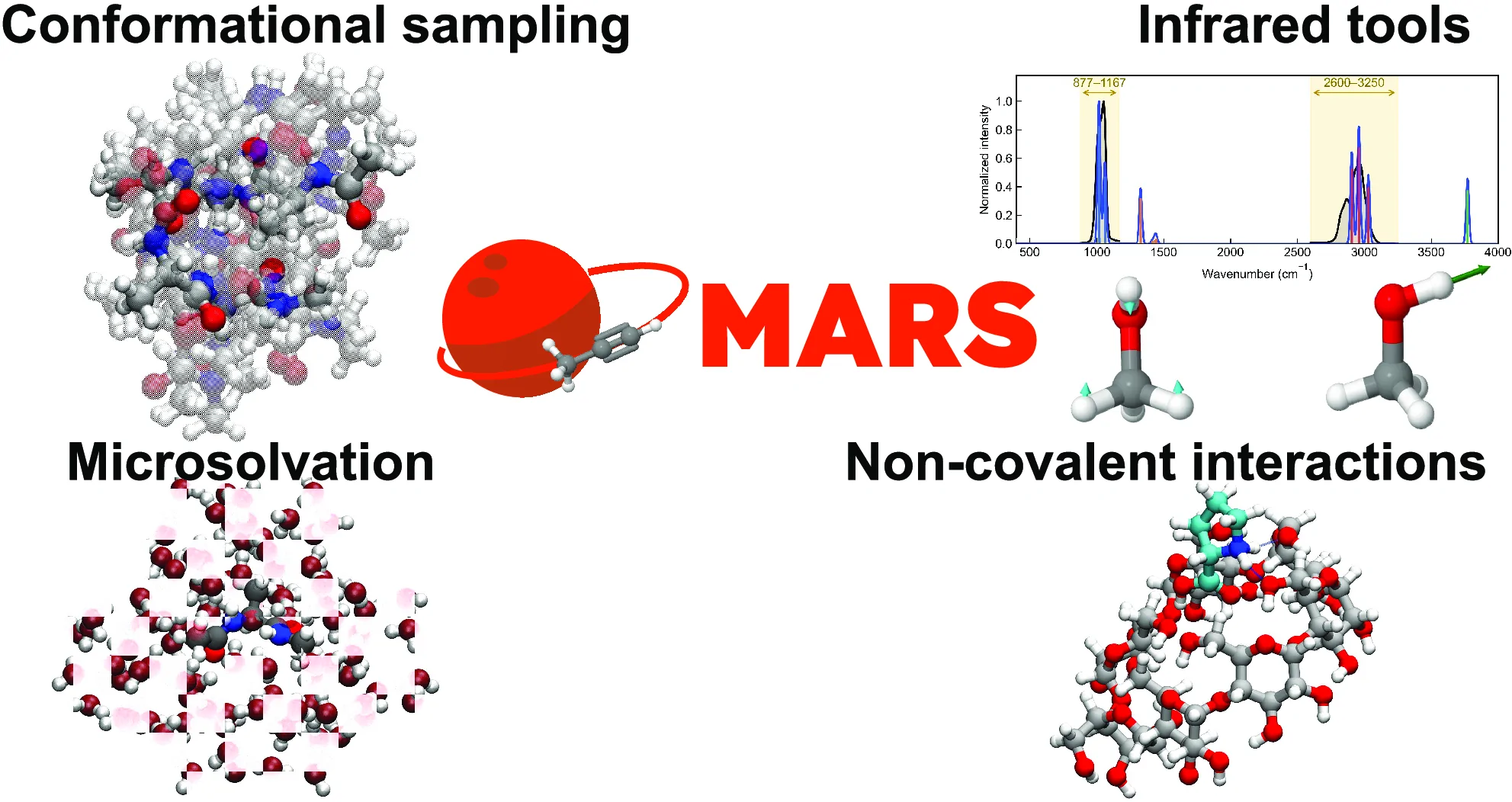

## 1 Introduction

The accurate prediction of molecular properties from first principles remains a central challenge in computational chemistry.^1,2^ For flexible molecules, thermodynamically meaningful observables—such as free energies, vibrational spectra, and reaction rates—require averaging over complex conformational ensembles. Exhaustive enumeration strategies based on systematic rotamer searches scale exponentially with the number of internal degrees of freedom, rendering them impractical for medium- to large-sized systems.^3^ Stochastic approaches, including molecular dynamics (MD) and Monte Carlo simulations, provide a more scalable alternative but often fail to efficiently traverse high free-energy barriers separating metastable conformational basins.^4–6^

Enhanced sampling methods, such as metadynamics, address this limitation by introducing history-dependent bias potentials that accelerate barrier crossing.^7^ These ideas have been successfully implemented in tools such as CREST,^8,9^ which combines metadynamics with the GFN-xTB semiempirical^10–12^ method to enable routine conformational exploration of drug-like molecules. This combination has established semiempirical quantum chemistry as the practical state of the art for automated conformational sampling, offering a successful compromise between energetic accuracy, robustness, and computational cost. However, the evaluation of energies and forces remains a major part of the total search cost, and the scaling of semiempirical electronic-structure calculations increasingly limits the length and number of trajectories that can be performed as molecular size grows.^13^ These limitations motivate potential energy models that preserve or improve conformational accuracy while reducing the cost and size dependence of repeated energy and force evaluations.

Recent advances in machine-learned force fields (MLFFs), trained on high-level quantum-mechanical data, offer a promising route to near-*ab initio* accuracy at dramatically reduced computational cost.^14–16^ Modern architectures, including equivariant message-passing models such as SO3krates^17^ or MACE^18^ and large-scale general-purpose models such as SO3LR^19^ or MACE-OFF23,^20^ demonstrate broad chemical transferability across diverse molecular systems. When combined with GPU-accelerated simulation frameworks, these models enable molecular simulations at scales that were previously inaccessible. However, despite their rapid development, MLFFs remain underutilized in automated workflows for conformational sampling and property prediction, particularly in tools designed for ease of use by nonexpert practitioners.

A key application of conformational ensemble generation is the prediction of infrared (IR) spectra, which provides a direct link between theory and experiment. For flexible molecules, the measured spectrum reflects a Boltzmann-weighted average over many interconverting structures, making accurate conformational sampling essential for reliable spectral predictions.^21–23^ In solution environments, this challenge is further compounded by explicit solute–solvent interactions. Microsolvation, i.e., the inclusion of discrete solvent molecules, captures effects such as hydrogen bonding, polarization, and cooperative interactions that are not described by continuum models, and can significantly alter both conformational preferences and vibrational signatures.^24,25^ Constructing physically meaningful microsolvated clusters therefore requires robust algorithms capable of efficiently generating diverse, sterically consistent solvation shells.

Established software packages for conformational sampling, molecular optimization, or molecular dynamics, such as CREST,^8^ ASE,^26^ and AQME,^27^ were developed around semiempirical, quantum-mechanical, or empirical force field potentials. Although these packages can be interfaced with MLFFs, their sampling algorithms, parameter choices, and execution strategies are not designed to exploit the specific characteristics of recent MLFFs. The main computational advantage of MLFFs comes not only from their efficient energy and force evaluations but also from GPU-native execution, parallel evaluation of multiple structures and trajectories, and sampling algorithms adapted and parametrized for MLFF potential energy surfaces.

Here, we present **MARS** (Machine-Learned Force Field F**r**amework for automated conformational sampling, vibrational spectroscopy, and microsolvation), an open-source Python package designed to bridge this gap. MARS provides a unified GPU-accelerated framework for conformational sampling, vibrational spectroscopy, and microsolvation within a single workflow. Built on JAX^28^ for automatic differentiation and just-in-time compilation and leveraging JAX-MD^29^ for efficient molecular dynamics, MARS enables high-performance simulations on both GPUs and standard laptop hardware.

A key feature of MARS is its *model-agnostic* design, allowing the seamless integration of a wide range of machine-learned force fields as well as other potential energy models. This flexibility enables users to readily adapt the workflow to emerging MLFF architectures without modification of the core framework. At the same time, MARS is explicitly designed to be accessible to nonspecialists, providing automated protocols and sensible defaults that lower the barrier to entry for researchers without extensive computational chemistry expertise (a graphical user interface (GUI) is provided for broad use). See Supporting Information (SI) Section S7.

The conformational sampling workflow combines multiple complementary strategies, including (i) metadynamics with adaptive biasing parameters, (ii) relaxation from diverse initial structures, (iii) multitemperature molecular dynamics for enhanced exploration, and (iv) genetic crossing in internal coordinates to promote structural diversity. Vibrational analysis is integrated into the same pipeline and includes automated mode classification via Wilson B-matrix projections, as well as functional group identification using graph-based pattern matching inspired by the AccFG algorithm^30^ (see Section S3.4 of the SI for details).

The remainder of this work is organized around a set of benchmarks and applications designed to test MARS across the main regimes encountered in ensemble-based molecular modeling. We begin by assessing conformer energetics for a chemically diverse collection of molecules, including alkanes, halogenated aromatics, amino acids, peptides, drug-like systems, and phosphamides. This benchmark compares modern MLFFs with semiempirical tight-binding methods and density functional reference calculations, providing a quantitative assessment of relative energy accuracy, optimization behavior, and numerical stability in automated conformer searches.

We next evaluate the spectroscopy, noncovalent sampling, and microsolvation components of the framework. Harmonic IR spectra are benchmarked against experimental gas-phase spectra, while the automated mode-classification workflow is illustrated through chemically assigned vibrational eigenvectors. Larger case studies on alanine peptides and proline/β-cyclodextrin test whether MARS can recover experimentally relevant conformational reorganization, host–guest binding motifs, and ensemble-averaged IR signatures. Finally, explicit microsolvation is examined for alanine dipeptide and 2-methoxycyclohexanone, where solvent-specific interactions drive changes in conformer stability and vibrational response. These examples collectively probe isolated flexible molecules, noncovalent complexes, solvated clusters, and biomolecular systems, establishing the range of chemical problems accessible with the automated MARS workflow.

Together, these benchmarks test the central hypothesis of this work: that modern MLFFs can move automated conformational sampling beyond the established semiempirical CREST/GFN2-xTB paradigm by simultaneously improving the high-level energetic accuracy, computational throughput, and scaling with molecular size. MARS provides the automated, model-agnostic framework required to translate these advances in MLFFs into practical conformational ensemble generation, spectroscopy, and microsolvation workflows.

## 2 Overview of the MARS Framework

MARS integrates conformational sampling, vibrational spectroscopy, and explicit microsolvation into a single automated workflow. The package is organized as a set of composable modules that share a common data model, configuration interface, and potential energy back end. This design allows a researcher to proceed from a Cartesian input structure to a solvated, ensemble-averaged spectrum without manually coordinating intermediate steps. Parameters that would otherwise require expert selection, including bias strengths, simulation lengths, convergence thresholds, and refinement seeds, are estimated from the molecular topology using internal heuristics. Three preset profiles (quick, normal, and thorough) provide an accessible cost–accuracy trade-off. The main components of the framework are summarized below; complete methodological and implementation details are provided in Sections S1–S6 of the SI.

### 2.1 Conformational Sampling

The conformational workflow forms the backbone of MARS (Figure 1A). Its design is inspired by the automated conformer–rotamer search strategy introduced in CREST,^8^ particularly its combination of biased molecular dynamics, ensemble filtering, and genetic recombination. MARS implements these concepts as an independent Python/MLFF workflow designed for accelerator-native sampling and subsequent spectroscopic analysis.

**Figure 1:**
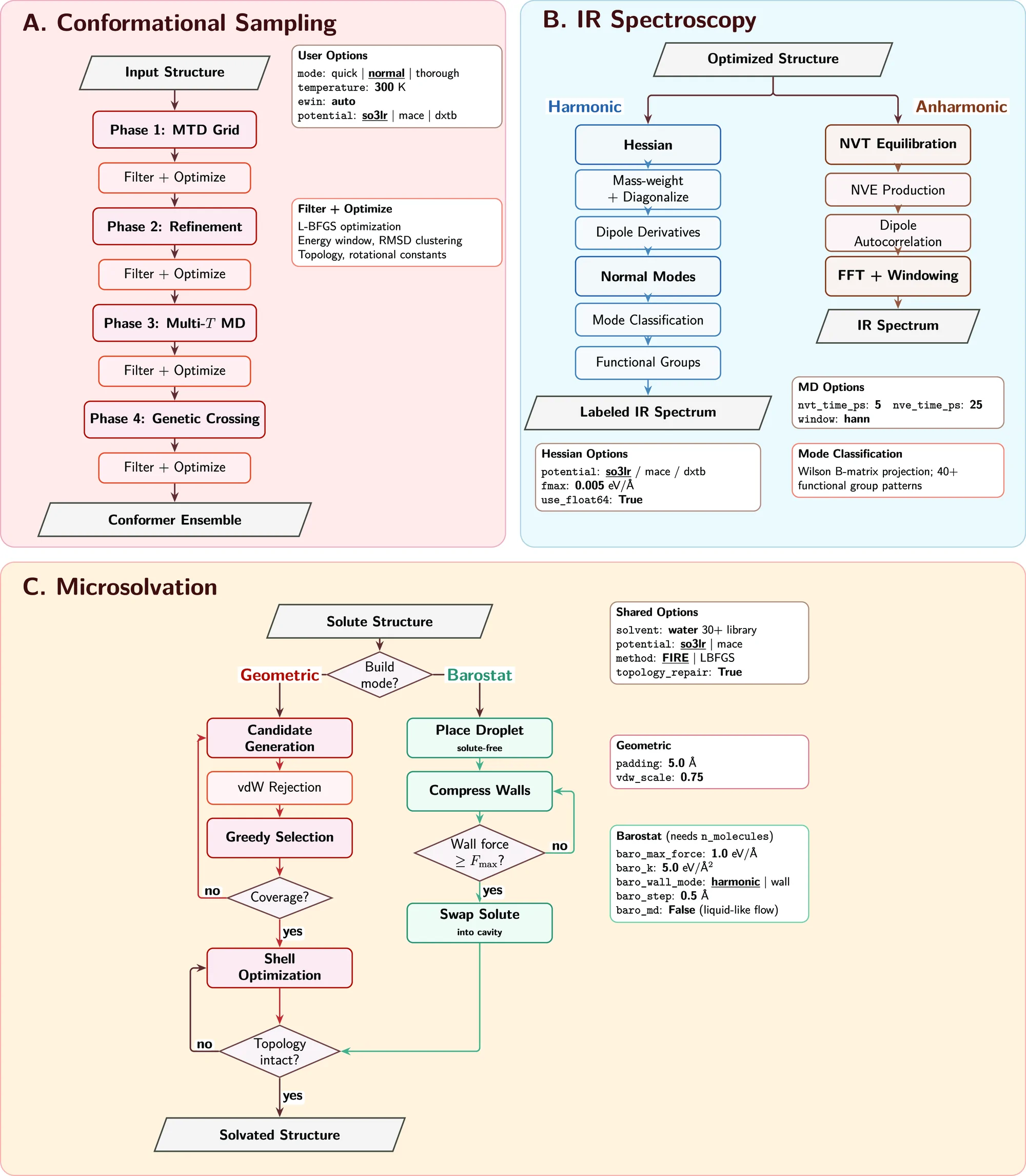
Overview of the principal MARS workflows. (A) Conformational sampling combines parallel metadynamics, seed refinement, multitemperature molecular dynamics, and genetic crossing, with intermediate filtering and L-BFGS optimization. (B) IR spectra are calculated using either analytical Hessians and normal-mode assignment or molecular dynamics followed by dipole autocorrelation and Fourier analysis. (C) Microsolvation proceeds through either geometric shell construction (red) or droplet compression and solute insertion (teal), followed by a common covalent-topology check. Shapes distinguish computational operations, inputs and outputs, and decision points. Cream boxes indicate key user-selectable settings, with default options shown in bold. All workflows use a model-agnostic potential interface supporting SO3LR, MACE, and dxtb.

Starting from a Cartesian structure in .xyz format, the workflow explores configuration space through four complementary stages: (I) *a parallel metadynamics, using RMSD as collective variable grid, spanning multiple bias amplitudes and widths;* (II) *refinement trajectories initialized from structurally diverse metadynamics intermediates;* (III) *unbiased molecular dynamics at multiple temperatures to capture rotameric transitions;* and (IV) *genetic crossing of dihedral angles in internal coordinates.* A detailed description of the sampling algorithms, simulation parameters, collective variables, and selection criteria is provided in Section S1.4 of the SI.

Between stages, candidate structures are optimized and filtered using energy windows, RMSD clustering, rotational-constant similarity, and covalent-topology validation (see Section S1.6 of the SI). Consequently, only chemically valid and nonredundant structures are propagated. Multifragment noncovalent systems are treated by detecting connected components and activating fragment-aware confinement and permutation-invariant structural comparisons, thereby reducing dissociation and avoiding redundant configurations without requiring manual setup (see Section S1.6.5 of the SI).

### 2.2 Vibrational Spectroscopy

The spectroscopy module converts individual structures or conformational ensembles into predicted IR spectra (Figure 1B). Where supported by the selected back end, harmonic frequencies are obtained from analytical mass-weighted Hessians. Normal modes are characterized by projection onto an internal-coordinate basis and assigned chemically interpretable labels, such as carbonyl stretching, amide bending, and methyl rocking, using a graph-based functional group identification procedure^30^ (see Section S3.1 of the SI). The framework also supports molecular-dynamics-based spectra obtained from dipole autocorrelation functions, enabling harmonic and finite-temperature spectroscopic analyses within the same interface (see Section S3.3 of the SI).

### 2.3 Explicit Microsolvation

The microsolvation module constructs solvent environments around isolated molecules, noncovalent complexes, and conformational ensembles (Figure 1C). Two complementary construction strategies are available:

- The default geometric builder distributes candidate solvent positions on Fibonacci spheres, rejects steric overlaps, and balances solute proximity with angular coverage while growing relaxed shells outward from the molecular surface (see Section S4.2 of the SI).
- The barostat-based builder compresses a solvent droplet within a spherical moving wall and subsequently introduces the solute into a reserved central cavity, producing compact, liquid-like environments (see Section S4.7 of the SI).

A library of more than 30 preoptimized solvents is included (see Section S4.3 of the SI). Solvent shells can also be transferred between related conformers, and solvent counts can be automatically standardized across an ensemble to facilitate batch calculations.

### 2.4 Model-Agnostic Potential Interface

MARS is designed to operate independently of the underlying potential energy model. The package defines a minimal model interface and provides adapters for SO3LR,^17,19^ MACE^18^ foundation models, including MACE-OFF23,^20^ and the semiempirical dxtb^31^ back end. Thus, while MARS is model-agnostic at the interface level, its current high-performance execution requires a JAX-compatible implementation of the underlying potential. Models implemented in other frameworks can in principle be interfaced through alternative GPU-based workflows, although with reduced performance compared to the fully JAX-based execution path. Changing the potential therefore requires only a configuration-level modification. Additional models can be incorporated by implementing a single adapter without altering the sampling, optimization, or analysis modules. A full validation of the implemented potentials and the timings with respect to ASE implementation is in Section S8 of SI.

## 3 Conformational Exploration

The practical success of conformational sampling is fundamentally determined by the cost and accuracy of the underlying potential energy model. Reliable exploration of flexible drug-like molecules typically requires tens of thousands of energy and force evaluations across enhanced-sampling and molecular dynamics trajectories, which has historically limited routine workflows to semiempirical quantum chemistry methods. In particular, GFN2-xTB^11^ has emerged as a widely adopted standard through its integration into conformer-search tools such as CREST,^8^ offering an effective compromise between computational efficiency and approximate DFT-level conformational energetics.

Recent general-purpose machine-learned force fields (MLFFs) provide an alternative route toward scalable conformational sampling. General-purpose models such as SO3LR^17^ and MACE-OFF23 ^20^ provide fully differentiable force evaluation and native GPU acceleration, making them particularly attractive for large-scale ensemble generation. The central question addressed here is therefore whether current MLFFs can provide a reliable and computationally competitive alternative to semiempirical potentials for conformational sampling.

We benchmark the MLFFs against the semiempirical GFN1-xTB and GFN2-xTB baselines, evaluated using dxtb. Two SO3LR generations are tested. SO3LR-1 is the published release,^19^ and SO3LR-2 (2602) is the new one. SO3LR-2 is a general-purpose machine-learned force field for (bio)­molecular simulations, available in small, medium, and large variants (SO3LR-2-S/M/L). The models are trained on ∼150 million structures spanning 85 elements, drawn from several quantum-chemical data sets^42–48^ and combined through a multihead scheme. From the MACE family, we evaluate the small and medium variants of MACE-OFF23,^20^ together with MACE-OFF24 and MACE-OMol, a larger model trained on OMol25.^46^

To assess the suitability of MLFFs for conformational exploration, particularly in applications commonly addressed using semiempirical methods, we assembled a two-tier benchmark encompassing both realistic conformational-search tasks and targeted energetic tests (Table 1). The conformational-search systems considered in this work span 14–116 atoms and are therefore relatively small compared with many practical drug-like applications. This size range reflects the high computational cost of generating consistent quantum-chemical reference geometries and relative energies, particularly at correlated wave function and high-level DFT levels, for large conformational ensembles. Nevertheless, such systems provide a valuable high-accuracy benchmark because their conformational energetics can be evaluated against well-defined, high-level reference calculations. The first tier comprises 36 chemically diverse molecules and 454 reference conformers. These systems span a broad range of molecular sizes, flexibilities, and chemical complexity, from rigid alkanes to flexible peptides,^32,33,35,36^ drug-like MPCONF196 ^37^ systems, and stereoelectronically challenging phosphamides.^38^ Each subset is evaluated against the highest-level reference data available from the corresponding study; full descriptions and references are provided in the SI, Section S9. This tier evaluates the ability of the methods to explore conformational space and reproduce the relative energetics of relevant conformers across chemically diverse systems.

**Table 1:** Benchmark Data Sets Used in This Work^a^

Data set	Systems	Conformers	Reference level
*Curated high-level set – conformational search*			
Linear/branched alkanes	11	183	CCSD(T)/cc-pVTZ^32^
Cyclohexane	1	4	CCSD(T)/CBS^33^
Difluorobenzaldehydes	4	7	DLPNO–CCSD(T)/def2-TZVP^34^
Free amino acids	4	46	CCSD(T)/Aug-cc-pvtz or 6–311++G(d,p)^35^
Alanine dipeptide (Ala_2_)	1	6	MP2/aug-cc-pVQZ^36^
MPCONF196	13	196	(DLPNO−)CCSD(T)/CBS^37^
Phosphamides	2	12	B3LYP/6–31+G**^38^
*Public reference sets – single-point accuracy*			
TorsionNet500	500	12 000	B3LYP^39^/PBE0+MBD^19^/ωB97M-D3(BJ)/def2-TZVPPD^20^
CCCBDB torsion scans	37	887	Experiment^40^ (+ PBE0+MBD)
QUID (dimers)	42	42^b^	CCSD(T), PBE0+MBD^41^

a The curated high-level set drives the full conformational-search benchmark (ensemble generation, optimization, geometry retention, and sampling recovery); the public reference sets probe single-point relative energy accuracy at scale. Per-subset references and full descriptions are given in the Supporting Information (Section S9).

b Dimer interaction energies (one per system) and not relative conformer energies.

The second tier provides targeted tests of energetic contributions that are particularly important for the conformational sampling. We evaluate single-point relative conformer energies on TorsionNet500,^39^ torsional barriers on the CCCBDB set,^40^ using PBE0+MBD and experimental references, and noncovalent interaction energies for 42 dimers from QUID,^41^ referenced to CCSD­(T) and PBE0+MBD. These complementary tests probe the description of conformational energy differences, torsional energetics, and intermolecular interactions, thereby helping to identify the capabilities and limitations of each model for the conformational exploration.

### 3.1 Energetic Accuracy

Before performing the full conformational searches, we first assessed the intrinsic energetic accuracy of each potential through single-point (SP) calculations on fixed, high-level reference geometries. By exclusion of method-dependent geometry relaxation and sampling effects, this analysis isolates the ability of each potential to reproduce reference energy differences for a common set of molecular structures. Although SP performance does not by itself determine the outcome of a conformational search, it provides an initial measure of the energetic fidelity of each model and helps to identify errors that may affect the ranking of sampled conformers.

Figure 2 summarizes the SP energy errors across the ten relevant benchmark subsets and reveals clear differences among the model families. The two semiempirical methods exhibit markedly different performances. GFN1-xTB is generally the least accurate method, whereas GFN2-xTB provides a substantially stronger and more robust baseline with an accuracy comparable to that of several MLFFs across most subsets. It should be mentioned, however, that the GFN methods were designed with an emphasis on properties around energetic minima, including molecular geometries, vibrational frequencies, and noncovalent interactions, rather than on highly accurate absolute or relative energies alone.^10,11^ Among the MLFFs, SO3LR-2 consistently improves upon SO3LR-1, demonstrating the progress achieved between the two model generations. The comparatively larger errors of SO3LR-1 can be understood in the context of its original design: it was trained primarily on forces for molecular dynamics applications using a data set of approximately 4 million structures, compared with approximately 150 million structures for SO3LR-2. SO3LR-1 was therefore not specifically optimized for highly accurate relative conformer energies across a broad chemical space. The improvement is particularly pronounced for the phosphamide and QUID subsets for which SO3LR-2 largely resolves the comparatively large errors observed with SO3LR-1. For the chemically and conformationally challenging MPCONF196 and phosphamide subsets, SO3LR-2 and the MACE models outperform both of the semiempirical methods. The strongest overall performance is obtained with MACE-OMol, which is the most accurate method for the majority of the benchmark subsets and maintains consistently low errors for both the intramolecular conformer energies and intermolecular interaction energies. However, MACE-OMol is also the largest and computationally most expensive model evaluated here, highlighting the trade-off between the energetic accuracy and computational throughput. These results show that the most recent general-purpose MLFFs no longer merely approach semiempirical accuracy: for the most chemically and conformationally demanding subsets, they provide a systematic improvement over both GFN1-xTB and GFN2-xTB. This gain is particularly significant for conformational sampling because errors in small relative energy differences directly affect the conformer ranking and the composition of the predicted ensemble.

**Figure 2:**
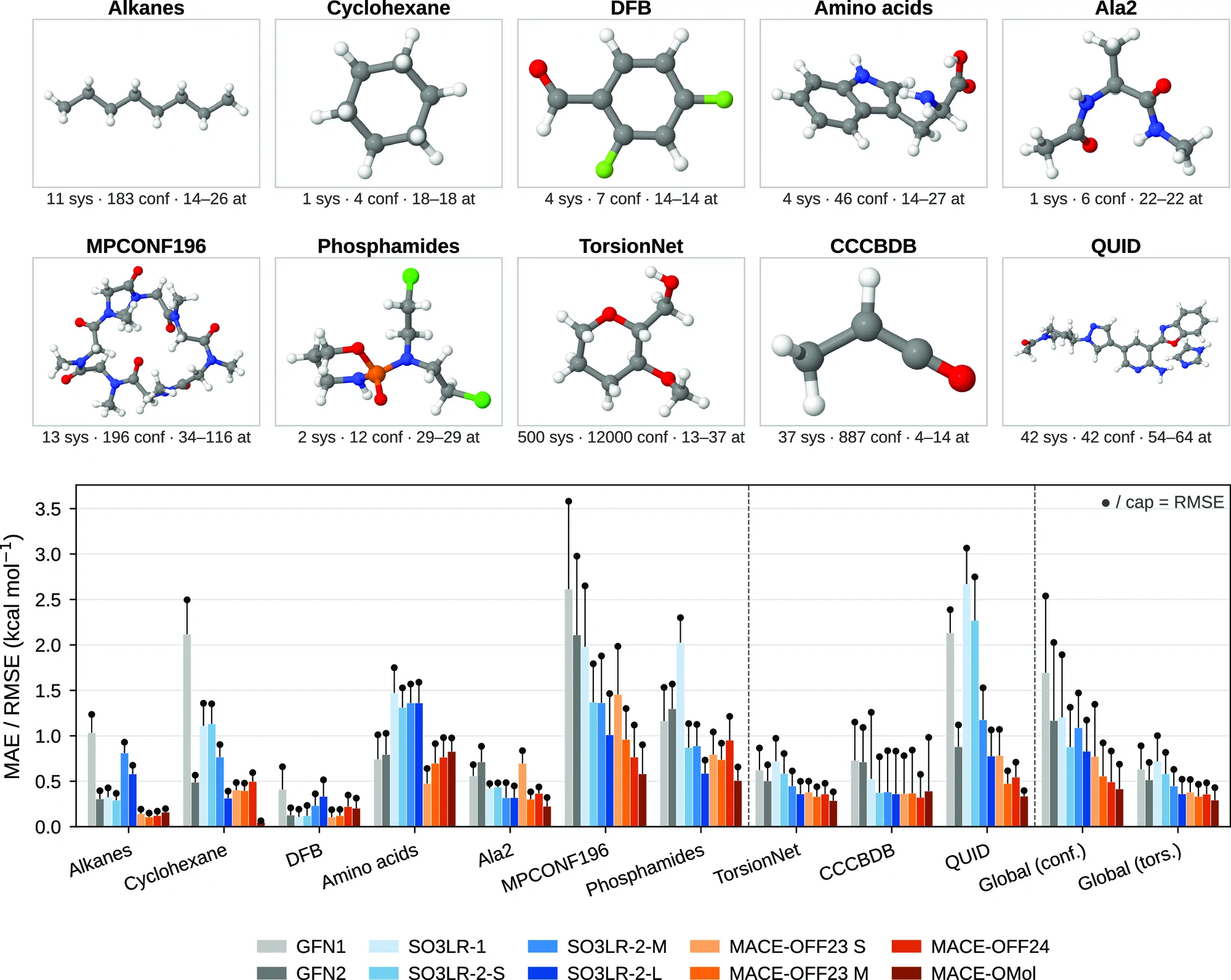
Single-point accuracy of the MARS implemented potentials across all benchmark families. **Top:** a representative structure of each subsystem, annotated with the number of systems, conformers, and atom range. **Bottom:** mean absolute error (bars) and RMSE (dots/caps) of each potential per subsystem on a common linear scale; the dashed line separates the curated conformer-energy sets (left) from the external single-point sets (right). The two rightmost columns report *n*-weighted global metrics pooled over the curated conformer-energy subsets (“Global (conf.)”) and over the torsion/dimer sets (“Global (tors.)”). Full per-reference values are given in SI Tables S16–S22.

We next evaluate the complete conformational-sampling workflow, where differences between methods may arise both from the potential energy surface and from the ability of the search protocol to explore it. Performance is characterized using three complementary metrics (Figure 3). Minimum retention is the fraction of reference conformers that remain distinct minima after unconstrained optimization with each potential, using a matching threshold of 0.3 Å Kabsch heavy-atom RMSD. For the retained conformers, energy accuracy is quantified by the MAE and RMSE of the relative energies with respect to the highest-level literature reference available for each subset. Finally, sampling recovery measures the fraction of the distinct minima supported by each potential that are located by the corresponding MARS search. Thus, retention tests whether a potential preserves the reference minima, whereas recovery tests whether the sampling procedure finds the minima present on that potential energy surface.

**Figure 3:**
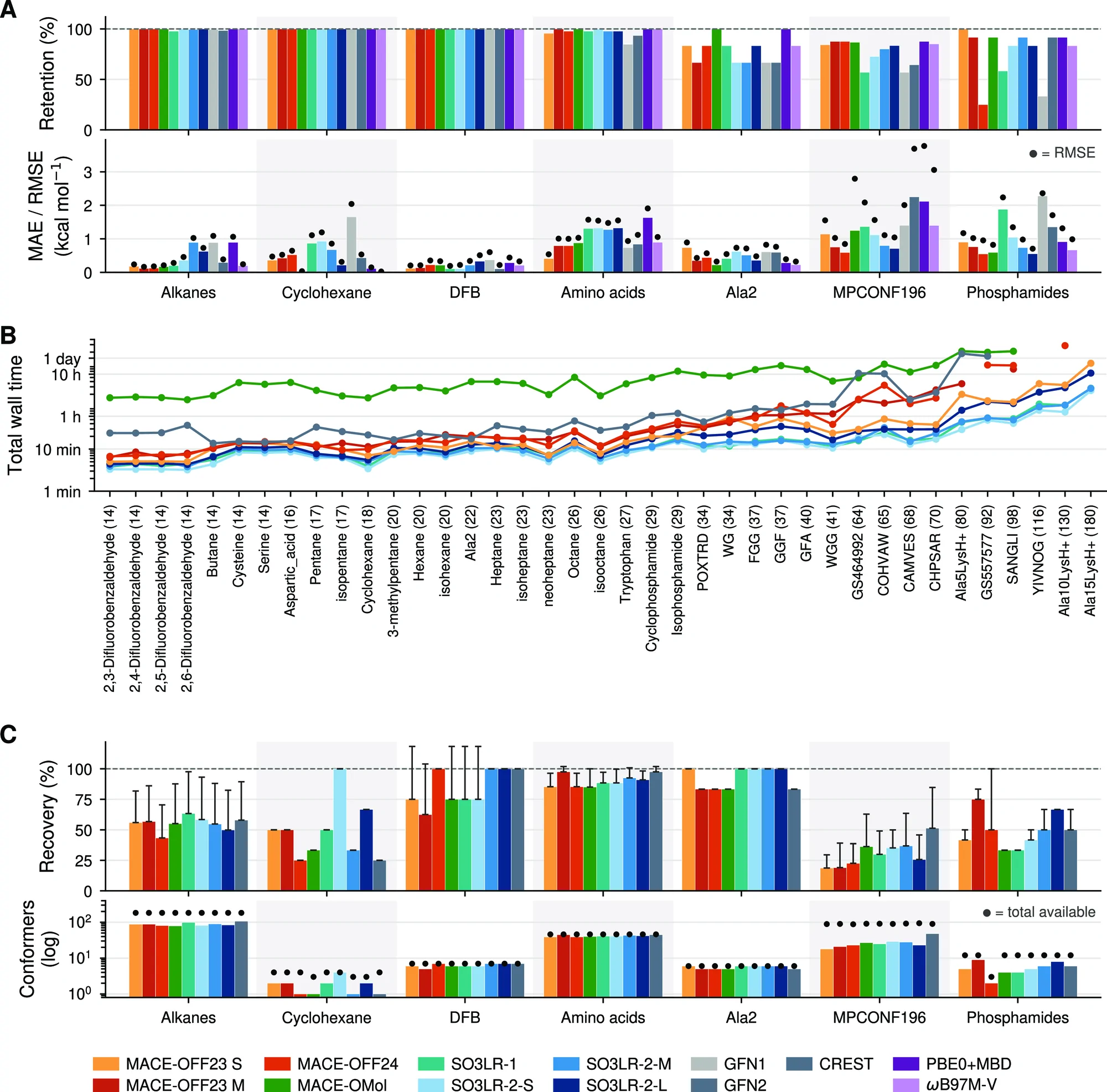
Benchmark of MARS with MACE-OFF23 (small/medium), MACE-OFF24, MACE-OMol, and SO3LR-1 and SO3LR-2-S/M/L against literature and DFT references on the seven-subset test set. (A) Minimum retention (fraction of reference conformers within 0.3 Å Kabsch heavy-atom RMSD after unconstrained optimization) and retained-set relative energies against the highest-level published reference per subset. Bars represent the MAE, while black points represent the RMSE. (B) Mean wall time per conformer as a function of molecular size. MLFF calculations were performed on a single NVIDIA V100 16 GB GPU at the University of Luxembourg HPC facility using double precision (--float64). CREST/GFN2-xTB calculations used 28 CPU cores, a 0.5 fs integration time step, and no SHAKE constraints. Calculations exceeding 48 h or failing because of memory limitations were classified as incomplete. (C) Sampling recovery: top, per-molecule mean ± std of the fraction of each method’s own optimized minima that are matched within 0.3 Å Kabsch heavy-atom RMSD; bottom, the corresponding absolute number of recovered conformers (bar) and total number of low-energy reference minima available (black point), summed over every molecule in the data set. Points and error bars denote the mean and standard deviation, respectively, over the molecules in each subset.

### 3.2 Minimum Retention and Conformer Energies

Minimum retention and conformer energies are summarized in Figure 3A. Nearly all reference conformers are retained for the simpler subsets, while MPCONF196 and the phosphamides provide greater discrimination among the methods. The differences between SO3LR-1 and SO3LR-2 are most pronounced for the phosphamides: the SO3LR-2 models retain substantially more reference conformers and largely eliminate the deficiencies observed for v1. Among the retained conformers, the MACE models generally yield the lowest relative energy errors, with MACE-OMol providing the strongest overall performance; SO3LR-2-L is the most accurate member of the SO3LR family. For some of the most challenging systems, the disagreement between the two DFT reference levels is comparable to the residual MLFF errors (SI Figures S4 and S5), suggesting that differences of this magnitude should be interpreted in the context of the uncertainty associated with the reference method. Full per-data set values are given in SI Table S19.

### 3.3 Total Wall Time

The timing results in Figure 3B show that the SO3LR models are consistently the fastest and exhibit the most favorable scaling with molecular size. Notably, the improvements of SO3LR-2 are obtained without a substantial loss of efficiency, relative to SO3LR-1. The cost of the MACE and CREST/GFN2-xTB workflows increases more strongly for larger systems, and some calculations exceed the 48 h wall time or available-memory limits. In contrast, the SO3LR-based searches finish within these limits for all of the systems considered. These timings represent practical workflow costs under the stated GPU and CPU resources rather than hardware-normalized method comparisons.

### 3.4 Sampling Recovery

Sampling recovery (Figure 3C) is high for the simpler subsets but decreases for MPCONF196 and the phosphamides. The top panel quantifies the fraction of reference minima recovered by each method, while the bottom panel reports the absolute number of recovered reference conformers and the total number of conformers identified during the sampling. This comparison accounts for additional minima discovered during sampling that are not present in the reference set and therefore do not contribute to the relative recovery metric. SO3LR-2 improves recovery relative to SO3LR-1, particularly for the phosphamides, although no model achieves the highest recovery across all subsets. Overall, SO3LR-2 provides the most favorable balance of retention, sampling recovery, energy accuracy, and computational throughput, whereas MACE-OMol offers the highest energetic accuracy at a substantially greater cost.

### 3.5 Numerical Stability and Reproducibility

Numerical stability and reproducibility were additionally assessed using ten independent replicas for five systems containing 14–70 atoms (SI Section S10). GPU calculations can exhibit small run-to-run numerical differences due to floating-point operations. Although these perturbations are initially negligible, they may be amplified during geometry optimization or molecular dynamics and lead nominally identical conformational searches to different minima. Reproducibility is therefore an important practical measure of the numerical stability of each model and implementation. Under the conditions tested, SO3LR produces reproducible results in both single and double precision. In contrast, MACE-OFF23 and MACE-OFF24 are sensitive to single-precision arithmetic, with replicate calculations producing instabilities and divergent sampling outcomes. MACE-OMol exhibits intermediate behavior, remaining stable for the smaller systems but showing single-precision instability for the largest system. These results indicate that single precision is adequate for SO3LR, whereas double precision is recommended for production conformational sampling with the MACE models. For systems exhibiting run-to-run variability, numerical instabilities, or optimization difficulties, calculations can be repeated using the --float64 option to improve numerical stability and convergence.

Although SO3LR-2 provides the strongest accuracy-to-cost balance in this benchmark, it remains an unpublished model under active development. We therefore use the published SO3LR-1 potential for the production calculations reported below, prioritizing public availability, computational efficiency, and reproducibility.^19^

### 3.6 Noncovalent Sampling

We evaluated the ability of the force fields available in MARS to describe noncovalent interaction energetics using NCI-ATLAS,^52,53^ a subset of 20 biologically relevant hydrogen-bonded dimers, including neutral and ionic O/N/C–H donors and halogen contacts, with 406 conformers in total (see Section S11.1 of the SI). For each dimer, conformational ensembles were generated with SO3LR-1, and relative energies, Δ*E*, were referenced to the lowest-energy conformer of that dimer. To separate errors in the potential energy surface from differences in optimized geometries, all methods were evaluated as single points on the same SO3LR-1-generated geometries. SO3LR models were compared with their reference level, PBE0+MBD, whereas MACE-OFF23 small, MACE-OFF23 medium, MACE-OFF24, and MACE-OMol were compared with ωB97M-V. The MACE-OFF models were trained on a subset of the SPICE data set^54^ containing only neutral molecules, whereas SO3LR and MACE-OMol training sets included charged systems. Thus, a poor performance of MACE-OFF models is expected for non-neutral molecules. The two DFT reference levels agree closely, with MAE = 0.56 kcal mol^–1^ and Pearson *r* = 0.98, providing a baseline for the force field errors. The full per-model single-point statistics, the optimized-geometry RMSD analysis, and the corresponding tables are reported in the SI (Section S11).

Figure 4 compares the force field relative energies with the corresponding DFT references. Accuracy improves with model capacity and training-set coverage: the retrained SO3LR-2 variants reduce the legacy SO3LR-1 error by a factor of 1.6–2.7 (MAE 0.72, 0.58, and 0.43 against 1.16 kcal mol^–1^), and sharply improve ranking fidelity, MACE-OMol reaches near-DFT agreement, and MACE-OFF23 medium, MACE-OFF24, and SO3LR-2-L approach the intrinsic uncertainty between the two DFT references. MACE-OFF24 (MAE = 0.66 kcal mol^–1^) improves substantially over MACE-OFF23 small (0.92 kcal mol^–1^) and is comparable to the SO3LR-2 S/M variants, although it does not reach the accuracy of MACE-OFF23 medium or SO3LR-2-L. The remaining errors are broadly distributed across interaction motifs rather than localized to a single hydrogen-bond or halogen-contact class.

**Figure 4:**
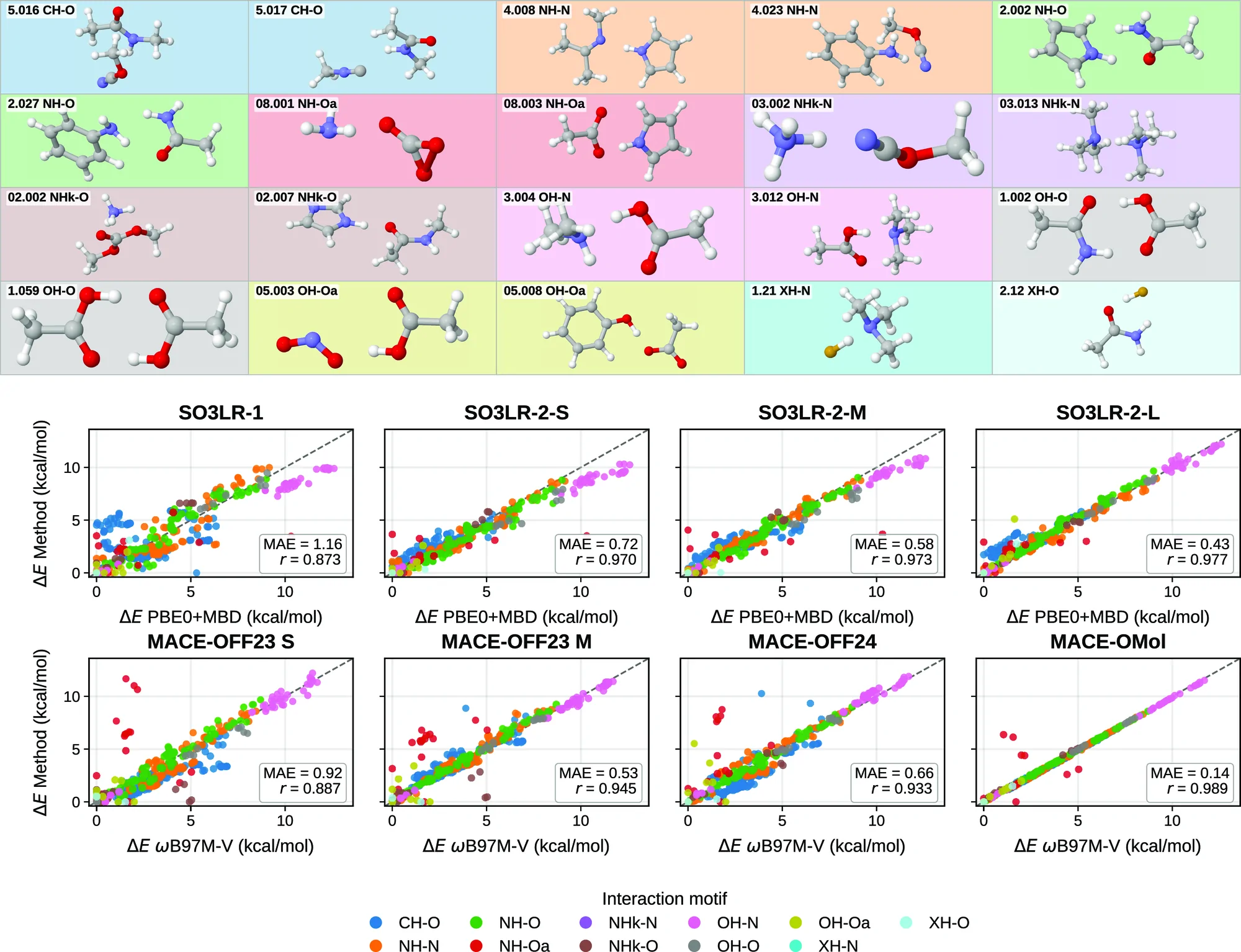
Relative conformer energies, Δ*E*, of the force fields available in MARS, evaluated as single points on common SO3LR-1-generated geometries for the NCI-ATLAS subsets (20 dimers, 406 conformers) and compared with the corresponding DFT reference level: the SO3LR models (legacy SO3LR-1 and the SO3LR-2-S/M/L variants) against PBE0+MBD, and the MACE models (MACE-OFF23 S/M, MACE-OFF24, and MACE-OMol) against ωB97M-V/def2-TZVPD. Points are colored by interaction motif, and the dashed line denotes perfect agreement. Mean absolute errors and Pearson correlation coefficients are reported in each panel.

The same ordering holds for the optimized geometries: relaxing the SO3LR-1 ensembles with each force field and measuring the heavy-atom RMSD to the DFT-optimized minima, SO3LR-2-L and MACE-OMol reproduce the DFT minima essentially within the ωB97M-V–PBE0+MBD baseline, while the legacy SO3LR-1 deviates the most. Per-model errors, correlation coefficients, and the optimized-geometry RMSD analysis are reported in SI Section S11.

These results support a hierarchical use of the models in MARS. SO3LR (particularly the fast −2-S variant) provides efficient, rapid exploration of conformational space, whereas the higher-capacity models (SO3LR-2-L, MACE-OMol) provide more accurate final energetics and geometries. Accordingly, the most effective protocol is to generate ensembles with a fast SO3LR variant and then rerank and, if needed, reoptimize the selected conformers with a higher-accuracy model.

## 4 Infrared Spectrum in Biomolecules

IR spectroscopy probes the vibrational degrees of freedom of a molecule and remains one of the most accessible structural fingerprints available to chemists.^55^ In the harmonic approximation, the molecular vibrational manifold reduces to a set of 3*N* – 6 (or 3*N* – 5 for linear molecules) normal modes whose frequencies ν_*k*_ are the square roots of the eigenvalues of the mass-weighted Hessian, and whose nuclear displacement patterns are encoded in the corresponding eigenvectors **Q**_*k*_.^56^ The IR activity of a mode follows from the dipole-moment derivative along **Q**_*k*_,

$$I_{k}^{\mathrm{IR}}\propto {\left|\frac{\partial \mu }{\partial Q_{k}}\right|}^{2}$$

so an accurate IR spectrum requires both reliable vibrational frequencies and a reliable description of the molecular dipole surface—two complementary tests of an electronic-structure method or force field.

Beyond reproducing peak positions, the eigenvectors themselves carry chemical information. Associating an experimental band with a specific normal mode—the C = O stretch of a ketone, the umbrella mode of a methyl group, the in-plane bend of an amide N–H—relies on inspecting **Q**_*k*_ and verifying that the atomic motion matches the diagnostic pattern. This eigenvector association underpins assignments in synthetic chemistry, mechanistic studies of catalysis, conformational analysis of biomolecules, and the interpretation of atmospheric- and surface-science IR data.^57–59^ Among the models currently implemented in MARS, the calculation of IR intensities is restricted to potentials that predict partial charges or molecular dipole moments, which currently include the SO3LR family and dxtb.

Methanol was selected as a representative experimental case study to demonstrate the automatic assignment capabilities of MARS-IR (Figure 5A). Beyond generating broadened IR spectra, MARS-IR retains the scaled frequencies, intensities, and Cartesian eigenvectors of all normal modes, enabling each spectral feature to be linked directly to a molecular displacement pattern without manual inspection of Hessian outputs. For methanol, the dominant bands are assigned automatically to the C–O stretch, CH_3_ deformations, C–H stretches, and the high-frequency O–H stretch. This mode-resolved representation allows experimental spectra to be interpreted at a glance, separating genuine frequency shifts from intensity changes, band overlap, or normal-mode mixing. The SI (see Section S3.7) illustrates the associated visualization workflow: --plot displays the calculated and experimental IR spectra on the same frequency grid, whereas mars viewer --ir opens the normal-mode viewer for inspecting individual frequencies, intensities, and eigenvector animations. MARS-IR therefore provides an experimentally oriented interface for converting computed vibrational spectra into chemically interpretable band assignments.

**Figure 5:**
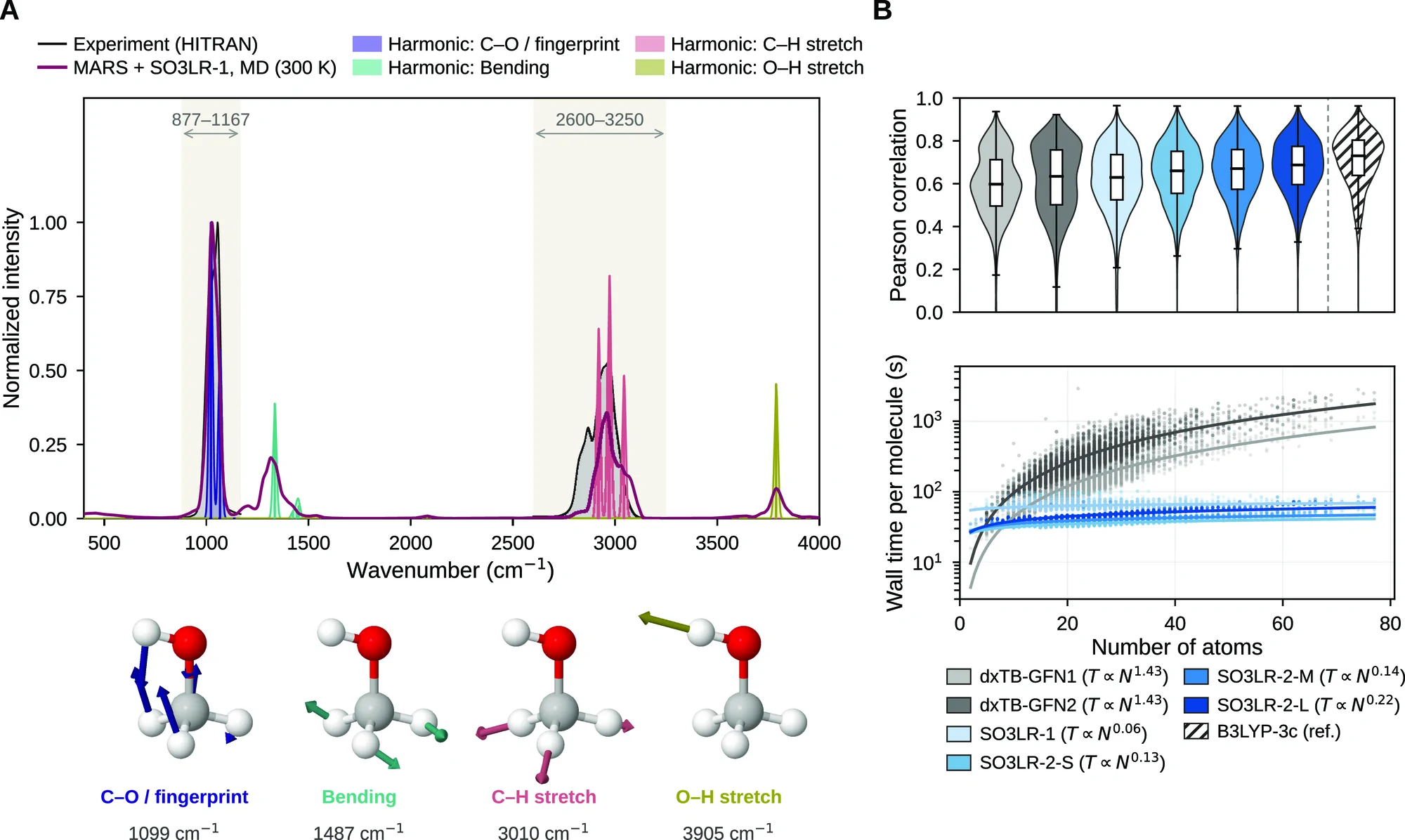
Benchmark of MARS-IR approaches (A) **Top:** Gas-phase IR spectrum of methanol from experiment and from the MARS-SO3LR-1 machine-learned force field. The black curve is the experimental HITRAN^49^ absorption cross section, available only in the two yellow-shaded spectral windows, 877–1167 cm^–1^ and 2600–3250 cm^–1^, corresponding to the C–O/fingerprint and C–H stretching regions, respectively. The red curve is the predicted anharmonic (MD) spectrum, scaled by 0.97. Harmonic spectra are colored by vibrational character. **Bottom:** Representative MARS-SO3LR vibrational eigenvectors for methanol: mixed C–O/fingerprint motion at 1099 cm^–1^, H–C–H asymmetric bending at 1487 cm^–1^, symmetric C–H stretching at 3010 cm^–1^, and O–H stretching at 3905 cm^–1^. (B) **Top:** SO3LR-1, the three SO3LR-2 model sizes (S/M/L, all FD), dxtb-GFN1 FD, and dxtb-GFN2 FD against experimental spectra from the NIST35 data set. B3LYP-3c data from Pracht et al.^50^ are included in gray as an external reference. Violin plots of the shift-tolerant Pearson correlation coefficient as in.^51^ Inner boxplots indicate the median and interquartile range. Statistics are reported for the 4155 molecules common to all six methods using a scaling frequency factor of x0.97. **Bottom:** Wall time per molecule as a function of the number of atoms. Solid lines denote power-law fits, obtained from linear regression of log *T* against log *N*; the fitted exponents are reported alongside each method in the shared legend. B3LYP-3c is omitted because timing information is unavailable.

We next benchmarked MARS-IR on the molecular set assembled by Pracht et al.,^50^ originally designed to compare low-cost methods against the NIST/EPA gas-phase IR archive. The benchmark consists of a subset of 4155 molecules with experimentally measured gas-phase IR spectra available in NIST35,^60^ and it was used consistently across all methods.

Harmonic vibrational spectra were compared following our previous work^51^ (Figure 5B). The dxtb fallback implementation in MARS does not support analytical Hessian evaluation; therefore, for consistency, finite differences were used. A comparison of the computational timings and numerical differences between analytical and finite-difference SO3LR Hessians is provided in SI Section S12.1.3. Across the NIST35 subset, the SO3LR models reproduce experimental gas-phase spectra with a shift-tolerant correlation approaching the hybrid-DFT (B3LYP-3c) reference, improving monotonically from the original SO3LR to the SO3LR-2-L model; dxtb-GFN2 is on par with the original SO3LR-1, and dxtb-GFN1 is the least accurate. The two families scale very differently with system size: the fitted exponent for the dxtb variants is approximately 1.43, whereas the SO3LR timings vary only weakly with the system size over this range. These exponents should not be interpreted as asymptotic theoretical scaling, as the reported values correspond to the total MARS workflow, including hardware initialization, geometry optimization, and Hessian construction. For these relatively small molecules, fixed GPU initialization and allocation overheads constitute a substantial fraction of the SO3LR runtime, masking the underlying size dependence of MLFF evaluation. Combined with its near-DFT correlation, this sublinear scaling identifies the SO3LR-2-L model as the sweet spot for high-throughput harmonic IR screening of medium-to-large organic molecules. Median correlations, interquartile ranges, and fitted scaling exponents for every method are reported in SI Section S12. A comparison to the anharmonic (MD) approach for a diverse subset of 100 molecules is provided in Section S12.1 of SI.

## 5 Solvation Effects in Biomolecules

We assessed the MARS microsolvation workflow on two systems whose conformational equilibria are governed by qualitatively different solute–solvent physics. The first is alanine dipeptide (Ace-Ala-NMe), a canonical flexible-backbone benchmark whose six low-lying conformers (C7eq, C5, C7ax, β_2_ is optimized with constrained dihedrals because it is not a minimum in vacuum with SO3LR-1, α_*L*_, α) interconvert on the Ramachandran (ϕ, ψ) surface and whose relative stabilities are strongly modulated by solvent. As a reference, we use ωB97M-V calculations in the gas phase and with the CPCM continuum model for water and diethyl ether, on the benchmark conformer geometries; the MP2 gas-phase and polarizable-continuum-model results reported by Wang and Duan^61^ are included in the SI for comparison. Because a continuum model does not represent explicit solvent structure, directional hydrogen bonding, or solvent configurational sampling, agreement with it is used only to assess whether the explicit-solvent MLFF predicts compatible solvent-induced effects, not as a quantitative benchmark of the microsolvation protocol. The second system is 2-methoxycyclohexanone, whose equilibrium reflects coupling between ring inversion (axial/equatorial) and rotation about the O–C2 bond (*g* ^+^, *g* ^–^, anti). This system has been characterized experimentally by infrared spectroscopy and by B3LYP/6-311+G­(d,p) calculations using the Onsager continuum model.^62^

The protocol is two-stage. First, for each conformer, MARS-SO3LR-1 builds and relaxes the explicit solvent shell: solvent molecules are inserted layerwise with on-the-fly relaxation around a reference conformer (C7eq for the dipeptide, the first conformer of the ensemble for the ketone), the shell is transplanted onto every other conformer (solute swap), and each cluster is then fully optimized. This default geometric layer-by-layer builder was used for all solvation results reported here; the alternative barostat builder is compared with it for alanine dipeptide in SI Section S13.1. The shells comprise 100 H_2_O or 25 Et_2_O molecules for the dipeptide, and 25 CCl_4_ or 25 CH_3_CN molecules for the ketone, with five independent solvation replicas generated per conformer. Second, single-point energies are evaluated on these fixed SO3LR-1-generated geometries with every benchmarked potential (SO3LR-1, SO3LR-2-S/M/L, MACE-OFF23 S/M, MACE-OFF24, and MACE-OMol). The conformational energy of the solute is taken as the sum of its per-atom (embedded) energy contributions. Because these atomic energy contributions depend on the local environment, they implicitly incorporate the influence of the explicit solvent on the solute. This decomposition is model-dependent and does not represent a unique physical partitioning of the total solute–solvent interaction energy; rather, it provides a consistent model-specific measure for comparing the relative stabilization of different conformations evaluated with the same potential.

Both systems reproduce the principal qualitative solvent-induced changes, but the agreement is not uniform across conformers. For alanine dipeptide (Figure 6A), the gas-phase relative energies of SO3LR-1, SO3LR-2-L, and MACE-OMol agree with the ωB97M-V reference to within a mean absolute deviation of 0.04–0.35 kcal mol^–1^. The MP2 PCM results of Wang and Duan,^61^ PBE0+MBD CPCM, and the remaining potentials are given in the SI (Section S13). Evaluated as single points on common microsolvated clusters, the potentials behave consistently, and the largest solvent effect of the continuum-solvation ωB97M-V/def2-TZVPD reference is reproduced: the extended β_2_ backbone, 3.2–3.6 kcal mol^–1^ above C7eq in the gas phase, is lowered to 0.3–0.6 kcal mol^–1^ in water (0.44 kcal mol^–1^ in the continuum DFT), and C5 is lowered more moderately, to 0.7–1.0 kcal mol^–1^ (0.15 kcal mol^–1^ in the continuum DFT). In ether, the mean absolute deviation from the continuum DFT is 0.5–0.7 kcal mol^–1^ for the three potentials shown. In water, it is larger, 1.6–2.8 kcal mol^–1^, because the metastable states are not stabilized: C7ax, α_*L*_, and α lie 1.5–4.8, 3.1–3.4, and 2.2–5.2 kcal mol^–1^, respectively, above the continuum DFT values, α_*L*_ remaining at its gas-phase energy. In ether, α_*L*_ is too high by 0.3–1.0 kcal mol^–1^, whereas C7ax agrees within 0.8 kcal mol^–1^. The larger deviations in water reflect the stronger dependence of the water shell on the solute geometry. Water forms directional hydrogen-bond networks with the exposed amide groups, and each replica samples a different network: the relative embedded energies of a given conformer span 5–11 kcal mol^–1^ across the water replicas, against 3–7 kcal mol^–1^ in ether (SO3LR-1). In both solvents, the microsolvation replicas sample genuinely different first-shell configurations, so the relative energies carry sizable scatter (SD ≈ 1–4 kcal mol^–1^; error bars in Figure 6A), comparable to several of these deviations. For 2-methoxycyclohexanone, explicit solvation makes the equatorial-*g* ^+^ conformer dominant in both CCl_4_ and CH_3_CN, in agreement with the B3LYP reference for the tested potentials within the replica scatter (SI Section S14).

**Figure 6:**
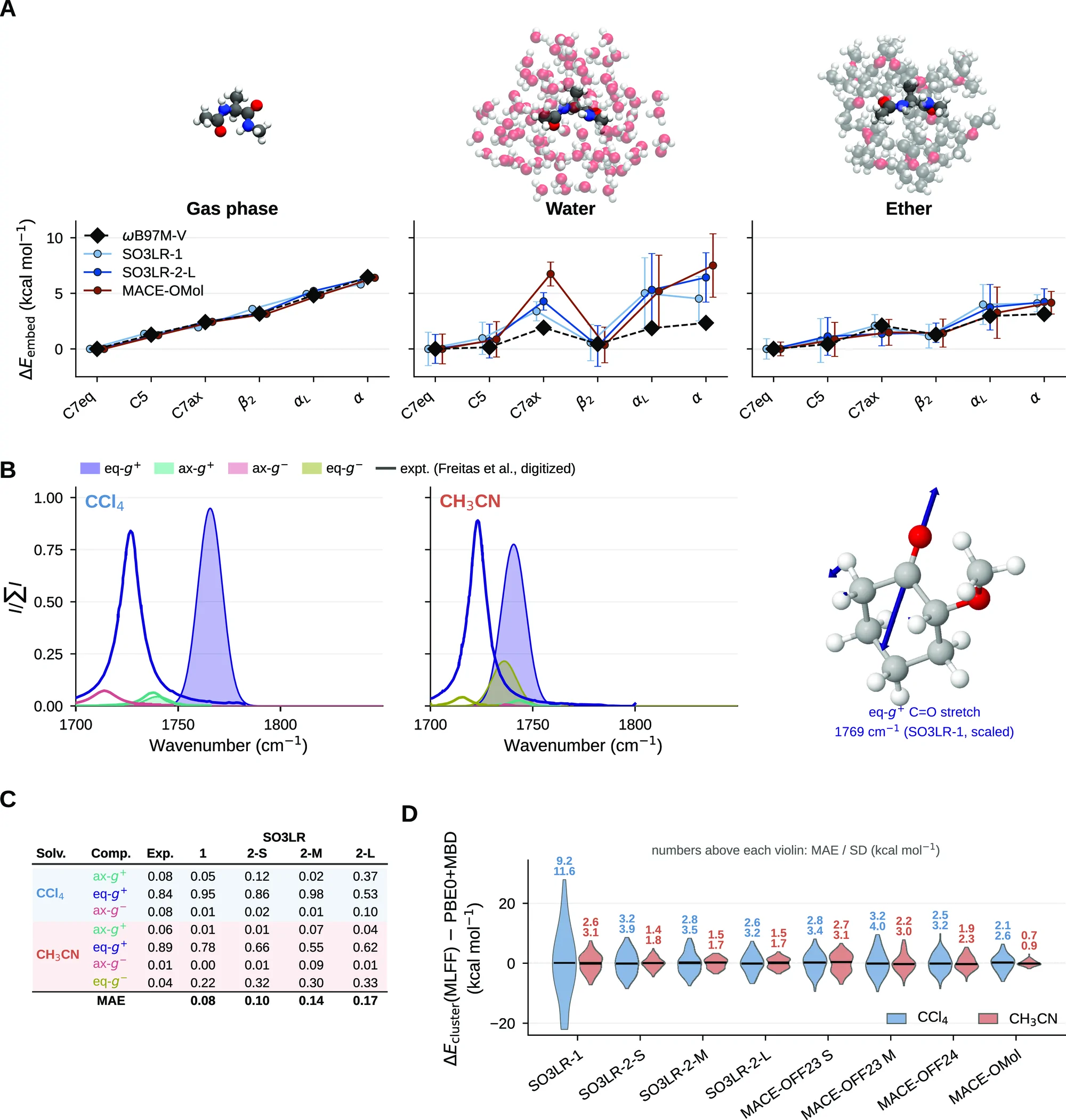
Validation of the MARS explicit microsolvation workflow (SO3LR-1) against reference data. Solvated values are averages over five microsolvation replicas (error bars = SD) computed from per-atom solute energies. (A) Relative solute energies (kcal mol^–1^, referenced to C7eq) of alanine dipeptide conformers in vacuum, water, and diethyl ether, highlighting solvent-induced stabilization of extended backbones;^61^ SO3LR-1, SO3LR-2-L, and MACE-OMol (dots); black diamonds show the ωB97M-V/def2-TZVPD reference (CPCM implicit solvent for water and ether). Solute energies are obtained by summing the MLFF atomic energy contributions assigned to solute atoms. (B) SO3LR-1 relative C = O band intensities (*I*/∑*I*) of the experimentally resolved eq-*g* ^+^, ax-*g* ^+^, ax-*g* ^–^ and eq-*g* ^–^ bands of 2-methoxycyclohexanone in CCl_4_ (left) and CH_3_CN (right): Boltzmann weight times band intensity, mean of five replicas, frequencies scaled by 0.96, Gaussian broadening with fwhm 14 cm^–1^ (peak height = *I*/∑*I*). Lines without a colored area correspond to the digitized experimental component bands from Freitas et al.^62^ Right: eq-*g* ^+^ C = O stretching mode (displacement vectors, isolated conformer). (C) C = O band relative intensities (*I*/*ΣI*) of the resolved carbonyl bands of 2-methoxycyclohexanone in CCl_4_ and CH_3_CN for the SO3LR models (−1 and −2-S/M/L) against the experimental component ratios;^62^ all models reproduce the predominance of the equatorial-*g* ^+^ band (Supporting Information). (D) Distribution (violin) of the mean-centered total-cluster energy error relative to PBE0+MBD for every potential on the 2-methoxycyclohexanone clusters in CCl_4_ and CH_3_CN (80 clusters each); numbers above each violin give MAE/SD (kcal mol^–1^; see the Supporting Information). Relative conformer energies against the B3LYP reference of Freitas et al.^62^ are given in the Supporting Information.

This behavior is mirrored in the infrared response: the predicted carbonyl band is dominated by the equatorial-*g* ^+^ stretch, with the minor axial bands and the red shift on increasing solvent polarity both recovered, reproducing the deconvoluted experimental spectrum of Freitas et al.^62^ (Figure 6B,C). The eq-*g* ^+^ band is the C = O stretch of the dominant conformer (Figure 6B, right); the predicted band lies 20–40 cm^–1^ above the experimental one, and the predicted CCl_4_ → CH_3_CN red shift is larger than observed. Benchmarking the explicit clusters directly against PBE0+MBD (Figure 6D) shows that the SO3LR-2 and MACE potentials reproduce the hybrid-DFT cluster energetics to within a few kcal mol^–1^, the larger models being the most accurate. The only marked exception is SO3LR-1 in CCl_4_, which correlates poorly with PBE0+MBD owing to a specific weakness for the chlorinated solvent rather than any failure of the microsolvation protocol; its conformer energetics and infrared response are unaffected. Per-model data for all three comparisons are collected in the Supporting Information.

The explicit treatment captures effects inaccessible to a uniform reaction field, but also highlights the limitations of a finite first solvation shell. Directional electrostatics and specific hydrogen bonds are represented atomistically, water donating to peptide carbonyls and amides, and the CH_3_CN dipole interacting with the ketone carbonyl, allowing MARS to recover the differential stabilization of exposed, high-dipole conformers that drives each equilibrium, rather than the conformer-independent solvation predicted by the Onsager model.^62^ However, this locality also leads to an underestimated stabilization of the metastable C7ax, α_*L*_ and α conformers of alanine dipeptide in water relative to the continuum reference, and to overstabilization of the most polar conformers relative to the implicit reference (notably eq-*g* ^–^ and eq-anti in the ketone), mirrored by the eq-*g* ^–^ intensity overshoot in CH_3_CN, and to an overestimation of the carbonyl solvatochromic shift. These effects arise because a single solvation shell overweights short-range interactions while neglecting long-range dielectric averaging and configurational entropy.

Consequently, the replica-to-replica variability (±0.4–2.5 kcal mol^–1^) is comparable to the smallest conformational energy differences and dominates the residual error, whereas gas-phase energies are essentially exact. This indicates that the residual error arises predominantly from the finite solvation shell and its sampling rather than from the gas-phase description of the potentials. MARS-solvation therefore captures the qualitative solvent-induced reordering and spectroscopic response of both systems, with quantitative agreement limited to a few kcal mol^–1^; closing the remaining gap is expected to require larger solvation shells, additional replicas, hybrid explicit/implicit treatments, and explicit free-energy sampling.

## 6 Host–Guest Chemistry: A Real Case Study of the MARS Protocol

We finally tested MARS on two larger, experimentally motivated systems that probe complementary aspects of conformational sampling. The first is the peptide pair Ace-Ala_5_-NMe and Ace-Ala_5_–LysH^+^, chosen to evaluate whether MARS can recover a charge-induced disorder–order transition. Short alanine peptides populate several competing backbone basins and are therefore standard models of shallow, intrinsically disordered peptides.^64–67^ In contrast, protonated lysine-capped alanine peptides are known to stabilize compact/helical motifs through charge-assisted self-solvation and backbone hydrogen bonding.^68,69^ The second system, l-proline included in β-cyclodextrin, tests a different regime: a large noncovalent host–guest complex for which conformational sampling must be connected to experimentally observable IR signatures.^63^

Both peptides were sampled using the default MARS-SO3LR-1 protocol. MARS identified 30 conformers for Ace-Ala_5_-NMe and 69 for Ace-Ala_5_–LysH^+^ within energy windows of approximately 10 kcal mol^–1^. Despite these similar energy ranges, the resulting ensembles differ markedly (Figure 7A,B). Ace-Ala_5_-NMe is distributed across α_R_, β, PPII, and other backbone regions, consistent with the conformational heterogeneity expected for a short alanine peptide. In contrast, Ace-Ala_5_–LysH^+^ shows a pronounced enrichment of α_R_ conformations, whose population increases from approximately 43 to 59%, together with a reduction in the populations of competing basins.

**Figure 7:**
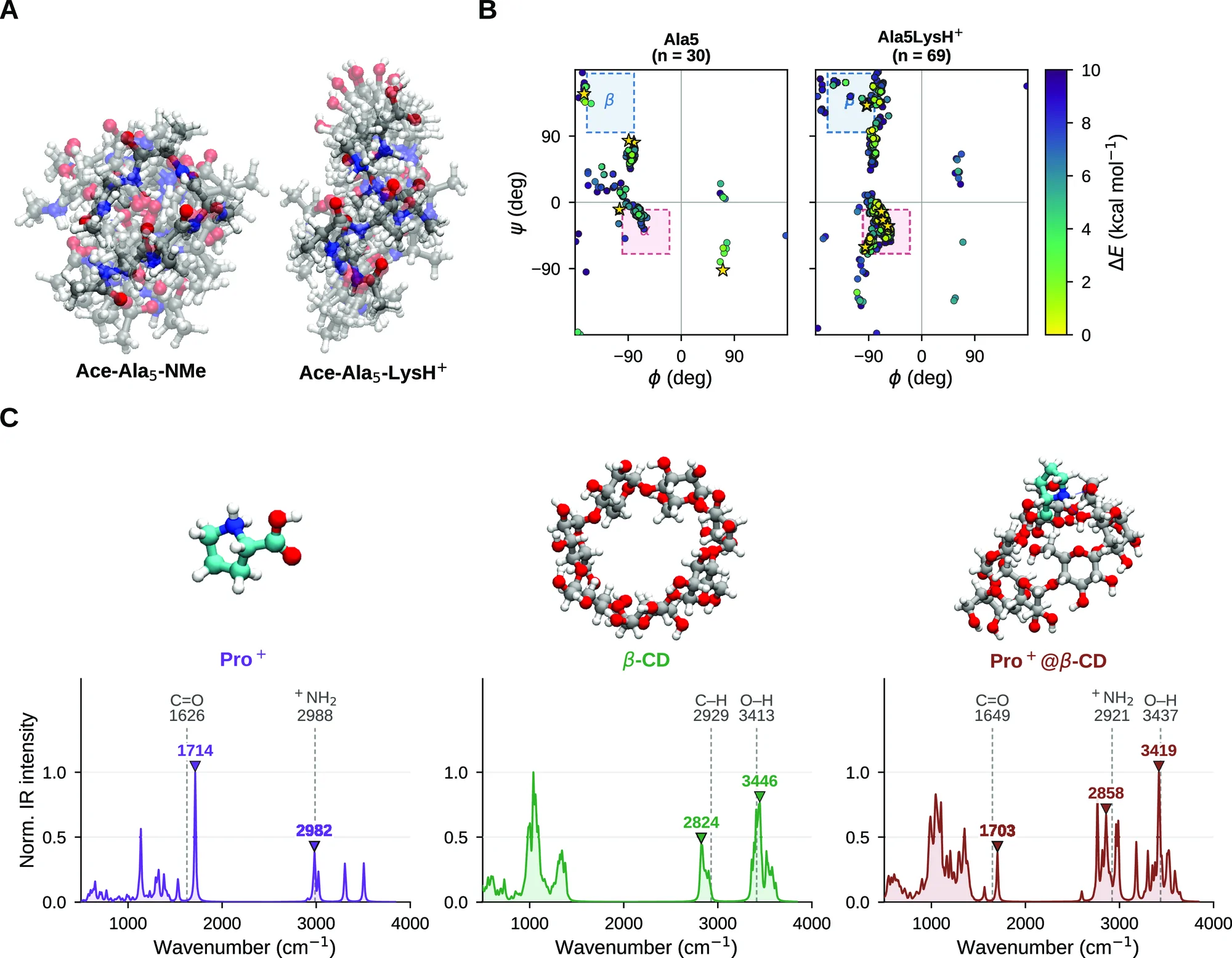
MARS-SO3LR-1 validation on larger molecular systems. (A) Superposition of the 10 lowest-energy conformers of Ace-Ala_5_-NMe, left, and Ace-Ala_5_–LysH^+^, right. The global minimum is opaque, and higher-energy conformers are translucent. (B) Ramachandran scatter over all alanine residues and conformers, colored by SO3LR relative energy, Δ*E* = *E* – *E*_min_; the gold star marks the global minimum. Dashed boxes indicate canonical α_R_ and β basins. (C) Simulated infrared spectra of cationic l-proline (Pro^+^), β-cyclodextrin (β-CD), and the Pro^+^@β-CD inclusion complex (left to right). Spectra were obtained from MARS conformational sampling followed by SO3LR-1 finite-difference (fd) Hessian frequency calculations, and are shown as 300 K Boltzmann-weighted averages over the sampled conformer ensembles (Lorentzian broadening, fwhm = 18 cm^–1^), with intensities normalized to the strongest band. Harmonic frequencies are scaled by a factor of 0.936. Filled triangles denote the predicted maxima of the diagnostic bands (^+^NH_2_, C = O, and N–H/O–H stretches), labeled with their scaled wavenumbers (cm^–1^); gray dashed lines mark the corresponding experimental FTIR band positions reported by Zaboub et al.^63^

Thus, without using structural templates, helical restraints, or system-specific sampling parameters, MARS reproduces the experimentally established tendency of the protonated lysine terminus to shift alanine-rich peptides toward helix-like conformations. The same qualitative behavior is found for the longer Ace-Ala_10_–LysH^+^ and Ace-Ala_15_–LysH^+^ peptides and when other MLFFs are used, indicating that the observed conformational reorganization is robust (SI Section S15).

We then considered the inclusion of proline in β-cyclodextrin. MARS-SO3LR-1 was used to sample free proline, isolated β-CD, and the corresponding inclusion complexes of neutral, anionic, and cationic proline. The sampled structures were subsequently optimized and used to calculate harmonic IR spectra, using all of the SO3LR variants (-1 and -2). We focus here on the cationic complex because it can be compared directly with the experimental FTIR assignment involving the protonated amine group.^63^

MARS-SO3LR-1 identified several possible inclusion geometries for each protonation state, with 23, 7, and 10 conformers obtained for the neutral, anionic, and cationic complexes, respectively. For the charged guests, however, one structure strongly dominates the predicted room-temperature ensemble, contributing approximately 93% of the population for the anionic complex and 88% for the cationic complex. The calculated IR spectrum of Pro^+^@β-CD is therefore governed primarily by a single preferred inclusion geometry.

The calculated complexation energies indicate favorable inclusion for all three protonation states, with stronger binding for the charged guests. This behavior is consistent with the formation of charge-assisted hydrogen bonds between proline and the hydroxyl groups at the β-CD rim. More importantly, the Boltzmann-averaged spectra reproduce the principal features of the experimental FTIR spectrum. The characteristic guest C = O, ^+^NH_2_, N–H, and O–H vibrations are retained, whereas the high-frequency region is dominated by the intense β-CD O–H envelope, which obscures weaker guest contributions (Figure 7C).

The calculations also reproduce the expected vibrational signatures of hydrogen-bond formation upon inclusion. In particular, guest donor vibrations undergo substantial red shifts; for Pro^+^, the carboxylic O–H stretching frequency shifts from 3744 to 2953 cm^–1^. The unscaled harmonic frequencies are systematically higher than the experimental values, as expected at this level of vibrational analysis. A uniform scaling factor of approximately 0.89 brings the calculated carbonyl stretching band into agreement with the experimental FTIR assignment. Calculations using other charge-aware potentials yield similar complexation strengths and spectral features, whereas models that do not explicitly account for molecular charge underestimate the binding of the ionic guests (SI Section S16).

Together, these applications show that MARS can connect conformational exploration of large, flexible systems with experimentally observed behavior. For alanine-rich peptides, the sampled ensembles reproduce the charge-induced shift from a heterogeneous conformational distribution toward helix-like structures. For Pro/β-CD, MARS identifies a dominant inclusion geometry and recovers the principal experimental IR signatures of host–guest complexation. These results illustrate the use of MARS as a practical workflow for generating experimentally meaningful conformational ensembles and interpreting structural and spectroscopic measurements.

## 7 Conclusions

We have presented MARS, a model-agnostic framework for automated conformational sampling, infrared spectroscopy, and explicit microsolvation with machine-learning force fields. The central result of this work is that modern MLFFs can already provide conformer energetics and vibrational observables at useful chemical accuracy while offering substantial acceleration relative to semiempirical quantum-mechanical methods, especially when combined with GPU-native implementations and batched sampling. This speedup enables more extensive conformational searches, larger ensembles, and a more routine treatment of systems that are difficult to explore with traditional electronic-structure-based workflows.

The applications considered here show that MARS can connect automated conformational exploration with experimentally relevant observables, including noncovalent binding motifs, solvent-dependent stability shifts, and IR spectral signatures. Quantitative predictions remain limited by the accuracy and chemical coverage of the selected potential, the completeness of finite temperature sampling, and the representation of explicit solvation. MARS does not remove these underlying physical limitations, but its model-agnostic design allows improved potentials and sampling strategies to be incorporated without redesigning the surrounding workflow.

MARS provides a practical route for replacing the semiempirical potential energy bottleneck with systematically improving MLFFs while integrating conformational sampling with vibrational spectroscopy and explicit microsolvation. As general-purpose MLFFs continue to improve in training coverage, energetic accuracy, and computational efficiency, the model-agnostic design of MARS allows these advances to be incorporated. This establishes MARS as a scalable framework for the next generation of automated ensemble-based molecular modeling.

## Supplementary Material



## Data Availability

The MARS source code, documentation, installation instructions, and example workflows are freely available at github.com/tcpunilu/mars and through the project webpage at tcpunilu.github.io/mars. The SO3LR-2 (version 2602) models will be made publicly available upon publication of the corresponding SO3LR-2 article. All results, input structures, either included directly or provided through references to the original sources, and scripts required to reproduce the results are available in the associated Zenodo repository at doi.org/10.5281/zenodo.22879791. DFT single-point calculations and geometry optimizations were performed using two density functional reference methods. ωB97M-V^70^ calculations were carried out with ORCA 6.0.0 ^71^ using the def2-TZVPD basis set. PBE0+MBD^72–74^ calculations were performed with FHI-aims^75^ (version 250320) using the *tight* numerical atom-centered orbital settings. Geometry optimizations used a maximum residual-force threshold of 5.0 × 10^–3^ eV/Å. All calculations used the molecular charge of the corresponding system and a spin multiplicity of one.

## Notes

Algorithmic and implementation details of the conformational-sampling, geometry optimization, infrared spectroscopy, microsolvation, noncovalent-interaction, and model-agnostic potential-interface modules; descriptions of the benchmark data sets; per-model relative conformer-energy, torsional, and dimer interaction-energy error tables; minimum-retention analyses against both DFT reference levels; numerical stability and reproducibility tests in single and double precision; noncovalent single-point and optimized-geometry statistics; harmonic IR accuracy and computational-scaling data for the NIST35 set; microsolvation model comparisons for alanine dipeptide and 2-methoxycyclohexanone; backbone conformational preferences of capped alanine peptides; and proline/β-cyclodextrin inclusion energies and infrared spectra (PDF)
