# Supplementary material for: MARS: Machine-Learned Force Field Framework for Automated Conformational Sampling, Vibrational Spectroscopy, and Microsolvation
Source: J Chem Theory Comput. 2026 Oct 21;22(21):11125–41. doi: 10.1021/acs.jctc.6c01588 (PMC13757767; doi:10.1021/acs.jctc.6c01588)
Supplement: Supplementary file 1 [file ct6c01588_si_001.pdf]

Supporting Information for

MARS: Machine-Learned Force Field Framework for Automated Conformational Sampling, Vibrational Spectroscopy, and Microsolvation

Sergio Suárez-Dou, Miguel Gallegos, Florian N. Brünig, Alexandre Tkatchenko*

Department of Physics and Materials Science, University of Luxembourg,
L-1511 Luxembourg City, Luxembourg
alexandre.tkatchenko@uni.lu

Contents

S1 Conformational Sampling **S6**
S1.1 Algorithm Overview S6
S1.2 Metadynamics Framework S6
S1.2.1 Gaussian Bias Potential S6
S1.2.2 RMSD Collective Variable S6
S1.2.3 Bias Force Computation S7
S1.2.4 Hill Deposition S7
S1.3 Automatic Parameter Configuration S7
S1.3.1 Flexibility Estimation S7
S1.3.2 Simulation Time Scaling S7
S1.3.3 Hill Height Scaling S7
S1.3.4 MTD Parameter Grid S8
S1.4 Four-Phase Workflow S8
S1.4.1 Phase 1: Multi-Step MTD Grid S8
S1.4.2 Phase 2: Conformer Refinement S8
S1.4.3 Phase 3: Multi-Temperature Rotamer MD S9
S1.4.4 Phase 4: Genetic Crossing S9
S1.5 Geometry Optimization S10
S1.5.1 Multi-Level Optimization Strategy S10
S1.6 Ensemble Pruning Pipeline S10
S1.6.1 Topology Validation S11
S1.6.2 Energy Window Filtering S11
S1.6.3 Rotational Constants Comparison S11
S1.6.4 RMSD Clustering S11
S1.6.5 Multi-Fragment Pruning S12
S1.7 Parallelization and Implementation S12
S1.7.1 JAX Transformation Stack S12
S1.7.2 Parallel MTD Execution S12
S1.7.3 Memory-Aware Batch Processing S13
S1.7.4 Neighbor List Management S13

*Corresponding author: alexandre.tkatchenko@uni.lu

S2 Geometry Optimization Module **S13**
S2.1 Optimization Algorithms . . . S13
S2.1.1 L-BFGS (Default) . . . S13
S2.1.2 FIRE (Fast Inertial Relaxation Engine) . . . S14
S2.1.3 Gradient Descent . . . S15
S2.1.4 Stationary Point Search (Damped Newton) . . . S15
S2.1.5 Redundant Internal Coordinates (optional) . . . S15
S2.2 Displacement Clipping . . . S16
S2.3 Safe Energy Function Wrapper . . . S16
S2.4 Parallel Batch Optimization . . . S16
S2.4.1 Batch Optimizer Construction . . . S16
S2.4.2 JIT-Compiled Optimization Loop . . . S16
S2.4.3 Neighbor List Handling in Batch Mode . . . S17
S2.5 Optimization Levels . . . S17
S2.6 Single-Point Energy Pre-Screening . . . S17

S3 Infrared Spectroscopy Tools **S17**
S3.1 Hessian-Based IR Calculation . . . S18
S3.1.1 Hessian Matrix Computation . . . S18
S3.1.2 Mass-Weighted Hessian . . . S18
S3.1.3 Normal Mode Computation . . . S18
S3.2 IR Intensity Calculation . . . S19
S3.2.1 Dipole Derivatives . . . S19
S3.2.2 Transformation to Normal Coordinates . . . S19
S3.2.3 IR Intensity . . . S19
S3.3 MD-Based IR Calculation . . . S19
S3.3.1 Dipole Autocorrelation Function . . . S19
S3.3.2 Wiener-Khinchin Theorem . . . S20
S3.3.3 Windowing Functions . . . S20
S3.3.4 MD Protocol . . . S20
S3.4 Normal Mode Classification . . . S20
S3.4.1 Wilson B-Matrix Formalism . . . S21
S3.4.2 Mode Projection . . . S21
S3.4.3 Classification Rules . . . S21
S3.4.4 Sub-Classification . . . S22
S3.5 Functional Group Identification . . . S22
S3.5.1 Molecular Graph Representation . . . S22
S3.5.2 Bond Order Assignment . . . S22
S3.5.3 Pattern Matching . . . S22
S3.5.4 Hierarchy Resolution . . . S23
S3.5.5 Mode-to-Functional-Group Mapping . . . S23
S3.6 Multi-Molecule Mode Attribution . . . S23
S3.6.1 Fragment Detection . . . S23
S3.6.2 Fragment Kinetic Energy Contribution . . . S24
S3.6.3 Mode Attribution . . . S24
S3.6.4 Hydrogen Bond Detection . . . S24
S3.7 Spectrum Output and Visualization . . . S24
S3.7.1 Static Plot (`--plot`) . . . S25
S3.7.2 Interactive Explorer (`mars viewer --ir`) . . . S25
S3.7.3 Additional Options . . . S26

S4 Microsolvation **S26**
S4.1 Solvent Placement Algorithm . . . S27
S4.1.1 Candidate Generation: Fibonacci Sphere Sampling . . . S27
S4.1.2 Van der Waals Overlap Rejection . . . S27
S4.1.3 Greedy Selection . . . S27
S4.2 Layer-by-Layer Shell Construction . . . S28
S4.2.1 Manual Mode . . . S28
S4.2.2 Automatic Mode . . . S28
S4.3 Solvent Library . . . S29
S4.3.1 Available Solvents . . . S29
S4.3.2 Solvent Properties . . . S29
S4.3.3 Custom Solvents . . . S30
S4.4 Shell Optimization . . . S30
S4.4.1 Frozen-Atom Optimization . . . S30
S4.4.2 Two-Stage Relaxation . . . S30
S4.5 Topology Repair . . . S30
S4.5.1 Reference Topology . . . S30
S4.5.2 Broken-Molecule Detection . . . S31
S4.5.3 Repair Loop . . . S31
S4.5.4 Defaults and Toggle . . . S31
S4.6 Multi-Conformer Solvation . . . S32
S4.6.1 Shell Transplantation . . . S32
S4.6.2 Kabsch Alignment . . . S32
S4.6.3 Batch Solvation . . . S32
S4.7 Barostat (Moving-Wall Droplet) Solvation . . . S33
S4.7.1 Spherical Wall Potentials . . . S33
S4.7.2 Barostat Compression . . . S33
S4.7.3 Solute Swap and Cleanup . . . S34

S5 Non-Covalent Interactions **S34**
S5.1 Multi-Fragment System Detection . . . S34
S5.1.1 Covalent Bond Detection . . . S34
S5.1.2 Connected Component Analysis . . . S34
S5.2 Confinement Potential . . . S35
S5.2.1 Confinement Geometry . . . S35
S5.2.2 Confinement Energy Functions . . . S35
S5.2.3 Total Confinement Energy . . . S35
S5.2.4 Automatic Boundary Determination . . . S36
S5.3 Inter-Fragment Distance Filtering . . . S36
S5.3.1 Cross-Fragment Distance Mask . . . S36
S5.3.2 Minimum Contact Distance . . . S36
S5.3.3 Adaptive Distance Threshold . . . S36
S5.3.4 Filtering Rule . . . S36
S5.4 Permutation-Invariant RMSD . . . S36
S5.4.1 Fragment Permutations . . . S37
S5.4.2 Implementation . . . S37
S5.5 NCI Workflow Integration . . . S37
S5.5.1 Activation . . . S37
S5.5.2 Modified Sampling . . . S37
S5.5.3 Workflow Checkpoints . . . S38
S5.6 Configuration Parameters . . . S38

S6 **Machine-Learned Potentials** S38
S6.1 Architecture Overview . . . S38
S6.1.1 Registry System . . . S38
S6.1.2 PotentialWrapper Base Class . . . S38
S6.2 Energy Function Requirements . . . S38
S6.2.1 Signature . . . S39
S6.2.2 JAX Differentiability . . . S39
S6.2.3 Force Computation . . . S39
S6.3 Neighbor List Interface . . . S39
S6.3.1 JAX-MD Integration . . . S39
S6.3.2 Neighbor State Management . . . S40
S6.4 Charge Support for IR Intensities . . . S40
S6.4.1 Charges Function . . . S40
S6.4.2 Dipole Moment . . . S40
S6.5 Built-in Potentials . . . S41
S6.5.1 SO3LR . . . S41
S6.5.2 MACE . . . S41
S6.5.3 GFN-xTB . . . S42
S6.6 Implementing Custom Potentials . . . S42
S6.6.1 Minimal Example . . . S42
S6.6.2 Example with Neighbor Lists . . . S42
S6.6.3 Example with Charges . . . S43
S6.6.4 Integration Checklist . . . S44

S7 **Graphical user interface** S45

S8 **Implementation validation** S47
S8.1 Part A: single-point energies and forces . . . S47
S8.2 Part B: molecular dynamics . . . S49

S9 **Conformational benchmark** S52

S10 **Reproducibility Test** S58
S10.1 Methods . . . S58
S10.1.1 System set . . . S58
S10.1.2 Sampling protocol . . . S58
S10.1.3 Test matrix . . . S58
S10.1.4 Reproducibility metrics . . . S58
S10.2 Results . . . S59
S10.2.1 Convergence rate . . . S59
S10.2.2 Reproducibility of the lowest-energy structure . . . S60
S10.2.3 Spread in the number of conformers found . . . S62
S10.3 Discussion . . . S63
S10.4 Recommendation . . . S63

S11 **Non-covalent benchmark** S64
S11.1 Dataset . . . S64
S11.2 Relative conformer energies (single point) . . . S65
S11.3 Optimized-geometry accuracy . . . S65

S12 Infrared benchmark **S67**
S12.1 Anharmonic spectra from molecular dynamics S67
S12.1.1 Molecular-dynamics protocol S67
S12.1.2 Results S68
S12.1.3 Computational cost S70

S13 Alanine dipeptide microsolvation: model comparison **S74**
S13.1 Geometric versus barostat microsolvation S77

S14 2-Methoxycyclohexanone: model comparison **S81**

S15 Backbone conformational preferences of capped alanine peptides **S83**

S16 Proline/β-cyclodextrin inclusion: model comparison **S85**

S17 Redundant Internal Coordinates **S86**

S18 Overlap between benchmark molecules and machine-learning training data **S89**
S18.1 Scope and definition of overlap S89
S18.2 Method S90
S18.2.1 Benchmark query index S90
S18.2.2 Canonical molecular key S90
S18.2.3 Streaming semi-join S91
S18.2.4 Per-dataset readers S92
S18.2.5 Validation S93
S18.3 Results S94
S18.4 Interpretation S95

S1 Conformational Sampling

S1.1 Algorithm Overview

The conformational sampling module performs automated generation of conformational ensembles for molecular systems. Given an input molecular structure, the algorithm systematically explores the potential energy surface to identify low-energy conformers within a specified energy window.

The workflow consists of four sequential phases:

1. **Multi-Step Metadynamics (MTD) Grid**: Parallel exploration using multiple bias parameter combinations
2. **Conformer Refinement**: Additional sampling from structurally diverse starting points
3. **Multi-Temperature Rotamer MD**: Unbiased molecular dynamics at elevated temperatures
4. **Genetic Crossing**: Z-matrix-based recombination of conformer dihedral angles

Input: Molecular geometry (XYZ format), charge, search mode (quick/normal/thorough)
Output: Ensemble of optimized conformers within the energy window, ranked by energy

S1.2 Metadynamics Framework

Metadynamics is employed to escape local energy minima by depositing repulsive Gaussian potentials (hills) at previously visited configurations. The collective variable (CV) used is the root-mean-square deviation (RMSD) from deposited hill centers.

S1.2.1 Gaussian Bias Potential

The total bias potential at configuration $\mathbf{R}$ is computed as:

$$V_{\text{bias}}(\mathbf{R}) = \sum_{i=1}^{N_{\text{hills}}} k_i \cdot \exp\left(-\alpha_i \cdot \text{RMSD}(\mathbf{R}, \mathbf{R}_i)^2\right) \tag{S1}$$

where:

- k_i is the hill height (in eV)
- α_i is the inverse Gaussian width (in Å^{-2})
- $\mathbf{R}_i$ is the atomic coordinates at hill i
- N_{hills} is the current number of deposited hills

S1.2.2 RMSD Collective Variable

The RMSD between two configurations is computed after optimal rigid-body alignment using the Kabsch algorithm [1]:

$$\text{RMSD}(\mathbf{R}, \mathbf{R}_{\text{ref}}) = \sqrt{\frac{1}{N} \sum_{i=1}^{N} |\mathbf{r}_i - \mathbf{r}_{i,\text{ref}}|^2} \tag{S2}$$

where N is the number of atoms and $\mathbf{r}_i$ are the Cartesian coordinates of atom i after alignment.

S1.2.3 Bias Force Computation

The bias force acting on atom j is obtained via automatic differentiation:

$$\mathbf{F}_{\text{bias},j} = -\frac{\partial V_{\text{bias}}}{\partial \mathbf{r}_j} \tag{S3}$$

During the MTD run, each frame is centered and Kabsch-aligned to the initial structure, and hills are stored in this common frame; the bias RMSD is evaluated in that frame without per-hill realignment, and the bias forces are obtained by JAX [2] automatic differentiation of Eq. (S1).

S1.2.4 Hill Deposition

Hills are deposited at regular intervals during the simulation:

- Deposition interval: $\tau_{\text{cvdump}} = 1000$ fs (default)
- A logistic damping function is applied to the most recently deposited hill to ensure smooth energy conservation

S1.3 Automatic Parameter Configuration

The algorithm automatically configures simulation parameters based on molecular properties, eliminating the need for manual parameter tuning.

S1.3.1 Flexibility Estimation

When a 3D structure is available (the default), molecular flexibility is estimated from a per-bond analysis of the covalent topology. Each internal (non-terminal) bond (i, j) contributes a factor

$$c_{ij} = b_{ij} \cdot r_{ij} \cdot o_{ij} \cdot h_{ij}, \qquad b_{ij} = \frac{2}{\sqrt{z_i\, z_j}}, \tag{S4}$$

where b_{ij} is a branching factor built from the heavy-atom coordination numbers z_i, z_j, and r_{ij}, o_{ij}, h_{ij} are reduction factors (each ≈ 0.5) that penalize ring membership, double/triple bond order, and sp^2 hybridization, respectively. The molecular flexibility is the quadratic mean of these per-bond factors,

$$f = \sqrt{\langle c_{ij}^2 \rangle}, \tag{S5}$$

with $f = 0$ for rigid (single-bond or fully constrained) systems and $f \to 1$ for long flexible chains.

When no structure is available, a heuristic fallback is used instead, $f = 0.7\sqrt{n_{\text{rot}}/n_{\text{heavy}}}\, c_{\text{conn}}$, where n_{rot} is the (approximate) number of rotatable bonds, n_{heavy} the number of heavy atoms, and c_{conn} a connectivity factor; this estimate is clamped to $[0.3, 1.0]$.

S1.3.2 Simulation Time Scaling

The MTD simulation time per grid step is scaled based on flexibility and system size:

$$t_{\text{MTD}} = 3.0 \times \exp\left(0.10 \times f \times \max(1, N_{\text{atoms}} - 8)\right) \tag{S6}$$

The result is clamped to the range $[5, 500]$ ps.

S1.3.3 Hill Height Scaling

The hill height k is scaled with system size:

$$k = k_{\text{start}} \times N_{\text{atoms}} \times s_{\text{MTD}} \tag{S7}$$

where k_{start} is the base hill height (in eV) and s_{MTD} is an optional global scaling factor (the `mtd_kscal` parameter, default 1.0).

S1.3.4 MTD Parameter Grid

Multiple (k, α) combinations are explored in parallel. The grid configuration depends on the search mode:

Table S1: MTD parameter grid configuration by search mode

Mode	$n_k \times n_\alpha$	Total Steps	Time Scaling	Conformer Seeds
quick	2×3	6	$0.5\times$	0
normal	3×4	12	$1.0\times$	3
thorough	4×6	24	$1.0\times$	6

The (k, α) values are distributed on an exponential grid that *descends* from a base (maximum) value, each successive step dividing by an increment factor:

$$k_j = \frac{k_{\text{base}}}{(\kappa_{\text{inc}})^j}, \quad j = 0, \ldots, n_k - 1, \quad k_{\text{base}} = k_{\text{start}} \times N_{\text{atoms}} \times s_{\text{MTD}} \tag{S8}$$

$$\alpha_m = \frac{\alpha_{\text{base}}}{(\gamma_{\text{inc}})^m}, \quad m = 0, \ldots, n_\alpha - 1 \tag{S9}$$

so that $j = 0$ ($m = 0$) corresponds to the strongest bias (largest hill height, narrowest Gaussian) and higher indices to progressively gentler, broader biases.

S1.4 Four-Phase Workflow

S1.4.1 Phase 1: Multi-Step MTD Grid

Before the production search, a short *trial MTD* (1 ps; $k = 1.0$ eV, $\alpha = 1.8$ Å^{-2}, hill dump every 20 fs, $\Delta t = 0.5$ fs) validates that the integration is stable for the given system. If the trajectory produces NaN coordinates or explodes (atoms more than 100 Å from the centroid), the timestep is halved and the trial is repeated as long as $\Delta t \geq 0.25$ fs (one retry from the default 0.5 fs); if the trial still fails, the search stops. The validated timestep is then used for the full search.

The first phase explores conformational space using parallel MTD simulations with different bias parameters.

Algorithm S1 Phase 1: Multi-Step MTD Grid

Require: Initial structure $\mathbf{R}_0$, parameter grid $\{(k_i, \alpha_i)\}_{i=1}^{N_{\text{grid}}}$, time t_{MTD}
Ensure: Conformer ensemble $\mathcal{E}$

1: $\mathcal{E} \leftarrow \emptyset$
2: **for all** $(k_i, \alpha_i) \in$ grid **do in parallel**
3: $\quad \text{traj}_i \leftarrow \text{RunMTD}(\mathbf{R}_0, k_i, \alpha_i, t_{\text{MTD}})$
4: $\quad \mathcal{E} \leftarrow \mathcal{E} \cup \text{ExtractSnapshots}(\text{traj}_i)$
5: **end for**
6: $\mathcal{E} \leftarrow \text{OptimizeMultilevel}(\mathcal{E})$
7: $\mathcal{E} \leftarrow \text{PruneEnsemble}(\mathcal{E})$
8: **return** $\mathcal{E}$

The Nosé-Hoover thermostat [3] is used for temperature control at $T = 300$ K (default).

S1.4.2 Phase 2: Conformer Refinement

Additional MTD runs are initiated from structurally diverse conformers identified in Phase 1.

Algorithm S2 Phase 2: Conformer Refinement

Require: Ensemble $\mathcal{E}$ from Phase 1, number of seeds N_{seeds}
Ensure: Refined ensemble $\mathcal{E}'$

$\mathcal{S} \leftarrow \text{SelectMostDiverse}(\mathcal{E}, N_{\text{seeds}})$ ▷ RMSD-based selection
for all $\mathbf{R}_s \in \mathcal{S}$ **do in parallel**
  $\text{traj}_s \leftarrow \text{RunMTD}(\mathbf{R}_s, 0.75\, k_{\text{med}}, \alpha_{\text{base}}, 0.5 \times t_{\text{MTD}})$
  $\mathcal{E} \leftarrow \mathcal{E} \cup \text{ExtractSnapshots}(\text{traj}_s)$
end for
$\mathcal{E}' \leftarrow \text{OptimizeMultilevel}(\mathcal{E})$
$\mathcal{E}' \leftarrow \text{PruneEnsemble}(\mathcal{E}')$
return $\mathcal{E}'$

The diverse subset is selected using a greedy algorithm that maximizes the minimum pairwise RMSD. The refinement bias uses the median hill height scaled by 0.75 and the base (narrowest) Gaussian width α_{base}.

S1.4.3 Phase 3: Multi-Temperature Rotamer MD

Unbiased molecular dynamics at multiple temperatures captures rotamer transitions that may be missed by biased sampling.

Algorithm S3 Phase 3: Multi-Temperature Rotamer MD

Require: Ensemble $\mathcal{E}$ from Phase 2, base temperature T_0, number of temperatures N_T
Ensure: Extended ensemble $\mathcal{E}'$

$\mathcal{T} \leftarrow \{T_0, T_0 + 100\text{K}, T_0 + 200\text{K}, \ldots\}$ ▷ N_T temperatures
$\mathcal{S} \leftarrow \text{SelectLowestEnergy}(\mathcal{E}, N_{\text{starts}})$
for all $(\mathbf{R}_s, T) \in \mathcal{S} \times \mathcal{T}$ **do in parallel**
  $\text{traj}_{s,T} \leftarrow \text{RunNVT}(\mathbf{R}_s, T, t_{\text{MD}})$
  $\mathcal{E} \leftarrow \mathcal{E} \cup \text{ExtractSnapshots}(\text{traj}_{s,T})$
end for
$\mathcal{E}' \leftarrow \text{OptimizeMultilevel}(\mathcal{E})$
$\mathcal{E}' \leftarrow \text{PruneEnsemble}(\mathcal{E}')$
return $\mathcal{E}'$

S1.4.4 Phase 4: Genetic Crossing

Genetic crossing generates new conformers by recombining the internal coordinates of parent conformers in Z-matrix (internal coordinate) representation.

The Z-matrix representation defines each atom by:

- Bond length to a reference atom
- Bond angle with two reference atoms
- Dihedral angle with three reference atoms

The crossing operation generates a child conformer:

$$\mathbf{q}_{\text{child}} = \mathbf{q}_{\text{ref}} + (\mathbf{q}_{\text{low}} - \mathbf{q}_{\text{high}}) \tag{S10}$$

where $\mathbf{q}$ comprises all Z-matrix coordinates (bond lengths, angles, and dihedrals; dihedral differences are wrapped to $[-\pi, \pi]$), $\mathbf{q}_{\text{ref}}$ is the lowest-energy conformer, and $\mathbf{q}_{\text{low}}, \mathbf{q}_{\text{high}}$ are Boltzmann-selected parents.

Algorithm S4 Phase 4: Genetic Crossing

Require: Ensemble $\mathcal{E}$ from Phase 3, number of children N_{children}
Ensure: Final ensemble $\mathcal{E}'$

$\mathbf{R}_{\text{ref}} \leftarrow \text{LowestEnergy}(\mathcal{E})$
$\mathbf{q}_{\text{ref}} \leftarrow \text{CartesianToZMatrix}(\mathbf{R}_{\text{ref}})$
for $i = 1$ **to** N_{children} **do**
  $\mathbf{R}_{\text{low}}, \mathbf{R}_{\text{high}} \leftarrow \text{BoltzmannSelect}(\mathcal{E})$
  $\mathbf{q}_{\text{low}} \leftarrow \text{CartesianToZMatrix}(\mathbf{R}_{\text{low}})$
  $\mathbf{q}_{\text{high}} \leftarrow \text{CartesianToZMatrix}(\mathbf{R}_{\text{high}})$
  $\mathbf{q}_{\text{child}} \leftarrow \mathbf{q}_{\text{ref}} + (\mathbf{q}_{\text{low}} - \mathbf{q}_{\text{high}})$
  $\mathbf{R}_{\text{child}} \leftarrow \text{ZMatrixToCartesian}(\mathbf{q}_{\text{child}})$ ▷ NeRF algorithm
  if $\neg\text{HasClash}(\mathbf{R}_{\text{child}})$ **then**
    $\mathcal{E} \leftarrow \mathcal{E} \cup \{\mathbf{R}_{\text{child}}\}$
  end if
end for
$\mathcal{E}' \leftarrow \text{OptimizeMultilevel}(\mathcal{E})$
$\mathcal{E}' \leftarrow \text{PruneEnsemble}(\mathcal{E}')$
return $\mathcal{E}'$

Cartesian coordinates are reconstructed from internal coordinates using the Natural Extension Reference Frame (NeRF) algorithm.

Clash detection uses coordination numbers with a threshold $\Delta CN_{\text{max}} = 0.3$ per atom and a hard distance ratio threshold of 0.7.

S1.5 Geometry Optimization

Structure optimization is performed using gradient-based methods with multi-level refinement. The optimizer module (Section S2) provides multiple optimization algorithms (L-BFGS, FIRE, Gradient Descent, Stationary Point search) with displacement clipping and parallel batch execution.

S1.5.1 Multi-Level Optimization Strategy

Optimization proceeds through three cycles with progressively tighter convergence criteria:

Table S2: Multi-level optimization parameters

Cycle	f_{max}	Energy Window	Max Iterations	Max Step
1	$50 \times f_{\text{target}}$	$2.0 \times E_{\text{win}}$	10	1.0 Å
2	$10 \times f_{\text{target}}$	$1.5 \times E_{\text{win}}$	100	0.5 Å
3	$1 \times f_{\text{target}}$	$1.0 \times E_{\text{win}}$	1000	0.2 Å

Default convergence threshold: $f_{\text{target}} = 0.01$ eV/Å. Default energy window: $E_{\text{win}} = 10$ kcal/mol (0.433 eV).

Between cycles, structures outside the energy window are discarded, reducing computational cost for subsequent cycles.

S1.6 Ensemble Pruning Pipeline

After each optimization cycle, the ensemble is filtered through a multi-stage pruning pipeline.

S1.6.1 Topology Validation

Structures with broken, excessively stretched, or newly formed bonds are discarded by comparing the current geometry against the reference bond list (from standard covalent radii $L_0 = r_i^{\text{cov}} + r_j^{\text{cov}}$). A reference bond (i, j) is considered intact only if

$$d_{ij} \leq \min\bigl(1.6\, L_0,\ L_0 + 0.60\ \text{Å}\bigr), \tag{S11}$$

and the structure is additionally rejected if any non-bonded pair comes within covalent range, $d_{ij} < 1.2\,(r_i^{\text{cov}} + r_j^{\text{cov}})$ (a newly formed bond). This catches both dissociation/over-stretching and unintended bond formation during sampling.

S1.6.2 Energy Window Filtering

Structures above the energy threshold relative to the global minimum are removed:

$$\text{Keep if: } E - E_{\text{min}} \leq E_{\text{win}} \tag{S12}$$

Default: $E_{\text{win}} = 10$ kcal/mol (0.433 eV).

Coarse-cycle safeguard. In the early (coarse) optimization cycles the geometries are only loosely converged—small iteration budgets and large step caps (Table S2)—so their energies are unreliable, and an aggressive window can collapse the entire batch onto the single lowest structure, which may then fragment in the next cycle and abort the search. To guard against this, whenever energy-window filtering would reduce a multi-structure ensemble to a single conformer (or none) in a *non-final* optimization cycle, the filter is skipped for that cycle—with a warning—and all structures are carried forward to the next cycle, where the window is re-applied to the better-converged geometries. The topology, rotational-constant, and RMSD filters still run in that cycle, so only the energy criterion is deferred; the final cycle always applies the window normally.

S1.6.3 Rotational Constants Comparison

Structures with similar rotational constants (principal moments of inertia) are considered duplicates:

$$\text{Duplicate if: } \left|\frac{A - A'}{A}\right| < \epsilon_{\text{rot}} \quad \text{and} \quad \left|\frac{B - B'}{B}\right| < \epsilon_{\text{rot}} \quad \text{and} \quad \left|\frac{C - C'}{C}\right| < \epsilon_{\text{rot}} \tag{S13}$$

Default threshold: $\epsilon_{\text{rot}} = 0.02$ (2% relative difference).

S1.6.4 RMSD Clustering

Near-duplicate structures are removed using greedy RMSD-based clustering:

Algorithm S5 RMSD-Based Pruning

Require: Ensemble $\mathcal{E}$ sorted by energy, RMSD threshold ϵ_{RMSD}
Ensure: Pruned ensemble $\mathcal{E}'$

```
1: E' ← {E[0]}                              ▷ Keep lowest-energy structure
2: for i = 1 to |E| − 1 do
3:     d_min ← min_{R'∈E'} RMSD(E[i], R')
4:     if d_min > ε_RMSD then
5:         E' ← E' ∪ {E[i]}
6:     end if
7: end for
8: return E'
```

Default threshold: $\epsilon_{\text{RMSD}} = 0.25$ Å.

Both the RMSD and rotational-constant filters additionally accept a *reference* ensemble: conformers accumulated in previous phases are passed in so that new structures matching the running ensemble are discarded across phases, not only within the current batch.

S1.6.5 Multi-Fragment Pruning

For non-covalent (multi-fragment) systems, two extra stages are applied. First, an inter-fragment *dissociation* filter discards structures whose minimum inter-fragment contact distance exceeds a threshold (Section S5); the threshold is relaxed in early optimization cycles (factors 2.0, 1.5, 1.0 for cycles 1–3). Second, when equivalent fragments are present, structural similarity is assessed with a permutation-invariant RMSD that minimizes over all equivalent-fragment relabelings (Section S5.4), preventing artificial inflation of ensemble diversity.

S1.7 Parallelization and Implementation

The conformational sampling workflow is implemented using JAX [2] for automatic differentiation, JIT compilation, and vectorized parallel execution.

S1.7.1 JAX Transformation Stack

Three key JAX transformations enable efficient parallel execution:

1. **JIT Compilation** (`jax.jit`): All energy, force, and optimization functions are compiled to XLA (Accelerated Linear Algebra) for GPU/TPU execution. Compilation occurs once on first call; subsequent calls reuse the compiled kernel.

2. **Automatic Differentiation** (`jax.grad`): Forces are computed as exact gradients of the energy function via reverse-mode automatic differentiation:

$$\mathbf{F} = -\nabla_{\mathbf{R}} E(\mathbf{R}) = -\texttt{jax.grad}(E)(\mathbf{R}) \tag{S14}$$

3. **Vectorization** (`jax.vmap`): Single-structure functions are automatically lifted to batch operations over the leading array dimension:

$$\texttt{vmap}(f) : \mathbb{R}^{N\times 3} \to \mathbb{R} \quad \Rightarrow \quad \mathbb{R}^{B\times N\times 3} \to \mathbb{R}^{B} \tag{S15}$$

   where B is the batch size.

S1.7.2 Parallel MTD Execution

The MTD parameter grid is evaluated using vectorized parallel execution:

Algorithm S6 Parallel MTD Grid Execution

Require: Initial positions $\mathbf{R}_0$, parameter grid $\{(k_i, \alpha_i)\}_{i=1}^{N_{\text{grid}}}$
Ensure: Trajectories $\{\text{traj}_i\}_{i=1}^{N_{\text{grid}}}$
1: $\mathbf{R}_{\text{batch}} \leftarrow \text{Stack}([\mathbf{R}_0] \times N_{\text{grid}})$ ▷ $(N_{\text{grid}}, N, 3)$
2: $\text{MTDState}_{\text{batch}} \leftarrow \text{vmap}(\text{InitMTD})(\mathbf{k}, \boldsymbol{\alpha})$
3: **for** $t = 1$ **to** N_{steps} **do**
4: $\quad \mathbf{R}_{\text{batch}}, \text{MTDState}_{\text{batch}} \leftarrow \text{vmap}(\text{MTDStep})(\mathbf{R}_{\text{batch}}, \text{MTDState}_{\text{batch}})$
5: **end for**
6: **return** $\text{ExtractSnapshots}(\mathbf{R}_{\text{batch}})$

Each replica maintains independent state (positions, velocities, deposited hills, neighbor lists) while sharing the same compiled computation kernel.

S1.7.3 Memory-Aware Batch Processing

For large ensembles, batch sizes are automatically adjusted based on available GPU memory:

Algorithm S7 Memory-Aware Batch Optimization

Require: Positions batch $\mathbf{R}_{\text{batch}}$ of size N
Ensure: Optimized positions $\mathbf{R}_{\text{opt}}$

try:
  $\mathbf{R}_{\text{opt}} \leftarrow \text{vmap(Optimize)}(\mathbf{R}_{\text{batch}})$
  BlockUntilReady($\mathbf{R}_{\text{opt}}$) ▷ Force synchronization
catch OutOfMemoryError:
  ClearCaches()
  mid $\leftarrow N/2$
  $\mathbf{R}^{(1)}_{\text{opt}} \leftarrow \text{BatchOptimize}(\mathbf{R}_{\text{batch}}[:\text{mid}])$ ▷ Recursive
  $\mathbf{R}^{(2)}_{\text{opt}} \leftarrow \text{BatchOptimize}(\mathbf{R}_{\text{batch}}[\text{mid}:])$
  $\mathbf{R}_{\text{opt}} \leftarrow \text{Concatenate}(\mathbf{R}^{(1)}_{\text{opt}}, \mathbf{R}^{(2)}_{\text{opt}})$
return $\mathbf{R}_{\text{opt}}$

This binary splitting strategy ensures robust execution across varying GPU memory constraints without manual batch size tuning.

S1.7.4 Neighbor List Management

For potentials using neighbor lists (e.g., SO3LR, MACE), each structure in a batch maintains its own neighbor list state. Neighbor lists are updated within the JIT-compiled optimization loop:

1. **Allocation**: Neighbor lists are pre-allocated with capacity for the maximum expected neighbors per atom.
2. **Update**: After each geometry step, neighbor lists are updated based on new positions.
3. **Overflow handling**: During MD/MTD, neighbor-list overflow is checked after each block of saved steps and the list is reallocated from the current positions.

S2 Geometry Optimization Module

The geometry optimization module provides GPU-accelerated structure minimization with multiple optimization algorithms, displacement clipping for numerical stability, and parallel batch execution via JAX vectorization.

S2.1 Optimization Algorithms

Four optimization methods are implemented, each suited to different optimization scenarios.

S2.1.1 L-BFGS (Default)

The Limited-memory Broyden-Fletcher-Goldfarb-Shanno algorithm [4] is a quasi-Newton method that approximates the inverse Hessian using a limited history of position and gradient differences.

Algorithm. At iteration k, the search direction is computed as:

$$\mathbf{p}_k = -\mathbf{H}_k \mathbf{g}_k \tag{S16}$$

where $\mathbf{g}_k = \nabla E(\mathbf{x}_k)$ is the gradient and $\mathbf{H}_k$ is the approximate inverse Hessian constructed from the most recent m pairs:

$$\mathbf{s}_i = \mathbf{x}_{i+1} - \mathbf{x}_i \tag{S17}$$

$$\mathbf{y}_i = \mathbf{g}_{i+1} - \mathbf{g}_i \tag{S18}$$

The inverse Hessian-vector product $\mathbf{H}_k \mathbf{g}_k$ is computed via the two-loop recursion without explicitly forming $\mathbf{H}_k$.

Line search. A backtracking line search (instead of the zoom line search that is the default in the underlying `jaxopt` L-BFGS implementation) determines the step length α_k:

$$\mathbf{x}_{k+1} = \mathbf{x}_k + \alpha_k \mathbf{p}_k \tag{S19}$$

Parameters:

- History size: $m = 10$ (default)
- Step increase factor: 1.2
- Convergence: $\|\nabla E\|_2 < f_{\max}$ (norm over all $3N$ components)

S2.1.2 FIRE (Fast Inertial Relaxation Engine)

FIRE [5] combines molecular dynamics with adaptive velocity modification to achieve fast convergence for systems far from equilibrium.

Algorithm. The velocity is modified at each step:

$$\mathbf{v} \leftarrow (1-\alpha)\mathbf{v} + \alpha|\mathbf{v}|\hat{\mathbf{F}} \tag{S20}$$

where $\hat{\mathbf{F}} = \mathbf{F}/|\mathbf{F}|$ is the normalized force direction and $\alpha \in [0,1]$ controls the mixing.

Adaptive timestep. The timestep Δt and mixing parameter α are adjusted based on the power $P = \mathbf{F} \cdot \mathbf{v}$:

$$\text{if } P > 0 \text{ and } n_{\text{pos}} > N_{\min} : \quad \begin{cases} \Delta t \leftarrow \min(\Delta t \cdot f_{\text{inc}}, \Delta t_{\max}) \\ \alpha \leftarrow \alpha \cdot f_\alpha \end{cases} \tag{S21}$$

$$\text{else} : \quad \begin{cases} \mathbf{v} \leftarrow 0 \\ \Delta t \leftarrow \Delta t \cdot f_{\text{dec}} \\ \alpha \leftarrow \alpha_{\text{start}} \end{cases} \tag{S22}$$

Parameters:

- Initial timestep: $\Delta t_{\text{start}} = 0.05$ (JAX-MD time units, unit masses)
- Maximum timestep: $\Delta t_{\max} = 0.1$ (JAX-MD time units)
- Increase factor: $f_{\text{inc}} = 1.1$

- Decrease factor: $f_{\text{dec}} = 0.5$
- Mixing decay: $f_\alpha = 0.99$
- Initial mixing: $\alpha_{\text{start}} = 0.1$
- Minimum positive steps: $N_{\text{min}} = 2$

S2.1.3 Gradient Descent

Standard gradient descent with fixed step size:

$$\mathbf{x}_{k+1} = \mathbf{x}_k - \eta \nabla E(\mathbf{x}_k) \tag{S23}$$

where η is the learning rate (step size). Used primarily for testing and comparison.

S2.1.4 Stationary Point Search (Damped Newton)

For locating transition states and saddle points, a damped Newton method with trust-radius control is used:

Newton step. The displacement is computed by solving:

$$(\mathbf{H} + \mu \mathbf{I})\boldsymbol{\delta} = -\mathbf{g} \tag{S24}$$

where $\mathbf{H}$ is the Hessian matrix, $\mathbf{g}$ is the gradient, and μ is a damping parameter that ensures the matrix is positive definite.

Trust radius. The step is scaled to stay within a trust radius r_{trust}:

$$\boldsymbol{\delta} \leftarrow \boldsymbol{\delta} \cdot \min\left(1, \frac{r_{\text{trust}}}{|\boldsymbol{\delta}|}\right) \tag{S25}$$

Adaptive damping. The damping parameter μ is adjusted based on step acceptance:

$$\mu \leftarrow \begin{cases} 10\mu & \text{if step severely truncated (scale} < 0.2) \\ 0.7\mu & \text{if step nearly accepted (scale} > 0.9) \\ \mu & \text{otherwise} \end{cases} \tag{S26}$$

with μ clamped to $[10^{-12}, 10^6]$.

Convergence. The search terminates when:

$$\max_i |\mathbf{F}_i| < f_{\max} \quad \text{and} \quad |\boldsymbol{\delta}| < \epsilon_{\text{step}} \tag{S27}$$

S2.1.5 Redundant Internal Coordinates (optional)

The four methods above all operate in Cartesian coordinates, which is the default throughout MARS. As an opt-in alternative, `mars optimize --coords internal` runs a rational-function-optimization stepper in redundant internal coordinates with a chemically informed initial Hessian [6]. It converges in far fewer iterations than Cartesian L-BFGS, but each iteration is correspondingly more expensive and it requires double precision, so it brings no net gain to the MARS workflow and is provided as an optimizer for specific applications rather than as a default. It is exposed on `mars optimize` only, leaving the conformational-search workflow unaffected; the comparison is given in Section S17.

S2.2 Displacement Clipping

To prevent numerical instabilities from large gradient steps (especially on the first iteration or near clashes), atomic displacements are clipped to a maximum value.

Clipping operation. After computing the raw step $\boldsymbol{\delta} = \mathbf{x}_{\text{new}} - \mathbf{x}_{\text{old}}$:

$$d_{\max} = \max_i \|\boldsymbol{\delta}_i\| \tag{S28}$$

$$\text{if } d_{\max} > \delta_{\text{clip}}: \quad \boldsymbol{\delta} \leftarrow \boldsymbol{\delta} \cdot \frac{\delta_{\text{clip}}}{d_{\max}} \tag{S29}$$

Default clip threshold: $\delta_{\text{clip}} = 0.2$ Å.

State reset. When clipping occurs in L-BFGS, the optimizer state (inverse Hessian approximation) is reset to maintain consistency between the stored history and the actual displacement taken:

$$\text{if clipped}: \quad \mathbf{H}_k \leftarrow \mathbf{H}_0 \quad \text{(reset to initial)} \tag{S30}$$

This prevents the quasi-Newton approximation from becoming inconsistent with the clipped trajectory.

S2.3 Safe Energy Function Wrapper

To ensure robust optimization, energy functions are wrapped to handle numerical edge cases:

$$E_{\text{safe}}(\mathbf{x}) = \begin{cases} E(\mathbf{x}) & \text{if } E(\mathbf{x}) \in \mathbb{R} \text{ (finite)} \\ E_{\text{large}} & \text{if } E(\mathbf{x}) = \text{NaN or } \pm\infty \end{cases} \tag{S31}$$

Default: $E_{\text{large}} = 10^4$ eV. This prevents the optimizer's internal convergence loop from spinning indefinitely on non-finite energies.

S2.4 Parallel Batch Optimization

Multiple structures are optimized simultaneously using JAX's `vmap` transformation.

S2.4.1 Batch Optimizer Construction

A single-structure optimizer is lifted to a batch optimizer:

Algorithm S8 Batch Optimizer via vmap

Require: Energy function E, convergence threshold $f_{\max}$, max iterations N_{iter}
Ensure: Batch optimizer function

1: OptimizeOne ← BuildSingleOptimizer($E, f_{\max}, N_{\text{iter}}$)
2: OptimizeBatch ← `jax.jit`(`jax.vmap`(OptimizeOne))
3: **return** OptimizeBatch

S2.4.2 JIT-Compiled Optimization Loop

The optimization loop is fully JIT-compiled using `jax.lax.while_loop`:

Algorithm S9 JIT-Compiled Single Structure Optimization

Require: Initial positions $\mathbf{x}_0$, energy function E, threshold $f_{\max}$, max iterations N
Ensure: Optimized positions $\mathbf{x}^*$, energy E^*, convergence flag
1: $\text{state}_0 \leftarrow \text{InitOptimizer}(\mathbf{x}_0)$
2: **define** $\text{cond}(\text{state}, k) \leftarrow (k < N) \wedge (\text{state.error} \geq f_{\max})$
3: **define** $\text{body}(\text{state}, k) \leftarrow (\text{OptimizerStep}(\text{state}), k + 1)$
4: $\text{state}^*, k^* \leftarrow$ `jax.lax.while_loop`$(\text{cond}, \text{body}, (\text{state}_0, 0))$
5: **return** $\text{state}^*.\mathbf{x}$, $E(\text{state}^*.\mathbf{x})$, $\text{state}^*.\text{error} < f_{\max}$

The entire loop (including convergence checks) is compiled into a single GPU kernel, eliminating Python overhead.

S2.4.3 Neighbor List Handling in Batch Mode

For potentials with neighbor lists, neighbor-list updates are incorporated into the JIT-compiled loop, and each structure in the batch maintains independent neighbor state:

$$(\mathbf{x}_{k+1}^{(i)}, \text{nbr}_{k+1}^{(i)}) = \text{Step}(\mathbf{x}_k^{(i)}, \text{nbr}_k^{(i)}) \quad \forall i \in [1, B] \tag{S32}$$

S2.5 Optimization Levels

Predefined optimization levels map to force convergence thresholds:

Table S3: Optimization level definitions

Level	$f_{\max}$ (eV/Å)	Description
−3	0.05	Very loose
−2	0.03	Loose
−1	0.02	Normal loose
0	0.01	Default
+1	0.005	Tight
+2	0.002	Very tight

S2.6 Single-Point Energy Pre-Screening

Before full geometry optimization, structures can be filtered using single-point energies to eliminate clearly high-energy configurations:

$$\text{Keep structure } i \text{ if: } E_{\text{SP}}^{(i)} \leq E_{\text{ref}} + E_{\text{buffer}} \tag{S33}$$

Default buffer: $E_{\text{buffer}} = 4 \times E_{\text{win}}$, where E_{win} is the target energy window.

If this pre-screen would reject *every* structure—for example when a backend places the single-point reference energy far from the sampled geometries—it is skipped entirely and all structures are passed to optimization, with a warning, rather than returning an empty ensemble.

Single-point energies are computed in parallel using `vmap`, with the same OOM-handling strategy as batch optimization.

S3 Infrared Spectroscopy Tools

The infrared spectroscopy module computes vibrational spectra, classifies normal modes, and identifies functional group contributions. Two complementary approaches are implemented: Hessian-based (harmonic approximation) and MD-based (dynamic, anharmonic).

S3.1 Hessian-Based IR Calculation

The harmonic approximation computes vibrational frequencies and IR intensities from the mass-weighted Hessian matrix.

S3.1.1 Hessian Matrix Computation

The Hessian matrix $\mathbf{H}$ contains second derivatives of the potential energy with respect to atomic displacements:

$$H_{i\alpha,j\beta} = \frac{\partial^2 E}{\partial r_{i\alpha} \partial r_{j\beta}} \tag{S34}$$

where i, j index atoms and $\alpha, \beta \in \{x, y, z\}$ index Cartesian components.

Analytical Hessian (default). Computed via JAX automatic differentiation:

$$\mathbf{H} = \texttt{jax.hessian}(E)(\mathbf{R}) \tag{S35}$$

This is exact and fully JIT-compiled.

Finite-difference Hessian (fallback). For potentials incompatible with JAX autodiff (e.g., those using `pure_callback`), the Hessian is built from central differences of the analytical *gradient* (forces), one displaced column per Cartesian coordinate:

$$\mathbf{H}_{:,j} \approx \frac{\nabla E(\mathbf{R} + \delta \mathbf{e}_j) - \nabla E(\mathbf{R} - \delta \mathbf{e}_j)}{2\,\delta}, \tag{S36}$$

followed by symmetrization $\mathbf{H} \leftarrow \frac{1}{2}(\mathbf{H} + \mathbf{H}^\top)$. This requires only $2 \times 3N$ gradient evaluations rather than an $\mathcal{O}(N^2)$ energy stencil. Default displacement: $\delta = 0.01$ Å (`--fd-displacement`).

S3.1.2 Mass-Weighted Hessian

The Hessian is mass-weighted to transform to mass-weighted coordinates:

$$\tilde{H}_{i\alpha,j\beta} = \frac{H_{i\alpha,j\beta}}{\sqrt{m_i m_j}} \tag{S37}$$

where m_i is the atomic mass of atom i in atomic mass units (amu).

S3.1.3 Normal Mode Computation

Diagonalization of the mass-weighted Hessian yields eigenvalues λ_k and eigenvectors $\mathbf{L}_k$:

$$\tilde{\mathbf{H}}\mathbf{L}_k = \lambda_k \mathbf{L}_k \tag{S38}$$

The vibrational frequencies are:

$$\nu_k = \frac{1}{2\pi} \sqrt{\frac{\lambda_k \cdot E_{\text{conv}}}{m_{\text{conv}}}} \tag{S39}$$

where E_{conv} and m_{conv} are unit conversion factors (eV to J, amu to kg).

In wavenumbers (cm^{-1}):

$$\tilde{\nu}_k = \frac{\nu_k}{c \times 100} \tag{S40}$$

where c is the speed of light in m/s.

Filtering. Modes with $\tilde{\nu}_k < 50$ cm^{-1} are classified as translations or rotations and excluded from the vibrational spectrum.

S3.2 IR Intensity Calculation

IR intensity is proportional to the square of the dipole moment derivative with respect to the normal coordinate.

S3.2.1 Dipole Derivatives

The dipole moment derivative with respect to Cartesian coordinates:

$$\frac{\partial \boldsymbol{\mu}}{\partial r_{i\alpha}} = \sum_j \frac{\partial q_j}{\partial r_{i\alpha}} \mathbf{r}_j + q_i \mathbf{e}_\alpha \tag{S41}$$

where q_j are atomic partial charges.

Analytical computation. For differentiable potentials providing charges (SO3LR):

$$\frac{\partial \boldsymbol{\mu}}{\partial \mathbf{R}} = \texttt{jax.jacfwd}(\boldsymbol{\mu})(\mathbf{R}) \tag{S42}$$

Finite-difference computation. When analytical derivatives are unavailable:

$$\frac{\partial \mu_\gamma}{\partial r_{i\alpha}} \approx \frac{\mu_\gamma(\mathbf{R} + \delta \mathbf{e}_{i\alpha}) - \mu_\gamma(\mathbf{R} - \delta \mathbf{e}_{i\alpha})}{2\delta} \tag{S43}$$

S3.2.2 Transformation to Normal Coordinates

The dipole derivative is transformed from Cartesian to normal coordinates:

$$\frac{\partial \boldsymbol{\mu}}{\partial Q_k} = \sum_{i,\alpha} \frac{\partial \boldsymbol{\mu}}{\partial r_{i\alpha}} \cdot \frac{L_{k,i\alpha}}{\sqrt{m_i}} \tag{S44}$$

where $L_{k,i\alpha}$ is the k-th eigenvector component and the factor $1/\sqrt{m_i}$ converts from mass-weighted to normal coordinates.

S3.2.3 IR Intensity

The IR intensity for mode k:

$$I_k = \left| \frac{\partial \boldsymbol{\mu}}{\partial Q_k} \right|^2 = \sum_{\gamma \in \{x,y,z\}} \left(\frac{\partial \mu_\gamma}{\partial Q_k} \right)^2 \tag{S45}$$

S3.3 MD-Based IR Calculation

An alternative approach computes the IR spectrum from molecular dynamics via the Wiener-Khinchin theorem, capturing anharmonic effects.

S3.3.1 Dipole Autocorrelation Function

The dipole moment is recorded along an MD trajectory $\boldsymbol{\mu}(t)$. The autocorrelation function:

$$C_{\mu\mu}(\tau) = \langle \boldsymbol{\mu}(t) \cdot \boldsymbol{\mu}(t+\tau) \rangle_t \tag{S46}$$

S3.3.2 Wiener-Khinchin Theorem

The IR absorption spectrum is the Fourier transform of the dipole autocorrelation:

$$I(\omega) \propto \omega^2 \int_{-\infty}^{\infty} C_{\mu\mu}(\tau) e^{-i\omega\tau} d\tau \tag{S47}$$

In practice, the spectrum is evaluated as the power spectrum of the windowed, mean-subtracted dipole signal $\delta\boldsymbol{\mu}(t)$, which by the Wiener–Khinchin theorem is equivalent to Eq. (S47):

$$I(\omega_k) \propto \omega_k^2 \sum_{\gamma \in \{x,y,z\}} |\text{FFT}[W(t) \cdot \delta\mu_\gamma(t)]|^2 \tag{S48}$$

where $W(t)$ is a windowing function; the signal is mirrored and zero-padded to a power of two before the FFT.

S3.3.3 Windowing Functions

To reduce spectral leakage, the dipole signal is multiplied by a window function before the FFT; all windows are rescaled to preserve the signal power:

Table S4: Available windowing functions

Window	Definition
Hann (default)	$W(n) = 0.5\,[1 - \cos(2\pi n/N)]$
Hamming	$W(n) = 0.54 - 0.46\cos(2\pi n/N)$
Blackman-Harris	$W(n) = \sum_{m=0}^{3} (-1)^m a_m \cos(2\pi mn/N)$
Gaussian	$W(n) = \exp\big[-\frac{1}{2}\left((n - N/2)/(\sigma N/2)\right)^2\big]$
none	$W(n) = 1$

The window is selected with `--window` (default `hann`); the four-term Blackman-Harris coefficients are $a = (0.359, 0.488, 0.141, 0.012)$.

S3.3.4 MD Protocol

1. **NVT equilibration**: 5 ps at target temperature (default 300 K)
2. **NVE production**: 25 ps microcanonical trajectory (timestep $\Delta t = 0.5$ fs, `--md-timestep`)
3. **Dipole sampling**: Record $\boldsymbol{\mu}$ every 2.5 fs
4. **Trajectory segmentation**: Divide into overlapping segments ("chopping"; default 10 segments, `--chop`)
5. **Averaging**: Average spectra from all segments, and—when several replicas are requested—over independent replicas (see below)

Replica averaging. The MD-IR path supports N independent replicas per conformer (`--n-replicas`, default 1), launched with different random seeds and propagated in parallel via `vmap`; their spectra are averaged to reduce statistical noise. Optionally, an additional Gaussian smoothing of the final plotted spectrum is available through `--gaussian-sigma`, and the full NVE trajectory can be written to disk with `--trajectory-file`.

S3.4 Normal Mode Classification

Each normal mode is automatically classified by its dominant motion type using projection onto internal coordinates.

S3.4.1 Wilson B-Matrix Formalism

The Wilson B-matrix relates Cartesian displacements to internal coordinate changes:

$$\Delta q = \mathbf{B}\Delta\mathbf{x} \tag{S49}$$

where Δq is the change in internal coordinate (bond length, angle, or dihedral) and $\Delta\mathbf{x}$ is the Cartesian displacement vector.

Stretching coordinates. For a bond between atoms i and j:

$$B_i = -\hat{\mathbf{e}}_{ij}, \quad B_j = \hat{\mathbf{e}}_{ij} \tag{S50}$$

where $\hat{\mathbf{e}}_{ij} = (\mathbf{r}_j - \mathbf{r}_i)/|\mathbf{r}_j - \mathbf{r}_i|$.

Bending coordinates. For an angle i-j-k (central atom j):

$$B_i = \frac{\cos\theta \cdot \hat{\mathbf{e}}_{ji} - \hat{\mathbf{e}}_{jk}}{r_{ji}\sin\theta} \tag{S51}$$

$$B_k = \frac{\cos\theta \cdot \hat{\mathbf{e}}_{jk} - \hat{\mathbf{e}}_{ji}}{r_{jk}\sin\theta} \tag{S52}$$

$$B_j = -B_i - B_k \tag{S53}$$

Torsional coordinates. For a dihedral i-j-k-l:

$$B_i = -\frac{\mathbf{n}_1}{r_{ij}\sin^2\theta_1} \tag{S54}$$

$$B_l = \frac{\mathbf{n}_2}{r_{kl}\sin^2\theta_2} \tag{S55}$$

where $\mathbf{n}_1 = \hat{\mathbf{e}}_{ij} \times \hat{\mathbf{e}}_{jk}$ and $\mathbf{n}_2 = \hat{\mathbf{e}}_{jk} \times \hat{\mathbf{e}}_{kl}$ are the (unnormalized) plane normals and θ_1, θ_2 the bond angles at j and k.

S3.4.2 Mode Projection

For each normal mode, with normalized Cartesian displacement $\mathbf{d}_k = \mathbf{M}^{-1/2}\mathbf{L}_k/\|\mathbf{M}^{-1/2}\mathbf{L}_k\|$, the contribution from internal coordinate type t is:

$$S_{k,t} = \sum_{q\in\text{type } t} (\mathbf{B}_q \cdot \mathbf{d}_k)^2 \tag{S56}$$

The fractional contribution:

$$f_{k,t} = \frac{S_{k,t}}{S_{k,\text{str}} + S_{k,\text{bend}} + S_{k,\text{tors}}} \tag{S57}$$

S3.4.3 Classification Rules

A mode is classified based on the dominant contribution:

$$\text{Type}_k = \begin{cases} \text{stretching} & \text{if } f_{k,\text{str}} \geq 0.6 \\ \text{bending} & \text{if } f_{k,\text{bend}} \geq 0.6 \\ \text{torsion} & \text{if } f_{k,\text{tors}} \geq 0.6 \\ \text{mixed} & \text{otherwise} \end{cases} \tag{S58}$$

S3.4.4 Sub-Classification

Stretching modes:

- **Symmetric**: In-phase stretching of equivalent bonds
- **Asymmetric**: Out-of-phase stretching

Bending modes:

- **Symmetric deformation**: In-plane, in-phase angle opening/closing
- **Asymmetric deformation**: In-plane, out-of-phase motion
- **Wagging**: Out-of-plane, in-phase
- **Twisting**: Out-of-plane, out-of-phase

S3.5 Functional Group Identification

Functional groups are identified using graph pattern matching on the molecular topology, following the AccFG (Accurate Functional Group extraction) approach [7]. When Hessian-based IR peak allocation uses this identification, MARS prints the AccFG citation at run time.

S3.5.1 Molecular Graph Representation

The molecule is represented as a graph $G = (V, E)$ where:

- Vertices V: atoms with properties (element, hybridization, ring membership)
- Edges E: bonds with orders (single, double, triple, aromatic)

S3.5.2 Bond Order Assignment

Bond orders are assigned using valence rules:

Algorithm S10 Bond Order Assignment

Require: Molecular graph G, typical valences $\{v_Z\}$
Ensure: Bond orders $\{b_{ij}\}$

Initialize all $b_{ij} \leftarrow 1$ (single bonds)
Compute valence deficit: $d_i \leftarrow v_{Z_i} - \sum_j b_{ij}$
while $\exists$ bond (i, j) with $d_i > 0$ and $d_j > 0$ **do**
  Select bond with maximum $d_i + d_j$
  $b_{ij} \leftarrow b_{ij} + 1$
  Update deficits
end while
return $\{b_{ij}\}$

S3.5.3 Pattern Matching

Over 40 functional group patterns are defined, organized by category:

Table S5: Functional group categories and examples

Category	Examples
Carbonyl	Ketone, aldehyde, ester, amide, carboxylic acid
C=C bonds	Alkene, aromatic, allene
C≡C/C≡N	Alkyne, nitrile
C=N bonds	Imine, oxime, hydrazone
N-O bonds	Nitro, nitroso, N-oxide
S=O bonds	Sulfoxide, sulfone, sulfonamide
Alcohols/Ethers	Alcohol, phenol, ether, epoxide
Amines	Primary, secondary, tertiary, aniline
Thiols/Thioethers	Thiol, thioether, disulfide
Thiocarbonyl	Thioketone, thioamide (C=S)
P=O / P bonds	Phosphate, phosphonic acid, phosphonamide, phosphine
O-O / Si, B	Peroxide, silyl, boronic groups
Halogens	Alkyl/aryl/vinyl halide

S3.5.4 Hierarchy Resolution

When multiple patterns match overlapping atoms, specific patterns take precedence over generic ones:

- Ester C=O suppresses standalone carbonyl
- Amide N-H suppresses generic amine
- Carboxylic acid O-H suppresses generic alcohol

S3.5.5 Mode-to-Functional-Group Mapping

For each vibrational mode, the dominant atoms are identified by displacement magnitude. These atoms are matched against detected functional groups to generate labels such as "C=O stretch (ester)" or "N-H bend (amide)".

S3.6 Multi-Molecule Mode Attribution

For systems containing multiple molecular fragments (e.g., solvated molecules, host-guest complexes), modes are attributed to specific fragments or classified as collective.

S3.6.1 Fragment Detection

Molecular fragments are identified via connected-component analysis on the covalent bond graph:

Algorithm S11 Fragment Detection

Require: Bond list, atom count N
Ensure: Fragment assignments $\{f_i\}_{i=1}^{N}$

Build adjacency list from bonds
$f_i \leftarrow -1$ for all atoms (unassigned)
frag_id $\leftarrow 0$
for $i = 1$ **to** N **do**
    if $f_i = -1$ **then**
        BFS/DFS from atom i, assign $f_j \leftarrow$ frag_id for all reachable j
        frag_id $\leftarrow$ frag_id $+ 1$
    end if
end for
return $\{f_i\}$

S3.6.2 Fragment Kinetic Energy Contribution

For each mode k, the kinetic energy contribution from fragment F is:

$$\mathrm{KE}_{k,F} = \sum_{i \in F} m_i \, |\mathbf{d}_{k,i}|^2 \tag{S59}$$

The fractional contribution:

$$\phi_{k,F} = \frac{\mathrm{KE}_{k,F}}{\sum_{F'} \mathrm{KE}_{k,F'}} \tag{S60}$$

S3.6.3 Mode Attribution

A mode is classified based on its fragment distribution:

$$\mathrm{Attribution}_k = \begin{cases} \text{intramolecular (fragment } F) & \text{if } \phi_{k,F} \geq 0.80 \\ \text{collective/intermolecular} & \text{otherwise} \end{cases} \tag{S61}$$

S3.6.4 Hydrogen Bond Detection

Intermolecular modes are further analyzed for hydrogen-bonding character by checking if the dominant motion involves:

- Donor/acceptor heavy atoms drawn from {O, N, F}, on different fragments
- A hydrogen-to-acceptor contact in the range $1.2 < d_{\mathrm{H}\cdots\mathrm{X}} < 2.5$ Å, or a heavy-atom donor$\cdots$acceptor distance in the range $2.0 < d_{\mathrm{X}\cdots\mathrm{Y}} < 3.5$ Å

S3.7 Spectrum Output and Visualization

Once frequencies, intensities, and mode classifications have been computed, MARS offers two built-in ways to inspect the result: a static, publication-style plot generated with the `--plot` flag, and an interactive three-dimensional explorer launched with `mars viewer --ir`. We illustrate both on methanol (CH_3OH), whose harmonic spectrum is obtained from a single command:

```
mars ir methanol.xyz --plot --mode-xyz --save-structure
```

Here `--plot` writes the annotated spectrum figure, while `--mode-xyz` and `--save-structure` export the per-mode eigenvectors (`normal_modes.xyz`), the broadened spectrum (`ir_spectrum.dat`), the mode classification (`mode_analysis.txt`), and the optimized geometry that the viewer later reads back.

S3.7.1 Static Plot (`--plot`)

The `--plot` flag produces a two-panel figure (Figure S1). The upper panel shows the raw stick spectrum—one line per normal mode at its harmonic frequency, with height proportional to the IR intensity—and each stick is annotated with the dominant internal coordinate of the mode (e.g. C–O, C–H, O–H). The lower panel overlays a Lorentzian-broadened envelope (default width $\gamma = 10$ cm^{-1}, adjustable with `--broadening`), which approximates an experimentally measured band shape. An information box reports the temperature, the potential energy of the structure, and the number of modes assigned to each motion type (stretch / bend / torsion / mixed). The plotting window and resolution are controlled by `--freq-range MIN MAX` and `--plot-output FILE`; without the latter the figure is written next to the input as `ir_spectrum.png`.

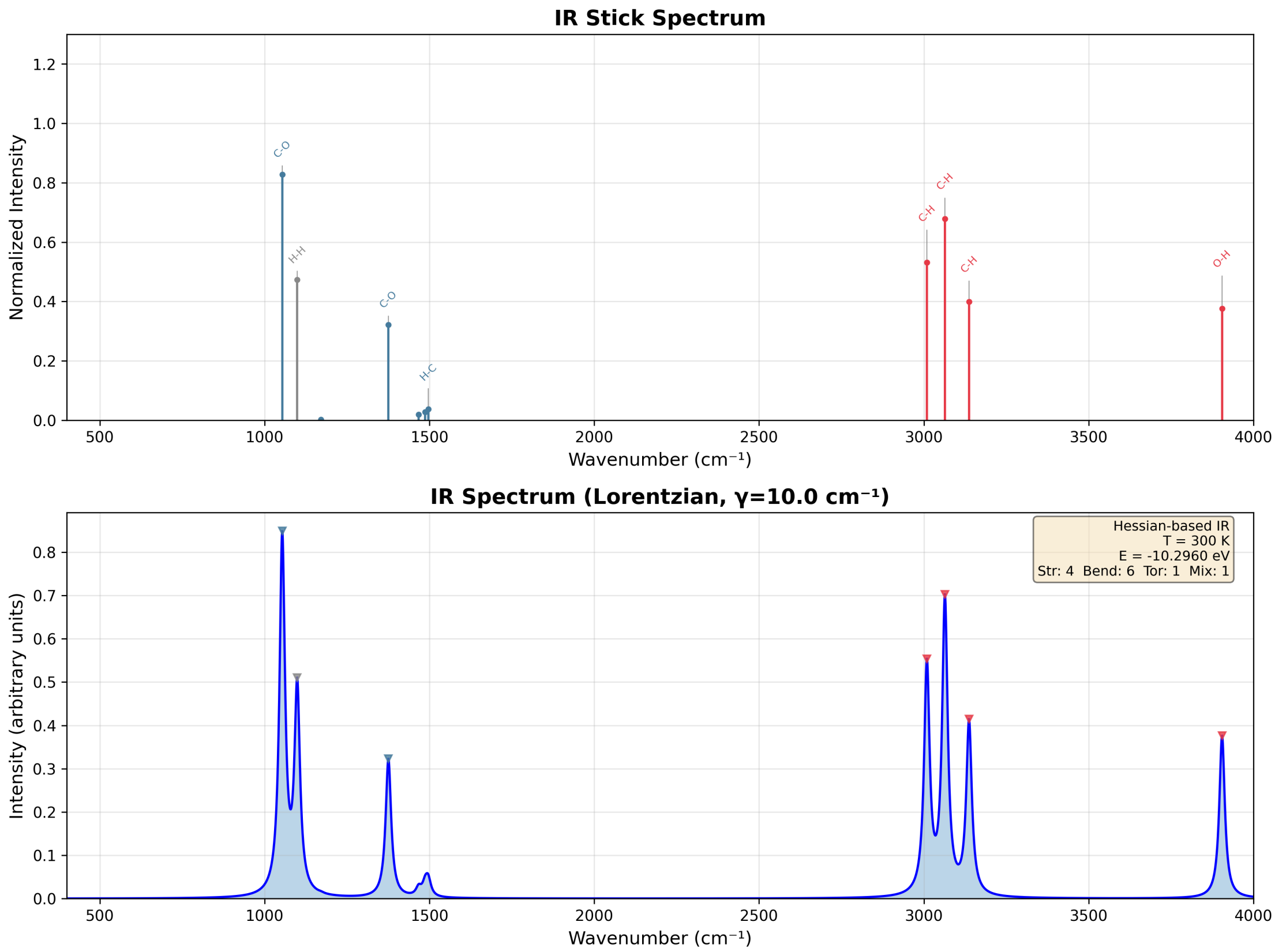

Figure S1: Static IR output of `mars ir methanol.xyz --plot`. Top: stick spectrum with per-mode internal-coordinate labels. Bottom: Lorentzian-broadened spectrum ($\gamma = 10$ cm^{-1}, $T = 300$ K); the info box summarizes the structure energy and the stretch/bend/torsion/mixed mode counts. Colors follow the mode classification of Section S3.4.

S3.7.2 Interactive Explorer (`mars viewer --ir`)

For a closer look at individual vibrations, `mars viewer --ir` opens an interactive explorer (Figure S2) that auto-discovers the result files produced above (or accepts explicit `--modes`, `--spectrum`, and `--mode-analysis` paths, or a `--dir`). The window couples three views:

- a 3D rendering of the molecule with per-atom displacement arrows for the selected mode;
- an information panel reporting the mode index, frequency, normalized intensity, and classified type;
- a clickable IR spectrum—selecting a peak jumps to the corresponding mode.

Modes can be stepped through with the `Prev`/`Next` buttons or the arrow keys, and the `Animate` button toggles a real-time oscillation of the structure along the eigenvector. Sliders adjust the arrow length, animation amplitude, atom size, and bond width. The same command renders headlessly to an image with `--save FILE`, which is how the snapshot in Figure S2 was produced; this is useful on remote machines without a display.

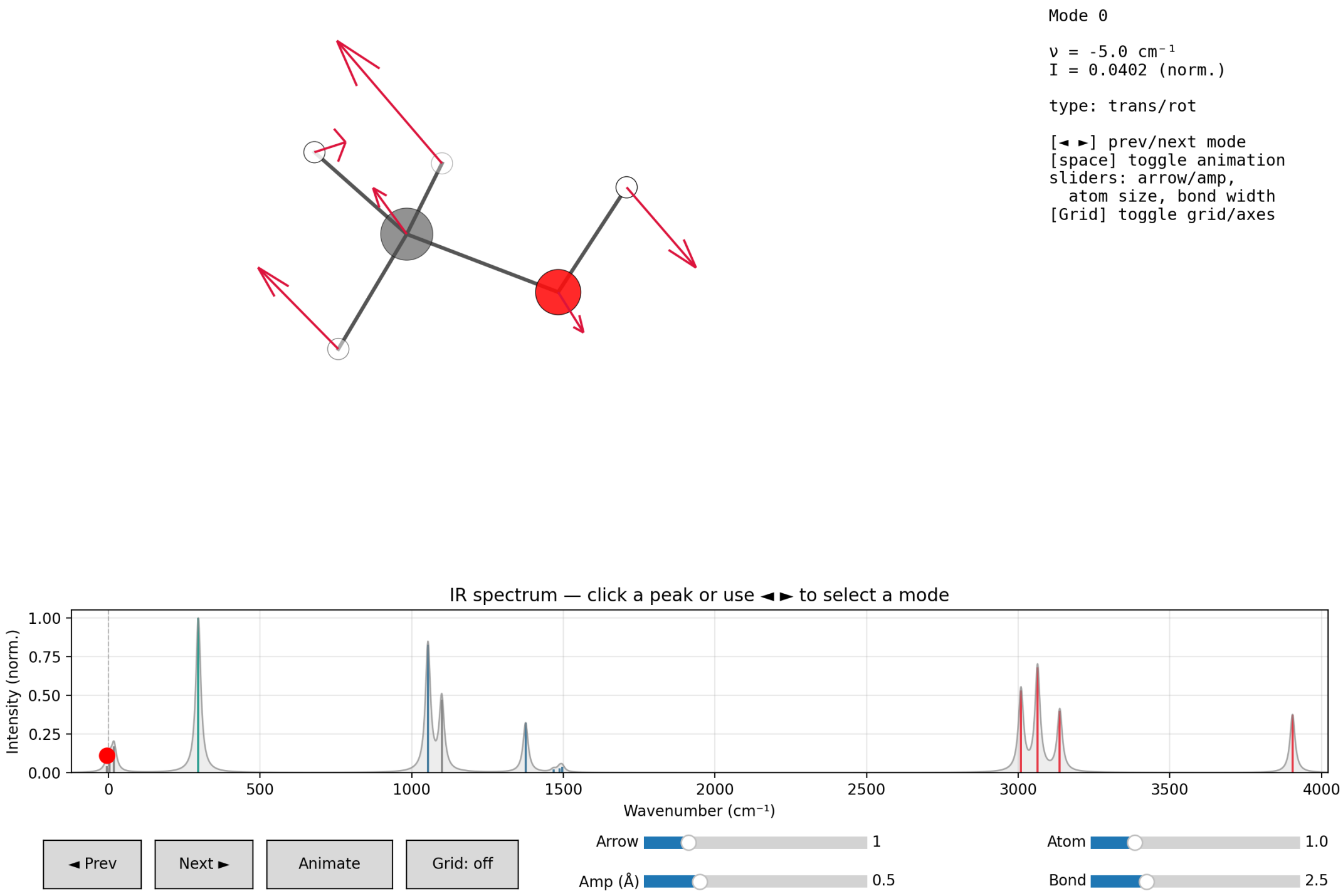

Figure S2: Interactive IR explorer (`mars viewer --ir`) for methanol, rendered headlessly with `--save`. The 3D panel shows the molecule with red displacement arrows for the currently selected normal mode; the side panel reports the mode index, frequency, intensity, and type; and the lower panel is a clickable IR spectrum. Modes are navigated with the `Prev`/`Next` buttons or arrow keys, and `Animate` oscillates the structure along the eigenvector.

S3.7.3 Additional Options

Several flags extend the IR workflow beyond the single-structure default:

- `--all-conformers`: compute a harmonic spectrum for every frame of a multi-conformer XYZ file, emitting per-conformer outputs for Boltzmann-weighted analysis.
- `--atoms`: restrict the analysis to a subset of atoms (e.g. the solute in a microsolvated cluster). For the Hessian path this builds a partial $3K \times 3K$ Hessian over the K selected atoms; for the MD path the dipole $\boldsymbol{\mu}(t) = \sum_i q_i \mathbf{r}_i$ is summed over the selected atoms only.
- `--fd-hessian`: force the finite-difference Hessian even when autodiff is available.
- `--save-hessian`: write the full Hessian / force-constant data (`ir_hessian_data.txt`) for external post-processing.

S4 Microsolvation

The microsolvation module constructs explicit solvent shells around molecular solutes. Two complementary build strategies are available. The default *geometric layer-by-layer* strategy

(Sections S4.1–S4.6) packs concentric shells directly around the solute using Fibonacci-sphere candidate generation with van der Waals overlap rejection. The alternative *barostat* strategy (Section S4.7) instead compresses a solute-free solvent droplet inside a spherical moving wall and then swaps the solute into a reserved central cavity, producing liquid-like packing densities. Both strategies share the same solvent library, frozen-solute relaxation, and topology-repair machinery.

S4.1 Solvent Placement Algorithm

Solvent molecules are placed in concentric layers around the solute using a three-stage process: candidate generation, overlap rejection, and greedy selection.

S4.1.1 Candidate Generation: Fibonacci Sphere Sampling

Candidate positions are generated uniformly on spherical shells using the Fibonacci spiral method, which provides near-optimal uniform distribution without clustering at poles.

For N points on a unit sphere:

$$\phi_i = 2\pi i/\varphi \tag{S62}$$

$$z_i = 1 - \frac{2i+1}{N} \tag{S63}$$

$$r_i = \sqrt{1 - z_i^2} \tag{S64}$$

where $\varphi = (1+\sqrt{5})/2$ is the golden ratio, and the Cartesian coordinates are:

$$(x_i, y_i, z_i) = (r_i \cos\phi_i, r_i \sin\phi_i, z_i) \tag{S65}$$

Radial shells. For each solute atom, three concentric shells of candidates are generated at radii:

$$R_{\text{shell}}^{(k)} = R_{\text{vdW,atom}} + R_{\text{solvent}} + k \cdot \Delta R, \quad k \in \{0, 1, 2\} \tag{S66}$$

where $R_{\text{vdW,atom}}$ is the atom's van der Waals radius, R_{solvent} is the solvent's effective (cross-sectional) packing radius, and the shell spacing is $\Delta R = 2 \times R_{\text{solvent}}$.

Default: 300 Fibonacci candidate points per atom per shell in the automatic workflow (`auto_solvate`; 200 in the manual layer builder).

S4.1.2 Van der Waals Overlap Rejection

Candidates overlapping with existing atoms (solute or previously placed solvent) are rejected:

$$\text{Reject if: } d_{ij} < s_{\text{vdW}} \cdot (R_{\text{vdW},i} + R_{\text{vdW},j}) \tag{S67}$$

where d_{ij} is the distance between atoms i and j, and s_{vdW} is the overlap scale factor.

Default: $s_{\text{vdW}} = 0.75$ for the geometric layer-by-layer builder (the barostat builder uses 0.85).

S4.1.3 Greedy Selection

From non-overlapping candidates, solvent molecules are selected to maximize coverage while maintaining spacing:

Algorithm S12 Greedy Solvent Selection

Require: Candidates $\mathcal{C}$, target count N_{target}, weight w
Ensure: Selected positions $\mathcal{S}$

$\mathcal{S} \leftarrow \emptyset$
Compute proximity scores: $p_i = 1/d^{(i)}_{\text{solute}}$ for each candidate
while $|\mathcal{S}| < N_{\text{target}}$ and $\mathcal{C} \neq \emptyset$ **do**
    Compute angular spread: $a_i = \min_{j \in \mathcal{S}} \angle(\mathbf{r}_i, \mathbf{r}_j)$ from solute center (normalized by π)
    Combined score: $\text{score}_i = p_i + w \cdot a_i$
    Select $i^* = \arg\max_i \text{score}_i$
    $\mathcal{S} \leftarrow \mathcal{S} \cup \{i^*\}$
    Remove candidates overlapping with i^* from $\mathcal{C}$
end while
return $\mathcal{S}$

The weight w (`spread_weight`, default 1.0) sets how strongly angular spread is favored relative to proximity; the proximity term is always retained, so larger w promotes more uniform angular coverage without discarding the proximity preference.

S4.2 Layer-by-Layer Shell Construction

Solvent shells are built iteratively, with each layer using the combined solute+inner-shell atoms as the reference for candidate generation.

S4.2.1 Manual Mode

Fixed number of molecules per layer with explicit control:

Algorithm S13 Manual Layer Construction

Require: Solute $\mathcal{M}_0$, layer specifications $\{(n_\ell, s_\ell)\}_{\ell=1}^{L}$
Ensure: Solvated system $\mathcal{M}$

$\mathcal{M} \leftarrow \mathcal{M}_0$
for $\ell = 1$ **to** L **do**
    $\mathcal{C} \leftarrow \text{GenerateCandidates}(\mathcal{M}, R_{\text{solvent}})$
    $\mathcal{C} \leftarrow \text{RejectOverlaps}(\mathcal{C}, \mathcal{M}, s_\ell)$
    $\mathcal{S}_\ell \leftarrow \text{GreedySelect}(\mathcal{C}, n_\ell)$
    $\mathcal{M} \leftarrow \mathcal{M} \cup \mathcal{S}_\ell$
end for
return $\mathcal{M}$

S4.2.2 Automatic Mode

In automatic mode each concentric shell is filled densely—every non-overlapping candidate that also clears the previously placed solvent is accepted (with up to `refill_cycles` repacking passes per shell)—and shells are added outward until a stopping criterion is met:

Algorithm S14 Automatic Shell Growth

Require: Solute $\mathcal{M}_0$; either a target padding d_{pad} or a target count N_{target}

Ensure: Solvated system $\mathcal{M}$

```
1: M ← M_0,    shell index s ← 0
2: repeat
3:     C ← GenerateCandidates(M_0, s)        ▷ Fibonacci shell at radius r_pack + s · 2r_pack
4:     C ← RejectOverlaps(C, M)
5:     M ← M ∪ DenseFill(C);    optionally relax shell (solute frozen)
6:     s ← s + 1
7: until stopping criterion
8: return M
```

Two stopping criteria are available. In **cover** mode (default, or when `padding` is given) shells are added until the shell offset reaches the requested thickness, $r_{\text{pack}} + s \cdot 2r_{\text{pack}} \geq d_{\text{pad}}$; with no `padding` the build stops after the first saturated contact shell that fully wraps the solute. In **count** mode (`n_molecules` given) shells grow until at least the requested number of molecules is placed, trimming the final shell to the molecules closest to the solute. Shell growth is also capped by `max_layers`.

S4.3 Solvent Library

Solvent geometries (curated experimental structures or Open Babel/MMFF94 3D structures) are stored with associated metadata.

S4.3.1 Available Solvents

Table S6: Solvent library (representative selection)

Category	Solvents	Count
Protic polar	Water, methanol, ethanol, *n*-/iso-propanol, *n*-/tert-butanol, ethylene glycol, glycerol, trifluoroethanol	10
Aprotic polar	Acetonitrile, acetone, DMF, DMSO, NMP, nitromethane	6
Halogenated	Chloroform, DCM, carbon tetrachloride	3
Ethers/esters	THF, dioxane, diethyl ether, ethyl acetate	4
Aromatic	Benzene, toluene, pyridine	3
Alkanes	*n*-pentane, hexane, heptane, cyclohexane	4
Other	Ammonia, formamide, HF, CS_2	4
**Total**		**34**

The exact list is obtained at runtime with `mars.solvents.list_solvents()`.

S4.3.2 Solvent Properties

Each solvent entry stores:

- XYZ geometry
- Molecular formula and SMILES
- Bulk density (for estimating shell counts)
- Dielectric constant (for property estimation)

- Aliases and a provenance/source tag

The effective (packing) radius used during placement is computed on the fly from the geometry rather than stored.

S4.3.3 Custom Solvents

User-defined solvents are registered by providing an XYZ file plus optional metadata:

```
register_solvent(name, xyz_path, aliases=..., formula=...,
                 density=..., dielectric=..., source=...)
```

S4.4 Shell Optimization

After geometric placement, the solvent shell is relaxed while keeping the solute frozen.

S4.4.1 Frozen-Atom Optimization

The solute atoms are held fixed by zeroing their gradient components:

$$\mathbf{g}_{\text{masked}} = \begin{cases} \mathbf{0} & \text{if atom} \in \text{solute} \\ \mathbf{g} & \text{if atom} \in \text{solvent} \end{cases} \tag{S68}$$

This is implemented via element-wise multiplication with a binary mask.

S4.4.2 Two-Stage Relaxation

Each relaxation is run in two stages: a coarse pass that quickly resolves clashes followed by a fine pass that converges to the minimum.

1. **Coarse stage**: $f_{\max} = 5 \times f_{\text{target}}$, using 40% of the iteration budget.
2. **Fine stage**: $f_{\max} = f_{\text{target}}$, using the remaining iterations.

The optimizer is selected with `method` (default `FIRE`); both stages use that method. The `HYBRID` option additionally switches the coarse stage to FIRE and the fine stage to L-BFGS, combining FIRE's robustness on clashes with L-BFGS's fast final convergence.

S4.5 Topology Repair

Even after geometric overlap rejection and constrained relaxation, individual solvent molecules occasionally fragment, reorganize, or chemically react with the solute during shell optimization—particularly with reactive backends, charged solutes, or aggressive optimizer settings. The microsolvation module therefore performs an automated topology repair loop after relaxation, in which broken solvent molecules are detected, removed, and replaced by fresh molecules placed in the vacated positions before re-relaxation. The loop iterates until all solvent molecules retain their reference covalent topology, or until a maximum number of repair passes is reached.

S4.5.1 Reference Topology

The covalent topology of an isolated solvent molecule is precomputed once from the library geometry. Bonds are detected from interatomic distances using element-specific covalent radii $r_{\text{cov}}^{(i)}$ with a multiplicative tolerance τ:

$$d_{ij} \leq \tau \cdot \left(r_{\text{cov}}^{(i)} + r_{\text{cov}}^{(j)} \right) \tag{S69}$$

The resulting set of bonded atom pairs $\mathcal{B}_{\text{ref}}$ defines the reference topology shared by every solvent molecule in the shell.

Default: $\tau = 1.3$ (`topo_tolerance`).

S4.5.2 Broken-Molecule Detection

For each solvent molecule $\mathcal{S}_k$ in the relaxed shell, the bond set $\mathcal{B}_k$ is recomputed from the current positions using Eq. S69. A solvent molecule is flagged as *broken* if any of the following conditions hold:

- $\mathcal{B}_k \neq \mathcal{B}_{\text{ref}}$ (covalent connectivity changed);
- the molecule has fragmented into disjoint components under the connectivity graph;
- one or more atoms of $\mathcal{S}_k$ formed a new bond to the solute or to a different solvent molecule.

S4.5.3 Repair Loop

Algorithm S15 Topology Repair Loop

Require: Solvated system $\mathcal{M} = (\mathcal{M}_0, \mathcal{S})$, reference bonds $\mathcal{B}_{\text{ref}}$, target count N_{target}, max passes K

Ensure: Repaired solvated system $\mathcal{M}'$

for $k = 1$ to K **do**
    $\mathcal{S}_{\text{broken}} \leftarrow \text{DetectBrokenMolecules}(\mathcal{S}, \mathcal{B}_{\text{ref}})$
    if $\mathcal{S}_{\text{broken}} = \emptyset$ **then**
        return $\mathcal{M}$ ▷ Converged
    end if
    $\mathcal{S} \leftarrow \mathcal{S} \setminus \mathcal{S}_{\text{broken}}$ ▷ Remove broken molecules
    $\mathcal{S} \leftarrow \text{Refill}(\mathcal{S}, \mathcal{M}_0, N_{\text{target}})$ ▷ Re-run shell construction
    $\mathcal{S} \leftarrow \text{OptimizeShell}(\mathcal{S}, \mathcal{M}_0)$ ▷ Re-relax
end for
$\mathcal{S} \leftarrow \mathcal{S} \setminus \text{DetectBrokenMolecules}(\mathcal{S}, \mathcal{B}_{\text{ref}})$ ▷ Drop survivors
return $\mathcal{M}$

Each repair pass calls the same dense-fill shell construction routine used for the initial build, seeded from the current (partially depleted) system so that fresh molecules occupy the space vacated by the removed ones while overlap rejection protects the surviving solvent. The seed used by the Fibonacci candidate generator is incremented at every pass so that successive refills explore different orientations rather than reproducing the broken configuration. If the loop has not converged after K passes, any molecules still broken in the final pass are dropped from the output structure with a warning, ensuring that the returned solvated system is always topologically consistent.

S4.5.4 Defaults and Toggle

Topology repair is enabled by default and is controlled by three parameters of `auto_solvate`:

- `topology_repair`: enables/disables the loop (default: `True`);
- `max_repair`: maximum number of repair passes (default: 3);
- `topo_tolerance`: bond-detection tolerance τ in Eq. S69 (default: 1.3).

In practice, the vast majority of shells converge in zero or one pass; the loop becomes important for highly polar or reactive solute environments, where occasional proton transfers or solvent fragmentation events would otherwise contaminate the ensemble.

S4.6 Multi-Conformer Solvation

For conformational ensembles, the solvent shell can be reused across conformers via transplantation.

S4.6.1 Shell Transplantation

Given a solvated reference structure and a new solute conformer:

Algorithm S16 Solvent Shell Transplantation

Require: Reference solvated system $(\mathcal{M}_{\text{ref}}, \mathcal{S}_{\text{ref}})$, new solute $\mathcal{M}_{\text{new}}$
Ensure: New solvated system $(\mathcal{M}_{\text{new}}, \mathcal{S}_{\text{new}})$
1: Align $\mathcal{M}_{\text{new}}$ to $\mathcal{M}_{\text{ref}}$ via Kabsch algorithm
2: $\mathcal{S}_{\text{new}} \leftarrow \mathcal{S}_{\text{ref}}$ ▷ Copy solvent positions
3: Optimize $\mathcal{S}_{\text{new}}$ with $\mathcal{M}_{\text{new}}$ frozen
4: **return** $(\mathcal{M}_{\text{new}}, \mathcal{S}_{\text{new}})$

S4.6.2 Kabsch Alignment

The new solute is aligned to the reference using the Kabsch algorithm [1] to minimize RMSD:

$$\mathbf{R}_{\text{rot}} = \arg \min_{\mathbf{R} \in SO(3)} \sum_i \|\mathbf{R}\mathbf{r}_i^{\text{new}} - \mathbf{r}_i^{\text{ref}}\|^2 \tag{S70}$$

The optimal rotation is computed via SVD of the cross-covariance matrix:

$$\mathbf{H} = \sum_i (\mathbf{r}_i^{\text{new}} - \bar{\mathbf{r}}^{\text{new}})(\mathbf{r}_i^{\text{ref}} - \bar{\mathbf{r}}^{\text{ref}})^T \tag{S71}$$

$$\mathbf{H} = \mathbf{U}\mathbf{\Sigma}\mathbf{V}^T \quad \Rightarrow \quad \mathbf{R}_{\text{rot}} = \mathbf{V}\mathbf{U}^T \tag{S72}$$

S4.6.3 Batch Solvation

Multiple conformers can be solvated in parallel:

1. Solvate the lowest-energy conformer (reference)
2. Transplant shell to remaining conformers
3. Batch-optimize all shells simultaneously (Section S2.4)

Batch uniformization. Vectorized (`vmap`) batch optimization requires every structure in a batch to share the same atom count, but independent solvation, shell transplantation, and broken-molecule removal can each leave conformers with slightly different solvent counts. Before batching—and again after the final topology cleanup—the ensemble is therefore uniformized against the target count N_{target}, across all build paths (independent, transplant, and barostat): structures already at N_{target} are left untouched; structures above it are trimmed to the N_{target} molecules closest to the solute; and structures below it are repaired in up to two rounds of *remove-broken* → *geometric refill to* N_{target} → *frozen-solute relaxation*. If a conformer is genuinely geometry-limited and cannot reach N_{target}, a warning is issued and the whole batch is trimmed to the smallest achieved count so all shapes stay uniform.

S4.7 Barostat (Moving-Wall Droplet) Solvation

The geometric layer-by-layer strategy places solvent directly onto the solute surface, which is robust but tends to produce loosely packed shells whose density is governed by the placement geometry rather than by the potential. The *barostat* strategy reaches liquid-like packing by mechanically compressing a separately prepared solvent droplet and only then introducing the solute. It is selected with the `--barostat` flag and requires a target solvent count (`--n-molecules`).

The procedure mimics a spherical piston acting on a solvent droplet, with the wall reaction force playing the role of an applied pressure. It proceeds in four stages (Algorithm S17): droplet placement, barostat compression, solute swap, and topology cleanup.

S4.7.1 Spherical Wall Potentials

Two soft confining walls are added to the potential energy as differentiable barriers. The *outer wall* (the piston) penalizes atoms beyond a radius r_{out}, while the *inner wall* reserves a solute-shaped cavity by penalizing atoms inside a radius r_{in}:

$$E_{\text{wall}}(\mathbf{R}) = k \sum_i \left[\max\left(0, \frac{d_i}{r_{\text{out}}} - 1\right)^n + \max\left(0, 1 - \frac{d_i}{r_{\text{in}}}\right)^n \right], \tag{S73}$$

where $d_i = \|\mathbf{r}_i - \mathbf{c}\|$ is the distance of atom i from the droplet center $\mathbf{c}$, k is the wall force constant (`--baro-k`, default 5.0 eV), and the exponent n selects a soft harmonic wall ($n = 2$, default) or a steep wall ($n = 12$) via `--baro-wall-mode`. Both terms act during droplet compression, keeping the central cavity empty; the inner term is removed once the solute is inserted.

The cavity radius is set from the solute bounding radius R_{solute} and a clearance buffer (`--baro-inner-buffer`, default 2.0 Å):

$$r_{\text{in}} = R_{\text{solute}} + \Delta_{\text{buffer}}. \tag{S74}$$

The initial outer radius is either set explicitly (`--baro-radius`) or estimated from the requested molecule count N and a target fill fraction ϕ (`--baro-fill`, default 0.35):

$$r_{\text{out}}^{(0)} = \left(r_{\text{in}}^3 + \frac{N\, r_{\text{mol}}^3}{\phi} \right)^{1/3}, \tag{S75}$$

where r_{mol} is the solvent bounding radius. A floor $r_{\text{out}} \geq r_{\text{in}} + 2r_{\text{eff}}$ prevents over-compression below a single solvent layer.

S4.7.2 Barostat Compression

The droplet is compressed iteratively. At each step the solvent is relaxed against the current walls, the outer-wall reaction force is measured, and the outer radius is shrunk by a fixed increment Δr (`--baro-step`, default 0.5 Å). The reaction force is the peak per-atom force exerted by the outer wall,

$$F_{\text{wall}} = \max_i \left\| \nabla_{\mathbf{r}_i} E_{\text{wall}}^{\text{out}}(\mathbf{R}) \right\|, \tag{S76}$$

and serves as the convergence gauge: compression stops when F_{wall} reaches the target "pressure" $F_{\max}$ (`--baro-max-force`, default 1.0 eV/Å), when the radius floor is hit, or after `--baro-max-steps` iterations (default 40).

Optionally (`--baro-md`), each *droplet* compression step first runs a short NVT molecular dynamics trajectory with the wall added to the energy, allowing the solvent to flow past itself like a liquid before the FIRE settle; this typically yields more homogeneous packing for larger droplets. MD-driven compression is applied only while the droplet is solute-free; the post-swap final packing (with the solute frozen) always uses plain FIRE relaxation. The MD temperature, duration, and timestep are controlled by `--baro-md-temp` (300 K), `--baro-md-time` (0.3 ps), and `--baro-md-dt` (0.5 fs).

S4.7.3 Solute Swap and Cleanup

Once compressed, the solute is inserted at the droplet center (which the inner wall kept empty), the inner wall is removed, and, with the solute frozen (Eq. S68), the solvent is compressed onto the solute surface with the moving outer wall and finally relaxed without wall. Finally, a topology check (Section S4.5) removes any solvent molecule whose covalent connectivity broke during relaxation. Unlike the geometric mode, the barostat performs removal only—no refill—since the droplet count is fixed in advance.

Algorithm S17 Barostat Solvation

Require: Solute $\mathcal{M}_0$, solvent type, count N, target force $F_{\max}$, step Δr
Ensure: Solvated system $\mathcal{M}$

$\mathbf{c} \leftarrow \text{centroid}(\mathcal{M}_0)$; $\quad r_{\text{in}} \leftarrow R_{\text{solute}} + \Delta_{\text{buffer}}$
$r_{\text{out}} \leftarrow$ initial outer radius (from ϕ or `--baro-radius`)
$\mathcal{S} \leftarrow$ place N solvent molecules in shell $[r_{\text{in}}, r_{\text{out}}]$ ▷ no solute
for step $= 1$ to `max_steps` **do**
  (optional) short NVT MD of $\mathcal{S}$ with walls $\{r_{\text{in}}, r_{\text{out}}\}$
  relax $\mathcal{S}$ with walls $\{r_{\text{in}}, r_{\text{out}}\}$ ▷ Eq. S73
  $F_{\text{wall}} \leftarrow \max_i \|\nabla E_{\text{wall}}^{\text{out}}\|$
  if $F_{\text{wall}} \geq F_{\max}$ **or** $r_{\text{out}} - \Delta r < r_{\text{floor}}$ **then**
    break
  end if
  $r_{\text{out}} \leftarrow r_{\text{out}} - \Delta r$
end for
insert $\mathcal{M}_0$ in cavity; drop inner wall; relax with $\mathcal{M}_0$ frozen, outer wall on
remove solvent molecules with broken topology
return $\mathcal{M}_0 \cup \mathcal{S}$

S5 Non-Covalent Interactions

The non-covalent interaction (NCI) module enables conformational sampling of multi-fragment systems such as host-guest complexes, ion pairs, and molecular clusters. The approach uses geometric confinement potentials and fragment-aware filtering rather than explicit interaction detection.

S5.1 Multi-Fragment System Detection

Systems containing multiple molecular fragments are automatically detected via connectivity analysis.

S5.1.1 Covalent Bond Detection

Bonds are identified based on interatomic distances relative to covalent radii:

$$\text{Bond}(i, j) \Leftrightarrow d_{ij} < f_{\text{tol}} \cdot (R_{\text{cov},i} + R_{\text{cov},j}) \tag{S77}$$

where $R_{\text{cov},i}$ is the covalent radius of atom i and f_{tol} is the tolerance factor.
Default: $f_{\text{tol}} = 1.3$.

S5.1.2 Connected Component Analysis

Molecular fragments are identified as connected components in the bond graph:

Algorithm S18 Fragment Identification

Require: Atom positions $\{\mathbf{r}_i\}$, covalent radii $\{R_{\text{cov},i}\}$
Ensure: Fragment assignments $\{f_i\}$, number of fragments N_F
1: Detect bonds using Eq. S77
2: Build adjacency list $\mathcal{A}$
3: Compute connected components of $\mathcal{A}$ by union–find (disjoint-set with path compression and union by rank)
4: $f_i \leftarrow$ component id of atom i; $\quad N_F \leftarrow$ number of components
5: **return** $\{f_i\}$, N_F

A system is classified as multi-molecular if $N_F > 1$. (An equivalent BFS/DFS traversal would give the same components; the implementation uses union–find.)

S5.2 Confinement Potential

To prevent fragment dissociation during molecular dynamics and metadynamics, a confinement potential restricts atoms to a bounded region.

S5.2.1 Confinement Geometry

Two geometries are supported:

Spherical confinement. Atoms are confined within a sphere of radius R centered at $\mathbf{c}$:

$$d_i = \frac{|\mathbf{r}_i - \mathbf{c}|}{R} \tag{S78}$$

Ellipsoidal confinement. Atoms are confined within an axis-aligned ellipsoid:

$$d_i = \sqrt{\left(\frac{x_i - c_x}{R_x}\right)^2 + \left(\frac{y_i - c_y}{R_y}\right)^2 + \left(\frac{z_i - c_z}{R_z}\right)^2} \tag{S79}$$

In both cases, atoms with $d_i \leq 1$ are inside the confinement region.

S5.2.2 Confinement Energy Functions

Wall mode (default). Steep polynomial repulsion simulating a hard wall:

$$E_{\text{conf}}^{(i)} = k \cdot \max(0, d_i - 1)^n \tag{S80}$$

Default exponent: $n = 12$ (approximates hard-sphere behavior). This is the default confinement mode used by the `--nci` workflow.

Harmonic mode. Smooth quadratic penalty outside the boundary ($n = 2$):

$$E_{\text{conf}}^{(i)} = k \cdot \max(0, d_i - 1)^2 \tag{S81}$$

This provides elastic repulsion that gradually increases as atoms cross the boundary, and is selected with `--nci-mode harmonic`. The force constant defaults to $k = 10$ eV.

S5.2.3 Total Confinement Energy

The total confinement energy is summed over all atoms:

$$E_{\text{conf}} = \sum_{i=1}^{N} E_{\text{conf}}^{(i)} \tag{S82}$$

S5.2.4 Automatic Boundary Determination

The confinement boundary is automatically sized based on the molecular extent plus a buffer:

$$R = \max_i |\mathbf{r}_i - \mathbf{c}| + d_{\text{buffer}} \tag{S83}$$

Default buffer: $d_{\text{buffer}} = 3.0$ Å.
For ellipsoidal confinement, radii are computed independently along each axis.

S5.3 Inter-Fragment Distance Filtering

Conformers where fragments have drifted apart are filtered based on minimum inter-fragment distances.

S5.3.1 Cross-Fragment Distance Mask

A boolean mask $\mathbf{M}$ identifies atom pairs belonging to different fragments:

$$M_{ij} = \begin{cases} 1 & \text{if } f_i \neq f_j \\ 0 & \text{if } f_i = f_j \end{cases} \tag{S84}$$

S5.3.2 Minimum Contact Distance

The minimum inter-fragment contact distance:

$$d_{\text{contact}} = \min_{i,j:M_{ij}=1} |\mathbf{r}_i - \mathbf{r}_j| \tag{S85}$$

S5.3.3 Adaptive Distance Threshold

The maximum allowed contact distance is relaxed during early optimization cycles and tightened progressively:

Table S7: Adaptive NCI distance thresholds by optimization cycle

Cycle	Threshold Multiplier	Effective d_{max}
1	2.0×	$2.0 \times d_{\text{base}}$
2	1.5×	$1.5 \times d_{\text{base}}$
3	1.0×	$1.0 \times d_{\text{base}}$

The base threshold is $d_{\text{base}} = \max(d_{\text{init}}, \texttt{nci\_max\_dist})$, i.e. the larger of the initial closest inter-fragment contact d_{init} (measured on the input complex) and the user parameter `nci_max_dist` (default 3.0 Å). Anchoring to the initial contact prevents over-tight filtering of complexes whose fragments start farther apart than 3 Å.

S5.3.4 Filtering Rule

Structures are discarded if fragments are too far apart:

$$\text{Discard if: } d_{\text{contact}} > d_{\text{max}}^{(\text{cycle})} \tag{S86}$$

S5.4 Permutation-Invariant RMSD

For systems with equivalent fragments (e.g., two identical solvent molecules), standard RMSD may incorrectly flag reordered configurations as different conformers.

S5.4.1 Fragment Permutations

For N_F fragments of which N_{equiv} are chemically equivalent, the permutations of equivalent fragments are considered:

$$\text{RMSD}_{\text{perm}} = \min_{\pi \in S_{N_{\text{equiv}}}} \text{RMSD}(\mathbf{R}, \pi(\mathbf{R}')) \tag{S87}$$

where $\pi(\mathbf{R}')$ denotes the structure with fragments reordered according to permutation π. To bound the cost, the number of enumerated permutations is capped (default 10,000); if a system would exceed this, the comparison falls back to the standard (non-permuted) RMSD.

S5.4.2 Implementation

Algorithm S19 Permutation-Invariant RMSD

Require: Structures $\mathbf{R}$, $\mathbf{R}'$, fragment assignments, equivalence classes
Ensure: Minimum RMSD over permutations

```
Identify equivalent fragment groups
Generate all permutations of equivalent fragments
RMSD_min ← ∞
for each permutation π do
    R'_π ← reorder atoms according to π
    d ← RMSD(R, R'_π) with Kabsch alignment
    RMSD_min ← min(RMSD_min, d)
end for
return RMSD_min
```

This ensures that fragment reorientation is not counted as conformational change during ensemble pruning.

S5.5 NCI Workflow Integration

NCI handling is integrated throughout the conformational sampling workflow.

S5.5.1 Activation

NCI mode is activated with the `--nci` flag; multi-fragment inputs ($N_F > 1$) without it trigger a warning.

S5.5.2 Modified Sampling

When NCI mode is active:

1. **Potential modification**: Confinement energy is added during MTD/MD:

$$E_{\text{total}} = E_{\text{potential}} + E_{\text{bias}} + E_{\text{conf}} \tag{S88}$$

2. **Optimization cycles**: NCI distance filtering is applied at each optimization cycle with adaptive thresholds (Table S7).

3. **RMSD pruning**: Permutation-invariant RMSD is used when equivalent fragments are present.

S5.5.3 Workflow Checkpoints

NCI filtering is applied at four stages:

1. After Phase 1 (MTD grid)
2. After Phase 2 (conformer refinement)
3. After Phase 3 (rotamer MD)
4. After Phase 4 (genetic crossing)

S5.6 Configuration Parameters

Table S8: NCI module parameters

Parameter	Description	Default
`--nci`	Enable multi-fragment confinement + filtering	off
`--nci-buffer`	Distance from outermost atom to confinement wall	3.0 Å
`--nci-mode`	Confinement type (`wall`/`harmonic`)	`wall`
`--nci-max-dist`	Base inter-fragment contact threshold, $d_{\text{base}} = \max(d_{\text{init}}, \cdot)$	3.0 Å

The confinement force constant ($k = 10$ eV) and wall exponent ($n = 12$) are fixed internal defaults of the confinement potential and are not exposed as command-line options.

S6 Machine-Learned Potentials

The ML potential module provides a registry-based system for integrating machine-learning potentials. New potentials can be added by subclassing the base wrapper class and registering with a decorator.

S6.1 Architecture Overview

S6.1.1 Registry System

Potentials are registered using a decorator pattern:

```
@register_potential("my_potential")
class MyPotential(PotentialWrapper):
    ...
```

Registered potentials are instantiated via the factory function:

```
potential = get_potential("my_potential", species=atomic_numbers, **kwargs)
```

S6.1.2 PotentialWrapper Base Class

All potentials inherit from the abstract base class `PotentialWrapper`, which defines the interface:

S6.2 Energy Function Requirements

The energy function returned by `_build_energy_fn()` must satisfy:

Table S9: PotentialWrapper interface methods

Method	Required	Description
`_build_energy_fn()`	Yes	Return JAX-differentiable energy function
`initialize(positions)`	No	One-time setup (e.g., allocate neighbor lists)
`build_energy_fn(with_charges)`	No	Return energy function, optionally with charges
`uses_neighbor_lists`	Property	Whether potential requires neighbor lists
`allocate_neighbors(positions)`	No	Create initial neighbor list state
`update_neighbors(positions, state)`	No	Update neighbor lists for new positions
`get_neighbor_kwargs(state)`	No	Return kwargs dict for energy function
`analytical_hessian`	Property	Whether analytical Hessian is supported

S6.2.1 Signature

```
def energy_fn(positions: jnp.ndarray, **kwargs) -> float
```

- **Input**: `positions` — array of shape $(N_{\text{atoms}}, 3)$ in Å
- **Output**: Scalar energy in eV
- **Optional kwargs**: Neighbor list indices, charges, etc.

S6.2.2 JAX Differentiability

The energy function must be compatible with JAX transformations:

- `jax.grad(energy_fn)` for forces
- `jax.hessian(energy_fn)` for vibrational analysis (or set `analytical_hessian = False` for FD fallback)
- `jax.jit(energy_fn)` for compilation
- `jax.vmap(energy_fn)` for batch evaluation

S6.2.3 Force Computation

Forces are automatically computed via automatic differentiation:

$$\mathbf{F}_i = -\frac{\partial E}{\partial \mathbf{r}_i} = -\texttt{jax.grad}(E)(\mathbf{R})_i \tag{S89}$$

No explicit force implementation is required.

S6.3 Neighbor List Interface

Potentials with pairwise interactions (cutoff-based) should implement the neighbor list interface for efficiency.

S6.3.1 JAX-MD Integration

Neighbor lists are managed using JAX-MD's `partition.neighbor_list`:

Algorithm S20 Neighbor List Setup

Require: Cutoff radius r_c, capacity multiplier c

Ensure: Neighbor list functions

```
displacement_fn, shift_fn ← space.free()
neighbor_fn ← partition.neighbor_list(
    displacement_fn, box = None, r_cutoff = r_c,
    capacity_multiplier = c, disable_cell_list = True)
return neighbor_fn
```

S6.3.2 Neighbor State Management

Three methods handle neighbor list lifecycle:

1. **Allocation**: Create initial neighbor list with sufficient capacity

```
def allocate_neighbors(self, positions):
    return self._neighbor_fn.allocate(positions, box=None)
```

2. **Update**: Recompute neighbors for new positions

```
def update_neighbors(self, positions, nbr_state):
    return nbr_state.update(positions, box=None)
```

3. **Kwargs extraction**: Convert state to energy function arguments

```
def get_neighbor_kwargs(self, nbr_state):
    return {"neighbor": nbr_state.idx}
```

S6.4 Charge Support for IR Intensities

Potentials that provide partial atomic charges enable IR intensity calculations.

S6.4.1 Charges Function

Override `build_energy_fn` to return a charges function:

```
def build_energy_fn(self, with_charges=False):
    energy_fn = self._build_energy_fn()
    if with_charges:
        def charges_fn(positions, **kwargs):
            return partial_charges  # shape (n_atoms,)
        return energy_fn, charges_fn
    return energy_fn
```

S6.4.2 Dipole Moment

The molecular dipole moment is computed from charges:

$$\boldsymbol{\mu} = \sum_i q_i \mathbf{r}_i \tag{S90}$$

IR intensities require the dipole derivative (Section S3.2).

Table S10: Available potentials in MARS

Name	Description	Neighbor Lists	Charges	Analytical Hessian
`harmonic`	Spring to centroid (test)	No	No	Yes
`lj`	Lennard-Jones pairwise	Yes	No	Yes
`so3lr`	SO3LR equivariant NN	Yes (SR+LR)	Yes	Yes
`mace`	MACE foundation model	Yes	No	Yes[†]
`dxtb`	GFN-xTB tight-binding	No	Yes	No (FD)
`confinement`	Spherical/ellipsoidal wall (NCI)	No	No	Yes

[†] Analytical Hessians for the JAX-native MACE backends (`off`/`mp`/`anicc`); the PyTorch-based `omol` backend falls back to finite differences. MACE does not currently expose partial charges, so MACE IR intensities are unavailable—use `so3lr` or `dxtb` for IR.

S6.5 Built-in Potentials

S6.5.1 SO3LR

Equivariant neural network with short-range and long-range components [8, 9]:

- Two neighbor lists: SR (4.5 Å; 5.0 Å for SO3LR-2-L) and LR (configurable via `lr_cutoff`, default 1000 Å for gas phase)
- Provides partial charges for IR intensities; accepts a total-system `charge`
- **Two SO3LR packages are supported and auto-detected** by introspecting the installed API (no version pin), so the same code runs in either environment: the *stable* `so3lr` (v0.1.x), which ships a single v1 model, and the *developing* package (v≥0.2), which adds a v2 model registry.
- Model selection via `model` (CLI `--so3lr-model`): `so3lr_v1` (legacy v1, the default) or—with the developing package—`so3lr-s`/`-m`/`-l` (v2 small/medium/large) or a filesystem path to a custom / fine-tuned model workdir. Requesting a v2 model when only the stable package is installed emits a warning and falls back to v1; the v2 models are still under active development, so v1 remains the safe default.
- Requires: `pip install so3lr` (v1) or the developing package for the v2 models

S6.5.2 MACE

Message-passing equivariant neural network [10]; the organic `off` family is the MACE-OFF23 model [11]. A *single* registry name `mace` is used; the foundation family is selected with the `foundation` argument (CLI `--mace-foundation`) and the size with `model` (`--mace-model`):

- `foundation` ∈ {`off` (organic, default), `off24` (MACE-OFF24), `mp` (materials), `anicc` (ANI-cc), `omol` (broad coverage)}
- JAX-native backend (`off`/`mp`/`anicc`) gives analytical gradients/Hessians; `omol` uses a PyTorch/ASE backend with finite-difference Hessians
- Accepts `charge` and `spin` (honored by the `omol` backend); JAX weights are cached under `cache_dir` (default `~/.cache/mars/mace_jax`)
- Requires both `pip install mace-torch` and `mace-jax` for the default JAX path

S6.5.3 GFN-xTB

Tight-binding DFT via dxtb [12]:

- Semi-empirical quantum chemistry; method selected with `method` (`gfn1`/`gfn2`)
- Provides Mulliken partial charges, so it can drive IR intensities
- Uses `jax.pure_callback` (no analytical Hessian; IR uses finite differences)
- Requires: `pip install dxtb[libcint]`

S6.6 Implementing Custom Potentials

S6.6.1 Minimal Example

A simple potential without neighbor lists:

```
from mars.potentials import PotentialWrapper, register_potential
import jax.numpy as jnp

@register_potential("morse")
class MorsePotential(PotentialWrapper):
    """Morse potential between atom pairs."""

    def __init__(self, species, D_e=1.0, alpha=1.5, r_e=1.5, **kwargs):
        self.D_e = D_e      # Well depth (eV)
        self.alpha = alpha  # Width parameter (1/Angstrom)
        self.r_e = r_e      # Equilibrium distance (Angstrom)
        self._species = jnp.array(species)

    def _build_energy_fn(self):
        D_e, alpha, r_e = self.D_e, self.alpha, self.r_e

        def energy_fn(positions, **kwargs):
            # Pairwise distances
            diff = positions[:, None, :] - positions[None, :, :]
            r = jnp.sqrt(jnp.sum(diff**2, axis=-1) + 1e-10)

            # Morse potential (upper triangle only)
            morse = D_e * (1 - jnp.exp(-alpha * (r - r_e)))**2
            mask = jnp.triu(jnp.ones_like(r), k=1)

            return jnp.sum(morse * mask)

        return energy_fn
```

S6.6.2 Example with Neighbor Lists

For potentials with cutoff:

```
@register_potential("custom_pairwise")
class CustomPairwisePotential(PotentialWrapper):

    def __init__(self, species, cutoff=6.0, **kwargs):
```

```
        self._species = jnp.array(species)
        self._cutoff = cutoff
        self._neighbor_fn = None
        self._initialized = False

    def initialize(self, positions):
        from jax_md import partition, space
        displacement_fn, _ = space.free()
        self._neighbor_fn = partition.neighbor_list(
            displacement_fn, box=None, r_cutoff=self._cutoff,
            capacity_multiplier=1.25, disable_cell_list=True
        )
        self._nbrs = self._neighbor_fn.allocate(positions)
        self._initialized = True

    def _build_energy_fn(self):
        if not self._initialized:
            raise RuntimeError("Call initialize() first")

        def energy_fn(positions, neighbor=None, **kwargs):
            # Use neighbor indices for efficient pairwise computation
            ...
            return total_energy

        return energy_fn

    @property
    def uses_neighbor_lists(self):
        return True

    def allocate_neighbors(self, positions):
        return self._neighbor_fn.allocate(positions)

    def update_neighbors(self, positions, nbr_state):
        return nbr_state.update(positions)

    def get_neighbor_kwargs(self, nbr_state):
        return {"neighbor": nbr_state.idx}
```

S6.6.3 Example with Charges

For IR intensity support:

```
@register_potential("charged_model")
class ChargedModelPotential(PotentialWrapper):

    def __init__(self, species, **kwargs):
        self._species = jnp.array(species)
        # ... model initialization ...

    def build_energy_fn(self, with_charges=False):
        energy_fn = self._build_energy_fn()
```

```
        if with_charges:
            def charges_fn(positions, **kwargs):
                # Compute partial charges from model
                charges = self._compute_charges(positions)
                return charges  # shape (n_atoms,)

            return energy_fn, charges_fn

        return energy_fn

    def _build_energy_fn(self):
        def energy_fn(positions, **kwargs):
            return self._model(positions)
        return energy_fn
```

S6.6.4 Integration Checklist

When implementing a new potential:

1. **Subclass** `PotentialWrapper`
2. **Register** with `@register_potential("name")`
3. **Implement** `_build_energy_fn()` returning a JAX function
4. **Ensure** output is in eV, input positions in Å
5. **Test** with `jax.grad` and `jax.jit`
6. **Optional**: Add neighbor list methods if using cutoffs
7. **Optional**: Override `build_energy_fn` for charges
8. **Optional**: Set `analytical_hessian = False` if using callbacks

S7 Graphical user interface

Every capability described in Sections S1–S6 is also reachable from a graphical interface, started with `mars gui`. The interface is a local web application: `mars gui` starts a small HTTP server on the user's own machine and opens a page in the default browser. Its purpose is to lower the entry barrier of the command line, structure preparation, option discovery, queueing and the inspection of ensembles, trajectories and spectra, without creating a second, divergent way of running MARS. Figure S3 shows the four pages of the interface.

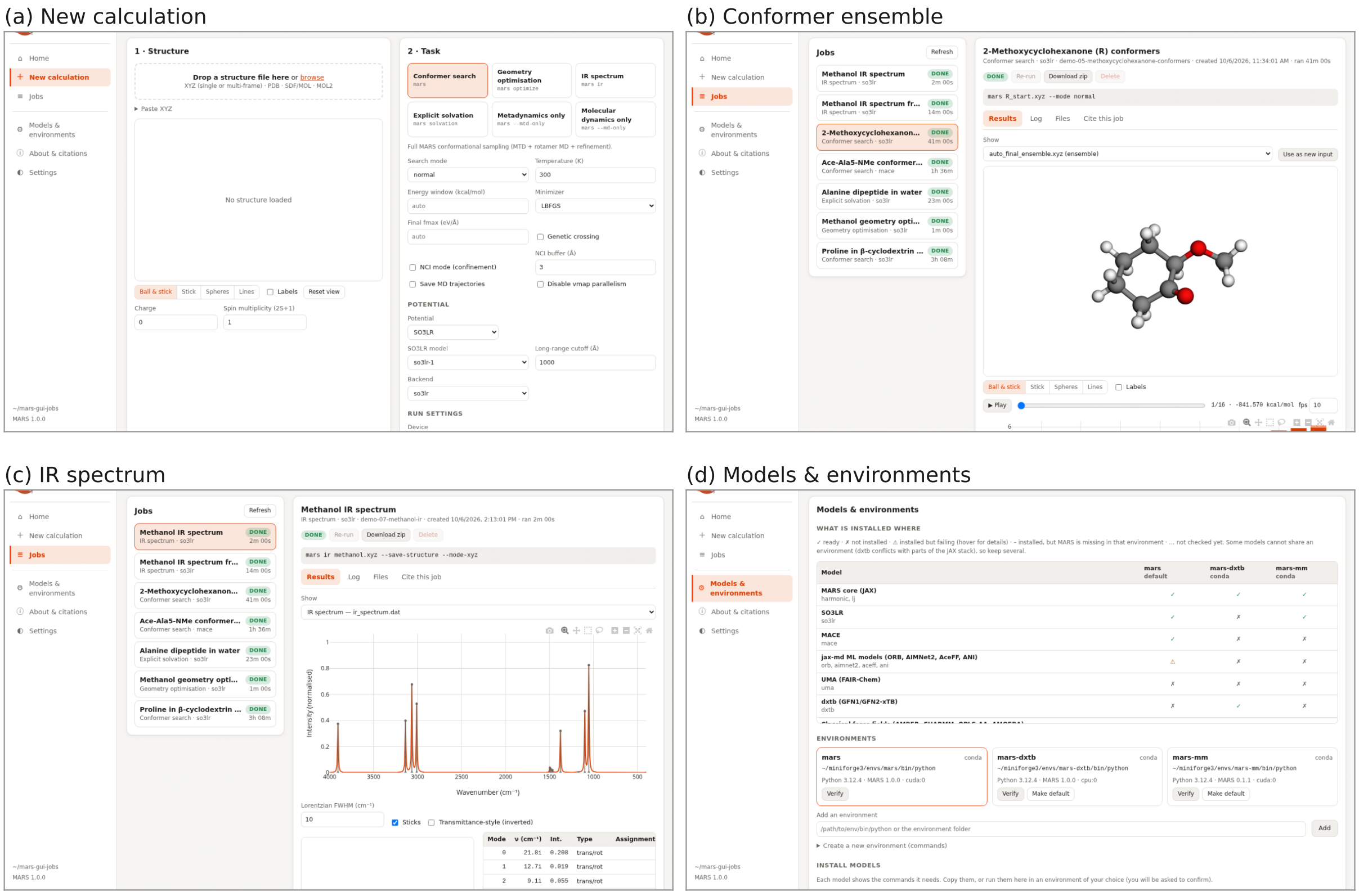

Figure S3: The MARS graphical interface (`mars gui`). **(a)** *New calculation*: the structure is dropped or pasted in and previewed in 3D (left), and the task, its options, the potential and the run settings are chosen on the right. **(b)** A finished conformer search: the ensemble is played back frame by frame with its relative energies, and any member can be sent back to *New calculation* as the input of the next job. **(c)** An IR job: the broadened spectrum with the stick spectrum, the adjustable Lorentzian width, and the mode table with the automatic assignments of Section S3. **(d)** *Models & environments*: which model families are installed in which Python environment, with the per-environment checks and the installation commands. The screenshots are taken from `mars gui -demo`, which opens the interface preloaded with finished example jobs; the banner this mode displays at the top of every page has been cropped.

The interface is a front end to the command-line interface and not a reimplementation of it. A job configured in the browser is translated into a `mars` command, which is displayed before the run starts, written to `command.sh` in the job directory, and stored in the job record. The run itself is that command, executed as an ordinary subprocess. Every calculation set up graphically can be repeated verbatim in a command-line.Six tasks are offered, each mapping to one command-line entry point: conformer search (`mars`), geometry optimisation (`mars optimize`), IR spectrum (`mars ir`), explicit solvation (`mars solvation`), metadynamics only (`mars --mtd-only`) and plain molecular dynamics (`mars --md-only`).

A finished job is presented through four views. *Results* renders what the job produced: final structures and conformer ensembles in a 3D viewer with frame-by-frame playback and the relative-energy profile, solvation clusters, trajectories, and IR spectra with an adjustable Lorentzian width, the stick spectrum, the mode table with the automatic assignments of Section S3, and animated normal modes. *Log* streams the run log while the job is running. *Files* lists every output file, downloadable individually or as a single archive of the job directory. *Cite this job* collects the references of the methods and models that particular job actually used.

Two operations connect jobs. *Re-run* clones a job identically. *Use as new input* sends a structure from the results of one job (a selected conformer, an optimised geometry, a solvated cluster..) into the input of the next (conformer search, then optimisation of a selected minimum, then its IR spectrum).

The models MARS supports cannot all be installed together: dxtb conflicts with parts of the JAX stack, and the PyTorch-based potentials pull in their own pinned dependencies. In practice a user therefore keeps several Python environments, which is a frequent source of confusion when a model appears to be missing. The *Models & environments* page makes that state explicit. It shows a matrix of model families against the environments the interface knows—its own, every `conda` environment, and any interpreter added by path—marking each entry as ready, not installed, installed but failing (with the error, typically absent model weights), or installed without MARS itself. Each environment reports its Python and MARS versions, can be verified on demand (which imports every model and lists the GPUs that JAX detects), and can be made the default used when a model is available in more than one. For each model family the exact installation commands are shown; they can be copied and run by the user, or executed in a chosen environment from the interface after a confirmation dialogue that displays every command that will be run. Steps that cannot be automated, such as accepting a model licence or authenticating against a model repository, are listed rather than attempted. When a job is submitted, the interface marks the environments that provide the selected potential and selects one of them.

`mars gui --demo` opens the interface populated with finished example jobs built from the outputs of the case studies deposited in the Zenodo archive of this work, an IR spectrum, conformer ensembles obtained with SO3LR and with MACE, a host–guest complex treated in NCI mode, and a solvated peptide, together with fictional environments. In this mode nothing is computed, and nothing is installed, so the interface, the result views and the environment page can be examined on a machine on which no potential, and no GPU, is available. The example jobs are distributed with the package (`examples/gui_demo`).

S8 Implementation validation

MARS reaches the potentials of Section S6 by several routes: SO3LR through MARS's own JAX-MD neighbour-list interface, the MACE-OFF models through a conversion to JAX, dxtb through a JAX callback, and MACE-OMol through a PyTorch bridge. This section establishes that each route reproduces the reference implementation of the same model. Part A compares single-point energies and forces; Part B compares molecular dynamics.

S8.1 Part A: single-point energies and forces

Every conformer of the conformer-energy benchmark set of Section S9 and Table 1 of the main text (36 molecules, 14–116 atoms, 454 conformers) was evaluated with MARS's potential layer and with the reference implementation of the same model: the ASE calculator distributed with each machine-learned potential (`So3lrCalculator` and `mace-torch`), and, for GFN1- and GFN2-xTB, the xtb 6.5.1 binary. Both sides use the same model weights and, for SO3LR, the same long-range cutoff (1000 Å). Each machine-learned potential was evaluated in single (fp32) and double (fp64) precision, and the GFN Hamiltonians in double precision.

Two implementations may differ in absolute energy by a constant arising from their isolated-atom references, which is immaterial to every property MARS computes. Energy agreement is therefore measured on relative energies, as the range of $E_{MARS} - E_{\text{ref}}$ over the conformers of a molecule (ΔE_{rel}), which does not depend on the choice of reference conformer. Force agreement is measured as the largest absolute difference over all Cartesian components of all conformers ($\Delta F_{\max}$), and as the worst per-conformer agreement in force direction, $1 - \cos\theta$. For the single-conformer molecule, 2,6-difluorobenzaldehyde, ΔE_{rel} vanishes by construction and only the forces are tested.

Each pair is held to a tolerance appropriate to what it compares. *Port* pairs compare MARS's JAX evaluation with the reference implementation of the model and constitute the actual test; they must satisfy $\Delta E_{\text{rel}} \leq 10^{-4}$ kcal/mol and $\Delta F_{\max} \leq 10^{-4}$ eV/Å in fp64. *Interface* pairs run the identical implementation on both sides and must agree to 10^{-8}; they verify the wiring and validate no physics. MACE-OMol belongs to this class: its non-linear interaction blocks cannot yet be converted to JAX, so MARS evaluates the PyTorch model through `jax.pure_callback` and agreement with ASE is exact by construction. *Cross-code* pairs compare independently written implementations of the same Hamiltonian, which need not agree to 10^{-4}, and are held to 10^{-2}. The port criterion is defined for fp64; single precision is treated separately below.

Table S11: Double-precision (fp64) single-point agreement between MARS and the ASE calculator of each machine-learned potential over the 36 molecules (454 conformers) of the curated benchmark set. ΔE_{rel}: largest range of $E_{\text{MARS}} - E_{\text{ASE}}$ over the conformers of a molecule (kcal/mol); $\Delta F_{\max}$: largest absolute force-component difference (eV/Å); $1 - \cos\theta$: worst per-conformer force-direction agreement. All values are maxima over the 36 molecules; the last column counts molecules within tolerance.

Model	Class	ΔE_{rel}	$\Delta F_{\max}$	$1 - \cos\theta$	within tol.
SO3LR	port	7.8×10^{-6}	7.8×10^{-7}	1.2×10^{-8}	36/36
SO3LR-2-S	port	3.5×10^{-5}	2.6×10^{-6}	3.3×10^{-11}	36/36
SO3LR-2-M	port	8.3×10^{-6}	1.5×10^{-6}	9.2×10^{-12}	36/36
SO3LR-2-L	port	6.6×10^{-6}	9.4×10^{-7}	3.9×10^{-12}	36/36
MACE-OFF23 S	port	9.4×10^{-5}	8.0×10^{-6}	3.9×10^{-10}	36/36
MACE-OFF23 M	port	6.3×10^{-5}	2.8×10^{-6}	6.3×10^{-11}	36/36
MACE-OFF23 L	port	4.2×10^{-5}	3.3×10^{-6}	6.1×10^{-11}	36/36
MACE-OFF24	port	6.3×10^{-5}	2.7×10^{-6}	3.2×10^{-11}	36/36
MACE-OMol	interface	0	4.9×10^{-15}	5.6×10^{-16}	36/36

All eight port pairs are within tolerance on every molecule in fp64 (Table S11). Relative energies agree to 9.4×10^{-5} kcal/mol or better and forces to 8.0×10^{-6} eV/Å or better, with worst-conformer force directions identical to $1-\cos\theta \leq 1.2\times10^{-8}$. MACE-OMol agrees to machine precision because both sides evaluate the same PyTorch model.

Single precision. Each machine-learned potential was also evaluated in fp32, and each implementation's fp32 result is compared with its own fp64 result in Table S12, so that the effect of the precision can be separated from any difference between the implementations. Forces are insensitive to the precision in every model and in both implementations ($\Delta F_{\max} \leq 8.9\times10^{-4}$ eV/Å). Relative energies are insensitive for SO3LR ($\leq 4.6\times10^{-3}$ kcal/mol in both implementations) but not for the MACE models: the largest error over the 36 molecules is 1.0–1.5 kcal/mol in MARS and 1.1–1.9 kcal/mol in the reference implementation, and the median molecule is off by 0.10–0.17 kcal/mol in MARS against 0.022 kcal/mol with `mace-torch`. The total energies of these models are of order 10^4 eV (median 14 814 eV), because the isolated-atom reference energies are included in the total, and single precision resolves a number of that size only to $\approx 10^{-3}$ eV (0.022 kcal/mol), which equals the median error of the reference implementation. SO3LR totals are of order 10^2 eV, two orders of magnitude smaller, and show no such effect. The comparison of the two fp32 evaluations directly gives 4.2×10^{-3} kcal/mol at most for SO3LR and 1.4–2.3 kcal/mol for the MACE-OFF models, and for MACE-OMol, where both sides run the identical PyTorch model, it is still 2.2 kcal/mol; forces of the two fp32 evaluations agree to 3.0×10^{-5} eV/Å in all models. The energy error of single-precision MACE evaluations is therefore a property of the precision, present in both implementations and in the identical-model comparison, rather than of the port. The typical error is larger in MARS than in `mace-torch` (median 0.10–0.17 against 0.022 kcal/mol), while the worst cases are comparable. Forces, and hence optimisation, molecular dynamics and vibrational spectra, are unaffected, but relative conformer energies from MACE models should be computed in double precision (`--float64`), as done for the benchmarks reported here.

Table S12: Effect of single precision on single-point energies and forces of each machine-learned potential, for MARS (JAX) and for the reference implementation (ASE calculators), over the 36 molecules of the curated benchmark set. In each implementation the fp32 result is compared with the fp64 result of the same implementation. ΔE_{rel}: range of $E_{\mathrm{fp32}} - E_{\mathrm{fp64}}$ over the conformers of a molecule (kcal/mol), given as the maximum and the median over molecules; $\Delta F_{\max}$: largest absolute force-component difference (eV/Å). $|E_{\mathrm{tot}}|$: median absolute total energy (eV). For MACE-OMol both columns use the same PyTorch model.

		MARS (JAX)			ASE		
Model	$\|E_{\mathrm{tot}}\|$	$\Delta E_{\mathrm{rel}}^{\max}$	$\Delta E_{\mathrm{rel}}^{\mathrm{med}}$	$\Delta F_{\max}$	$\Delta E_{\mathrm{rel}}^{\max}$	$\Delta E_{\mathrm{rel}}^{\mathrm{med}}$	$\Delta F_{\max}$
SO3LR	42	1.2×10^{-3}	3.8×10^{-4}	4.1×10^{-4}	1.2×10^{-3}	2.7×10^{-4}	4.1×10^{-4}
SO3LR-2-S	112	3.2×10^{-3}	7.7×10^{-4}	8.9×10^{-4}	3.8×10^{-3}	9.1×10^{-4}	8.9×10^{-4}
SO3LR-2-M	112	3.0×10^{-3}	7.0×10^{-4}	5.3×10^{-4}	3.2×10^{-3}	8.4×10^{-4}	5.2×10^{-4}
SO3LR-2-L	112	3.2×10^{-3}	7.9×10^{-4}	4.1×10^{-4}	4.6×10^{-3}	7.7×10^{-4}	4.1×10^{-4}
MACE-OFF23 S	14 814	1.07	0.17	4.1×10^{-4}	1.30	0.022	4.1×10^{-4}
MACE-OFF23 M	14 814	1.34	0.17	4.1×10^{-4}	1.94	0.022	4.1×10^{-4}
MACE-OFF23 L	14 814	0.96	0.10	4.1×10^{-4}	1.06	0.022	4.1×10^{-4}
MACE-OFF24	14 814	1.50	0.13	4.1×10^{-4}	1.34	0.023	4.0×10^{-4}
MACE-OMol	14 806	1.16	0.022	4.1×10^{-4}	1.31	0.022	4.1×10^{-4}

Table S13: Single-point agreement between MARS (dxtb) and the xtb 6.5.1 binary for the GFN Hamiltonians in fp64 over the 36 molecules (454 conformers) of the curated benchmark set; columns as in Table S11, with the cross-code tolerance of 10^{-2}. "analytic" uses the gradient printed by xtb; "symmetry-safe" uses the energy at the reference geometry and the average of the analytic forces at two geometries displaced by $\pm\boldsymbol{\delta}$ ($|\boldsymbol{\delta}| \sim 10^{-4}$ Å), which cancels the linear term of the displacement (see text).

Hamiltonian	xtb forces	ΔE_{rel}	$\Delta F_{\max}$	$1 - \cos\theta$	within tol.
GFN1	xtb analytic	3.5×10^{-4}	0.88	0.85	29/36
GFN1	xtb symmetry-safe	3.5×10^{-4}	3.8×10^{-5}	1.1×10^{-9}	36/36
GFN2	xtb analytic	6.7×10^{-4}	0.86	0.99	29/36
GFN2	xtb symmetry-safe	6.4×10^{-4}	3.5×10^{-5}	1.3×10^{-9}	36/36

For the GFN Hamiltonians (Table S13), MARS and the xtb binary agree on relative energies to within 6.4×10^{-4} kcal/mol on all 36 molecules. The analytic forces of the xtb 6.5.1 binary agree with MARS to better than 10^{-2} eV/Å on 29 molecules, but on seven (n-butane, n-hexane, n-heptane, n-octane, isohexane, 3-methylpentane and FGG) they differ from MARS by 0.05–0.9 eV/Å in a few Cartesian components of the affected conformers, while the energies of the two codes agree on those same conformers to 3×10^{-6} kcal/mol. The discrepancy lies in xtb: finite differences of xtb's own energy at the reference geometries of n-butane, n-hexane and n-octane reproduce the MARS forces to 7×10^{-6} eV/Å and differ from the xtb analytic gradient by 0.52, 0.53 and 0.88 eV/Å. The error occurs only on exactly symmetric geometries: the affected conformers of n-butane, n-hexane and n-octane are centrosymmetric (every coordinate about the centroid occurs in an exact $\pm$ pair), and displacing the atoms by 10^{-4} Å restores the agreement of the analytic gradient with finite differences to 10^{-5} eV/Å. Conformers with only partial symmetry show smaller errors ($\sim 7 \times 10^{-3}$ eV/Å for neoheptane, isopentane and isoheptane). A reference free of this artefact is obtained by averaging the analytic forces at two geometries displaced by $\pm\boldsymbol{\delta}$, which cancels the term linear in $\boldsymbol{\delta}$ and leaves an error of order $|\boldsymbol{\delta}|^2 \approx 10^{-8}$ Å^2 times the third derivative; the energy is taken at the undisplaced geometry. Against this symmetry-safe reference MARS agrees to 3.8×10^{-5} and 3.5×10^{-5} eV/Å (GFN1 and GFN2) on all 36 molecules, with force directions identical to $1-\cos\theta \leq 1.3\times10^{-9}$. On six test geometries the symmetry-safe forces themselves differ from MARS by 4×10^{-6}–2.4×10^{-5} eV/Å.

S8.2 Part B: molecular dynamics

The same potentials were compared in molecular dynamics on Ala_2 (22 atoms), cyclophosphamide (29), GFA (40) and CHPSAR (70), starting from the first reference conformer. Each machine-learned potential was run through MARS and through its ASE calculator on the same NVIDIA V100 GPU, in double precision, with an identical protocol: 300 K, a 0.5 fs time step, ten replicas, 50 ps of NVT with a Nosé–Hoover chain thermostat followed by 5 ps of NVE, repeated five times. MARS integrates the ten replicas together in one batched kernel, whereas ASE propagates them one after the other. The cost is reported per step, atom and replica, which removes the differences in system size and replica count; the NVE energy drift is the slope of a linear fit to the total energy per atom. One hundred snapshots of each MARS trajectory were re-evaluated with the ASE calculator to test the agreement along the dynamics.

Table S14: Molecular dynamics through MARS and through the ASE calculator of each potential (fp64, one V100, four molecules of 22–70 atoms). Cost in μs per step, atom and replica (range over the four molecules); speed-up = ASE/MARS cost (range, geometric mean in parentheses); largest absolute NVE drift over the four molecules (meV/atom/ps); largest force difference between MARS and ASE on 100 snapshots per molecule (ΔF_{max}, eV/Å).

	Cost (μs)			NVE drift		
Model	MARS	ASE	Speed-up	MARS	ASE	ΔF_{max}
SO3LR-1	14–15	124–355	8.5–23 (15)	4.9×10^{-4}	4.6×10^{-4}	6.8×10^{-7}
SO3LR-2-S	9.4–11	109–308	11–28 (19)	9.6×10^{-4}	8.4×10^{-4}	2.4×10^{-6}
SO3LR-2-M	14–15	129–348	8.9–22 (15)	6.5×10^{-4}	3.9×10^{-4}	1.5×10^{-6}
SO3LR-2-L	28–34	145–387	4.3–14 (8.4)	4.6×10^{-4}	4.8×10^{-4}	1.1×10^{-6}
MACE-OFF23 S	20–41	706–5523	17–281 (84)	5.2×10^{-4}	8.9×10^{-4}	6.9×10^{-6}
MACE-OFF23 M	52–138	852–6025	6.2–115 (31)	3.9×10^{-4}	5.3×10^{-4}	2.4×10^{-6}
MACE-OFF23 L	205–624	1334–6047	2.1–29 (8.5)	8.6×10^{-4}	5.8×10^{-4}	3.2×10^{-6}
MACE-OFF24	53–137	862–5696	6.3–108 (30)	4.0×10^{-4}	5.5×10^{-4}	3.2×10^{-6}
MACE-OMol	1220–2675	1640–7620	1.3–2.8 (2.0)	7.6×10^{-4}	1.2×10^{-3}	8.0×10^{-15}

MARS is faster than the ASE route for every potential (Table S14): by a factor of 8–19 (geometric mean) for the SO3LR models and 8–84 for the MACE-OFF models. The gain is largest for the smallest molecule and decreases with size (SO3LR-1: 23× for Ala_2, 8.5× for CHPSAR), because the per-step overhead of the ASE loop dominates for small systems while the batched MARS kernel keeps the GPU occupied. MACE-OMol, which runs the identical PyTorch model on both sides, gains only 1.3–2.8×, from the batching of the replicas. Fitted over the four molecules (22–70 atoms), the cost per step grows as $N^{0.94}$ for SO3LR-1 in MARS, the linear behaviour expected for a local potential, but is nearly independent of size in ASE ($N^{0.09}$), which is therefore latency-bound at these sizes. The NVE drifts of the two routes are of the same size, at most 1.2×10^{-3} meV/atom/ps, and along the trajectories the two implementations agree to 6.9×10^{-6} eV/Å in the forces and 4×10^{-5} meV/atom in the energies; for MACE-OMol they are identical to machine precision.

For the GFN Hamiltonians, dxtb in MARS (2 ps NVT with two replicas) was compared with the xtb binary (10 ps, one trajectory, 1–28 OpenMP threads) on the same 28-core CPU node (Table S15). The xtb binary gains a factor of 2–4 from multithreading, saturating at 8–28 threads, and its cost per step grows as $N^{1.7}$–$N^{1.8}$ over the four molecules (fastest thread count). MARS+dxtb costs 9.7–14.2 ms (GFN1) and 23.9–31.7 ms (GFN2) per step, atom and replica, 15–28 and 27–89 times more than xtb at its best thread count (0.32–0.96 ms), because dxtb is evaluated through a PyTorch callback that JAX executes replica by replica. Its NVE drift is at most 3.6×10^{-4} meV/atom/ps. The dxtb route is therefore suited to single points, optimisations and Hessians within MARS workflows, while molecular dynamics with GFN-xTB is better run with the xtb binary.

Table S15: Molecular dynamics with the GFN Hamiltonians: MARS+dxtb (2 ps NVT, two replicas) against the xtb 6.5.1 binary (10 ps NVT, one trajectory) with 1–28 OpenMP threads, both on the same 28-core CPU node. Cost in ms per step, atom and replica; the fastest xtb setting is in bold; the last column is the MARS+dxtb cost divided by the fastest xtb cost.

			xtb, OpenMP threads						
Molecule	Atoms	MARS+dxtb	1	2	4	8	16	28	Ratio
GFN1									
Ala_2	22	10.6	1.27	0.80	0.47	0.46	0.39	**0.38**	28
Cyclophosphamide	29	13.8	1.88	1.15	0.90	0.71	**0.62**	0.62	22
GFA	40	9.69	2.04	1.28	0.90	0.64	**0.61**	0.61	16
CHPSAR	70	14.2	2.79	1.76	1.47	1.17	1.01	**0.96**	15
GFN2									
Ala_2	22	28.2	0.55	0.45	0.33	**0.32**	0.35	0.36	89
Cyclophosphamide	29	31.7	1.13	0.86	**0.61**	0.65	0.66	0.75	52
GFA	40	24.5	1.14	0.89	0.73	**0.58**	0.64	0.70	43
CHPSAR	70	23.9	1.87	1.45	0.99	0.91	0.91	**0.90**	27

S9 Conformational benchmark

The curated high-level conformational-search set (summarised in Table 1 of the main text) comprises 36 molecules spanning 454 conformers across seven chemical classes:

1. **Linear, branched alkanes:** Eleven molecules (n-butane through n-octane, the iso/neo isomers, and 3-methylpentane) referenced to CCSD(T)/cc-pVTD or B2K-PLYP-D(0.22)/pc-2//PW6B95/6-311G(d,p) values reported by Gruzman, Karton, and Martin [13].

2. **Cyclohexane:** Four ring-pucker conformers (chair/twist-boat family) with reference energies from Kang and Park [14] at the CCSD(T)/CBS-aTQ limit. One of these, the half-chair ($4H_3$), is a first-order saddle point on the ring-inversion pathway rather than a local minimum. It is consequently excluded from the optimisation/retention analysis (cyclohexane is there assessed over its three minima: the $^4C_1$ chair, the twist-boat, and the boat) but retained in the single-point evaluations, where a fixed-geometry energy on the saddle point is well defined.

3. **Halogenated benzaldehydes:** The four isomers 2,3-, 2,4-, 2,5- and 2,6-difluorobenzaldehyde probe the aldehyde rotational barrier under varying ortho-/meta-fluorination patterns and are sensitive to electrostatic anisotropy and intramolecular C–H$\cdots$F contacts. Reference DLPNO–CCSD(T)/def2-TZVP energies were taken from [15].

4. **Free amino acids:** Aspartic acid, cysteine, serine, and tryptophan exhibit several closely-spaced conformers stabilised by intramolecular hydrogen bonds and side-chain rotation; reference energies are drawn from [16] and serve as a calibration point for the larger peptide subset.

5. **Alanine dipeptide:** Ala–Ala (Ala_2) provides a prototypical peptide conformational landscape governed by backbone ϕ/ψ torsions. The benchmark includes multiple minima across the Ramachandran map, with reference MP2/aug-cc-pVQZ energies reported in [17].

6. **MPCONF196 drug-like molecules:** Thirteen pharmaceutically relevant systems as a curated set of 196 high-quality conformers whose reference is CCSD(T)/CBS and DLPNO-CCSD(T)/CBS method [18]. In some of the systems, reference optimised structures were obtained at DFT level combined with implicit solvation; consequently, some conformers may not correspond to true gas-phase minima.

7. **Phosphamide chemotherapeutics:** Cyclophosphamide and isophosphamide are clinically used pro-drugs that are stereoelectronically distinctive (P=O, N-CH_2CH_2Cl arms) and absent from standard conformer benchmarks. Reference energies and geometries were extracted from [19].

Table S16: Relative conformer-energy MAE (RMSE) per dataset compared with higher-level literature references. Per-dataset literature references — Alkane [13], Cyclohexane [14], DFB [15], Amino acids [16], Alanine dipeptide [17], MPCONF196 [18], Phosphamides [19]. All values in kcal/mol.

Method	Alkanes	Cyhx	DFB	AA[b]	Ala2	MPCONF196	Phos.
n	183	4	7	41	6	196	12
MACE-OFF23 S	0.14 (0.19)	0.40 (0.49)	0.11 (0.19)	0.48 (0.64)	0.70 (0.84)	1.46 (1.98)	0.79 (1.04)
MACE-OFF23 M	0.11 (0.15)	0.40 (0.48)	0.12 (0.19)	0.70 (0.91)	0.30 (0.38)	0.96 (1.30)	0.74 (0.92)
MACE-OFF24	0.12 (0.17)	0.50 (0.59)	0.22 (0.35)	0.76 (0.98)	0.37 (0.44)	0.77 (1.12)	0.95 (1.21)
MACE-OMol	0.16 (0.20)	0.05 (0.07)	0.20 (0.31)	0.83 (0.98)	0.22 (0.32)	0.58 (0.90)	0.51 (0.66)
SO3LR-1	0.33 (0.43)	1.11 (1.36)	0.11 (0.19)	1.47 (1.75)	0.42 (0.47)	1.98 (2.65)	2.03 (2.30)
SO3LR-2-S	0.29 (0.37)	1.13 (1.35)	0.12 (0.23)	1.31 (1.53)	0.43 (0.48)	1.37 (1.79)	0.87 (1.13)
SO3LR-2-M	0.81 (0.93)	0.77 (0.90)	0.23 (0.36)	1.36 (1.57)	0.32 (0.48)	1.36 (1.88)	0.89 (1.12)
SO3LR-2-L	0.58 (0.68)	0.31 (0.39)	0.33 (0.52)	1.36 (1.59)	0.32 (0.45)	1.01 (1.46)	0.59 (0.73)
dxtb GFN1	1.03 (1.24)	2.12 (2.50)	0.41 (0.66)	0.74 (1.01)	0.56 (0.68)	2.61 (3.58)	1.16 (1.53)
dxtb GFN2	0.30 (0.40)	0.49 (0.57)	0.13 (0.21)	0.79 (1.03)	0.71 (0.88)	2.11 (2.98)	1.30 (1.57)
PBE0+MBD	0.85 (1.02)	0.13 (0.16)	0.26 (0.39)	1.64 (1.92)	0.40 (0.50)	1.96 (3.14)	0.74 (1.09)
ωB97M-V	0.18 (0.22)	0.04 (0.06)	0.20 (0.31)	0.84 (1.00)	0.20 (0.30)	0.65 (1.10)	0.56 (0.75)

[b]Amino acids comprise 46 conformers in total; the literature reference [16] covers 41 (five cysteine conformers lack a published value), so the literature-referenced statistics here use $n = 41$, whereas the computed-DFT tables above use all 46.

Table S17: Relative conformer-energy MAE (RMSE) per dataset compared with ωB97M-V/def2-TZVPD as the reference. Per-dataset conformers references — Alkane [13], Cyclohexane [14], DFB [15], Amino acids [16], Alanine dipeptide [17], MPCONF196 [18], Phosphamides [19]. All values in kcal/mol.

Method	Alkanes	Cyhx	DFB	AA	Ala2	MPCONF196	Phos.
n	183	4	7	46	6	196	12
MACE-OFF23 S	0.16 (0.20)	0.36 (0.44)	0.09 (0.16)	0.99 (1.16)	0.67 (0.79)	1.38 (2.00)	0.51 (0.73)
MACE-OFF23 M	0.14 (0.17)	0.35 (0.44)	0.08 (0.12)	0.30 (0.38)	0.15 (0.24)	0.74 (1.00)	0.27 (0.38)
MACE-OFF24	0.15 (0.20)	0.45 (0.56)	0.03 (0.06)	0.27 (0.35)	0.27 (0.34)	0.79 (1.22)	0.43 (0.52)
MACE-OMol	0.03 (0.03)	0.01 (0.01)	0.01 (0.02)	0.05 (0.07)	0.03 (0.04)	0.27 (0.42)	0.10 (0.13)
SO3LR-1	0.48 (0.59)	1.07 (1.30)	0.31 (0.47)	0.73 (0.91)	0.29 (0.38)	2.07 (2.80)	1.57 (1.79)
SO3LR-2-S	0.19 (0.26)	1.09 (1.30)	0.10 (0.20)	0.68 (0.83)	0.30 (0.40)	1.39 (1.78)	0.59 (0.81)
SO3LR-2-M	0.64 (0.73)	0.72 (0.86)	0.03 (0.07)	0.52 (0.63)	0.22 (0.31)	1.28 (1.70)	0.70 (0.91)
SO3LR-2-L	0.42 (0.48)	0.27 (0.35)	0.14 (0.23)	0.52 (0.62)	0.12 (0.18)	0.97 (1.36)	0.59 (0.73)
dxtb GFN1	0.87 (1.03)	2.07 (2.44)	0.61 (0.94)	1.38 (1.52)	0.60 (0.70)	2.41 (3.32)	0.89 (1.01)
dxtb GFN2	0.24 (0.33)	0.45 (0.52)	0.21 (0.36)	1.11 (1.32)	0.57 (0.73)	2.06 (2.93)	0.88 (1.05)
PBE0+MBD	0.68 (0.81)	0.18 (0.21)	0.06 (0.09)	0.88 (1.00)	0.33 (0.39)	1.59 (2.34)	0.22 (0.37)

Table S18: Relative conformer-energy MAE (RMSE) per dataset compared with PBE0+MBD@rsSCS as the reference. Per-dataset conformers references — Alkane [13], Cyclohexane [14], DFB [15], Amino acids [16], Alanine dipeptide [17], MPCONF196 [18], Phosphamides [19]. All values in kcal/mol.

Method	Alkanes	Cyhx	DFB	AA	Ala2	MPCONF196	Phos.
n	183	4	7	46	6	196	12
MACE-OFF23 S	0.81 (0.96)	0.54 (0.63)	0.15 (0.24)	1.81 (2.08)	0.91 (1.12)	2.34 (3.55)	0.61 (0.79)
MACE-OFF23 M	0.81 (0.96)	0.53 (0.63)	0.13 (0.21)	1.03 (1.21)	0.38 (0.50)	1.89 (2.77)	0.39 (0.57)
MACE-OFF24	0.82 (0.98)	0.63 (0.74)	0.04 (0.08)	0.96 (1.13)	0.51 (0.63)	2.01 (3.05)	0.31 (0.39)
MACE-OMol	0.70 (0.84)	0.18 (0.21)	0.05 (0.08)	0.90 (1.03)	0.35 (0.41)	1.76 (2.62)	0.28 (0.46)
SO3LR-1	1.16 (1.37)	1.24 (1.50)	0.36 (0.56)	0.67 (0.82)	0.36 (0.56)	2.74 (3.82)	1.42 (1.69)
SO3LR-2-S	0.65 (0.82)	1.27 (1.49)	0.16 (0.28)	0.84 (1.05)	0.37 (0.50)	2.08 (2.93)	0.70 (0.88)
SO3LR-2-M	0.21 (0.28)	0.90 (1.05)	0.03 (0.06)	0.51 (0.64)	0.49 (0.65)	2.18 (3.16)	0.85 (1.06)
SO3LR-2-L	0.32 (0.41)	0.45 (0.53)	0.08 (0.15)	0.43 (0.54)	0.31 (0.37)	1.97 (2.93)	0.74 (0.99)
dxtb GFN1	0.41 (0.51)	2.25 (2.64)	0.67 (1.03)	2.17 (2.36)	0.66 (0.85)	2.95 (4.33)	0.76 (0.86)
dxtb GFN2	0.73 (0.94)	0.62 (0.72)	0.27 (0.44)	1.80 (2.05)	0.41 (0.50)	2.98 (4.30)	0.74 (0.93)
ωB97M-V	0.68 (0.81)	0.18 (0.21)	0.06 (0.09)	0.88 (1.00)	0.33 (0.39)	1.59 (2.34)	0.22 (0.37)

Table S19: Sampling recovery per method and subset: mean recovery (%) with, in parentheses, the total number of recovered over available low-energy reference conformers summed over the molecules of the subset (main text Figure 3C).

Method	Alkanes	Cyclohexane	DFB	Amino acids	Ala2	MPCONF196	Phosphamides
MACE-OFF23 S	56 (88/183)	50 (2/4)	75 (6/7)	85 (39/46)	100 (6/6)	19 (18/89)	42 (5/12)
MACE-OFF23 M	57 (88/183)	50 (2/4)	62 (5/7)	98 (45/46)	83 (5/6)	19 (21/90)	75 (9/12)
MACE-OFF24	43 (81/183)	25 (1/4)	100 (7/7)	85 (39/46)	83 (5/6)	23 (23/88)	50 (2/3)
MACE-OMol	55 (79/183)	33 (1/3)	75 (6/7)	85 (40/46)	83 (5/6)	36 (27/91)	33 (4/12)
SO3LR-1	63 (99/183)	50 (2/4)	75 (6/7)	88 (41/46)	100 (6/6)	30 (25/91)	33 (4/12)
SO3LR-2-S	58 (82/183)	100 (4/4)	75 (6/7)	88 (41/46)	100 (6/6)	35 (29/90)	42 (5/12)
SO3LR-2-M	55 (90/183)	33 (1/3)	100 (7/7)	92 (43/46)	100 (6/6)	37 (28/92)	50 (6/12)
SO3LR-2-L	50 (84/183)	67 (2/3)	100 (7/7)	91 (42/46)	100 (6/6)	26 (23/94)	67 (8/12)
CREST	58 (107/183)	25 (1/4)	100 (7/7)	98 (45/46)	83 (5/6)	51 (48/90)	50 (6/12)

Table S20: TorsionNet500 [20] relative conformer-energy errors (kcal/mol) for the potentials available in MARS, against two independent references evaluated on the same 500 molecules / 12 000 conformers. MAE and RMSE use per-molecule mean-centred relative energies; $\mathrm{MAE}_{\mathrm{min}}$ references each profile to its lowest conformer.

	ωB97M-V/def2-TZVPPD [11]			PBE0+MBD [8]		
Method	MAE	RMSE	$\mathrm{MAE}_{\mathrm{min}}$	MAE	RMSE	$\mathrm{MAE}_{\mathrm{min}}$
MACE-OFF23 S	0.26	0.36	0.33	0.38	0.50	0.52
MACE-OFF23 M	0.15	0.20	0.19	0.33	0.44	0.46
MACE-OFF24	0.19	0.25	0.25	0.36	0.48	0.50
MACE-OMol	0.05	0.07	0.07	0.29	0.38	0.40
SO3LR-1	0.79	1.08	1.18	0.72	0.97	1.04
SO3LR-2-S	0.58	0.81	0.87	0.59	0.80	0.82
SO3LR-2-M	0.38	0.54	0.51	0.45	0.61	0.61
SO3LR-2-L	0.27	0.36	0.36	0.36	0.50	0.50
dxtb GFN1	0.69	0.93	0.89	0.62	0.87	0.83
dxtb GFN2	0.54	0.73	0.69	0.50	0.68	0.66

Table S21: CCCBDB [21] torsion errors (kcal/mol) for the potentials available in MARS, against experiment and against the matching PBE0+MBD scans, over the 37 molecules common to all potentials. The experiment columns are min-referenced per profile; the PBE0+MBD columns are mean-centred per profile. The last row is the PBE0+MBD-vs-experiment baseline on the same molecules.

	vs experiment		vs PBE0+MBD	
Method	MAE	RMSE	MAE	RMSE
MACE-OFF23 S	0.36	0.78	0.31	0.80
MACE-OFF23 M	0.37	0.84	0.15	0.38
MACE-OFF24	0.32	0.58	0.21	0.55
MACE-OMol	0.39	0.98	0.08	0.20
SO3LR-1	0.53	1.26	0.35	0.95
SO3LR-2-S	0.37	0.77	0.23	0.45
SO3LR-2-M	0.38	0.84	0.15	0.31
SO3LR-2-L	0.36	0.83	0.10	0.28
dxtb GFN1	0.73	1.15	0.55	0.89
dxtb GFN2	0.71	1.09	0.45	0.71
PBE0+MBD (ref.)	0.46	1.28	—	—

Table S22: QUID [22] dimer interaction-energy errors (kcal/mol) for the potentials available in MARS, against CCSD(T) (primary) and PBE0+MBD (secondary), over 42 complexes. ME is the signed mean error (predicted − reference).

	vs CCSD(T)			vs PBE0+MBD	
Method	MAE	RMSE	ME	MAE	RMSE
MACE-OFF23 S	0.78	1.07	+0.51	0.89	1.17
MACE-OFF23 M	0.47	0.61	+0.28	0.57	0.72
MACE-OFF24	0.54	0.71	+0.21	0.64	0.79
MACE-OMol	0.34	0.40	−0.18	0.61	0.71
SO3LR-1	2.67	3.07	+2.64	2.54	2.91
SO3LR-2-S	2.27	2.75	+2.27	2.08	2.65
SO3LR-2-M	1.18	1.53	+1.12	1.03	1.48
SO3LR-2-L	0.78	1.07	+0.43	0.85	1.10
dxtb GFN1	2.13	2.39	+2.13	1.93	2.29
dxtb GFN2	0.88	1.12	+0.62	1.02	1.27

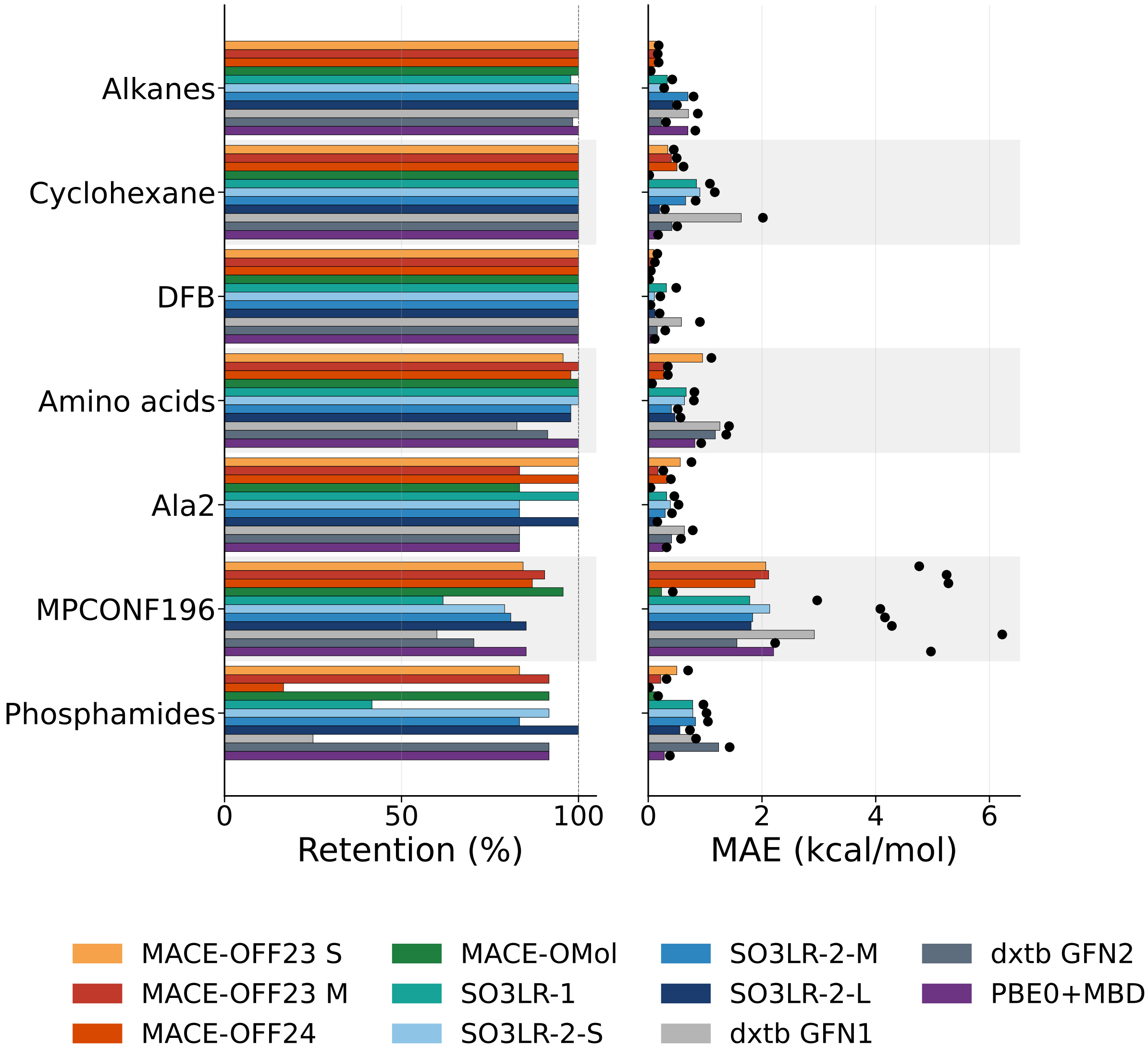

Figure S4: Minimum retention (left, fraction of reference conformers within 0.3 Å heavy-atom Kabsch RMSD after unconstrained optimisation) and retained-set relative-energy MAE (bars) / RMSE (dots) (right), per subset and per potential, using the ωB97M-V/def2-TZVPD reoptimized geometries and energies as the reference. PBE0+MBD appears as a comparator row; the ωB97M-V self-comparison is omitted.

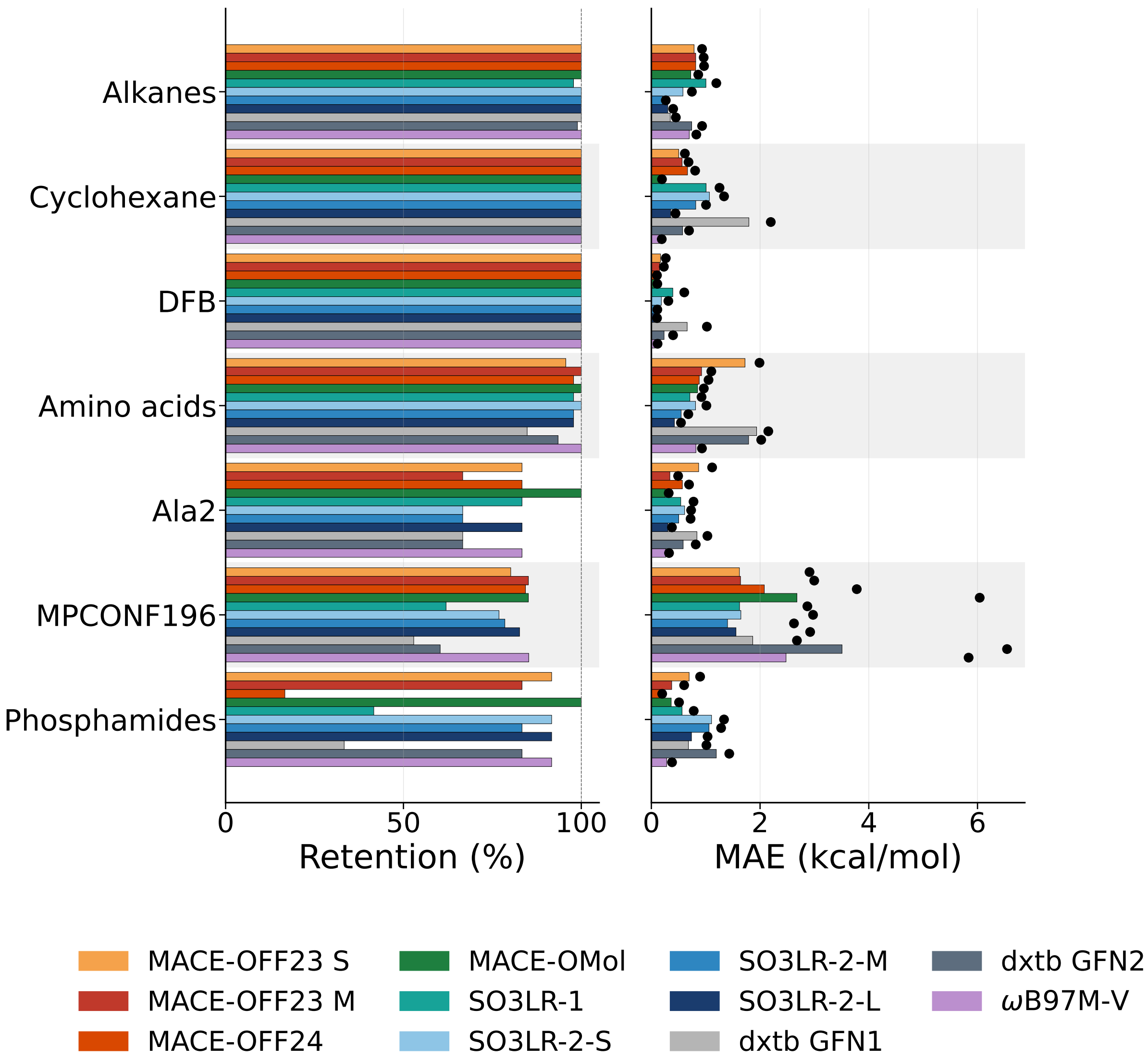

Figure S5: As Figure S4, but using the PBE0+MBD@rsSCS reoptimized geometries and energies as the reference. ωB97M-V appears as a comparator row; the PBE0+MBD self-comparison is omitted.

S10 Reproducibility Test

We quantify the run-to-run reproducibility of MARS conformational sampling under both single- (fp32) and double-precision floating-point arithmetic for three GPU-accelerated MLFF families: SO3LR (four models — the legacy SO3LR-1 and the SO3LR-2 *small*, *medium*, *large* variants), MACE-OFF23 (*small* and *medium*), MACE-OFF24, and MACE-OMol. MARS uses fixed pseudorandom seeds, so all replica-to-replica variation observed is due to the non-deterministic ordering of GPU reductions, which isolates the precision effect. Across five molecules spanning 14 to 70 atoms, all four SO3LR variants converge in every one of the 400 replicas and reach the lowest-energy structure to within $\sigma_{E_{\min}} \lesssim 3 \times 10^{-3}$ kcal/mol at both precisions on the small, rigid systems. MACE-OMol converges in all 100 replicas. The MACE-OFF models at single precision are operationally unusable: only 13/50 of the MACE-OFF23 *small*, 18/50 of the MACE-OFF23 *medium* and 21/50 of the MACE-OFF24 replicas produce a final ensemble, while at fp64 all 150 MACE-OFF replicas converge and reach the same lowest-energy structure with $\sigma_{E_{\min}} \leq 2 \times 10^{-3}$ kcal/mol in 12 of the 15 cells (the other three, on GFA and CHPSAR, lie at 0.14–0.34 kcal/mol) — 13 to 6500 times (median 3100 times) smaller than at fp32 in the cells where both are defined.

S10.1 Methods

S10.1.1 System set

Five molecules span 14 to 70 atoms and a range of flexibility: n-butane (14 atoms), the alanine dipeptide Ala_2 (22), cyclophosphamide (29, containing P and Cl), the GFA tripeptide of MPCONF196 (40), and CHPSAR of MPCONF196 (70). For each molecule we used the reference geometry from the `conformers_benchmark` test set as the single starting structure.

S10.1.2 Sampling protocol

All sampling was performed with the default `mars` conformer-search subcommand in "normal" mode: automatic configuration based on molecular flexibility drove a 12-step multi-step MTD grid over 60 ps followed by three diverse refinement starts. Internally, MARS calls `jax.random.PRNGKey` with a fixed seed (`random_seed=42` for the global key, with deterministic offsets per metadynamics step), so the only source of replica-to-replica variation is the non-determinism of GPU reductions.

S10.1.3 Test matrix

We ran ten independent replicas of the workflow for each of the 80 cells defined by the cross product of: 5 systems × 8 models × 2 precisions, for a total of 800 GPU jobs. Each job used one NVIDIA A100 GPU with a 48-hour wall-time limit. The fp64 case was activated through the `--float64` command-line flag; MACE variants were selected via
`--potential mace --mace-foundation {off,off24,omol}` and SO3LR variants via
`--so3lr-model {so3lr_v1,so3lr-s,so3lr-m,so3lr-l}`.

S10.1.4 Reproducibility metrics

For every (system, model, precision) cell we report:

- n_{ok} — number of replicas (out of ten) that produced a valid final ensemble (`auto_final_ensemble.xyz`). Non-convergent replicas finish the workflow with the diagnostic "*all structures generated by Phase 1 MTD were discarded (broken bonds or outside energy window)*" and write no ensemble file.

- $\bar{n}_{\text{conf}} \pm \sigma_{n_{\text{conf}}}$ — mean and standard deviation across replicas of the number of unique conformers found.

- $\sigma_{E_{\text{min}}}$ — cross-replica standard deviation of the lowest energy in the final ensemble (kcal/mol). In the deterministic limit $\sigma_{E_{\text{min}}} = 0$; larger values mean replicas relaxed to different minima or the same minimum along different numerical paths. Reported only for cells with $n_{\text{ok}} \geq 2$.

S10.2 Results

S10.2.1 Convergence rate

Figure S6 and Table S23 show how many replicas of each cell completed successfully. Three well-defined patterns emerge:

1. All four SO3LR variants converge in every replica at both precisions across all five systems (400/400 converged runs). The precision choice does not affect whether SO3LR completes.

2. MACE-OMol converges in every one of its 100 replicas at both precisions across the five systems.

3. MACE-OFF23 and MACE-OFF24 fail catastrophically at fp32. The MACE-OFF23 *small* variant converges in 13/50 fp32 replicas; the four largest systems produce zero to three converged replicas each. The *medium* variant is similar (18/50 fp32 converged); GFA and CHPSAR yield zero converged fp32 replicas. MACE-OFF24 behaves the same way, with 21/50 fp32 replicas converged (butane 10, Ala_2 2, cyclophosphamide 9, GFA 0, CHPSAR 0). The log of every one of its 29 failed replicas contains the Phase 1 diagnostic described above and no traceback, and none of the jobs ended in a time-out. Double precision restores every MACE-OFF23 and MACE-OFF24 cell to 10/10, including the peptide systems (Ala_2, GFA, CHPSAR).

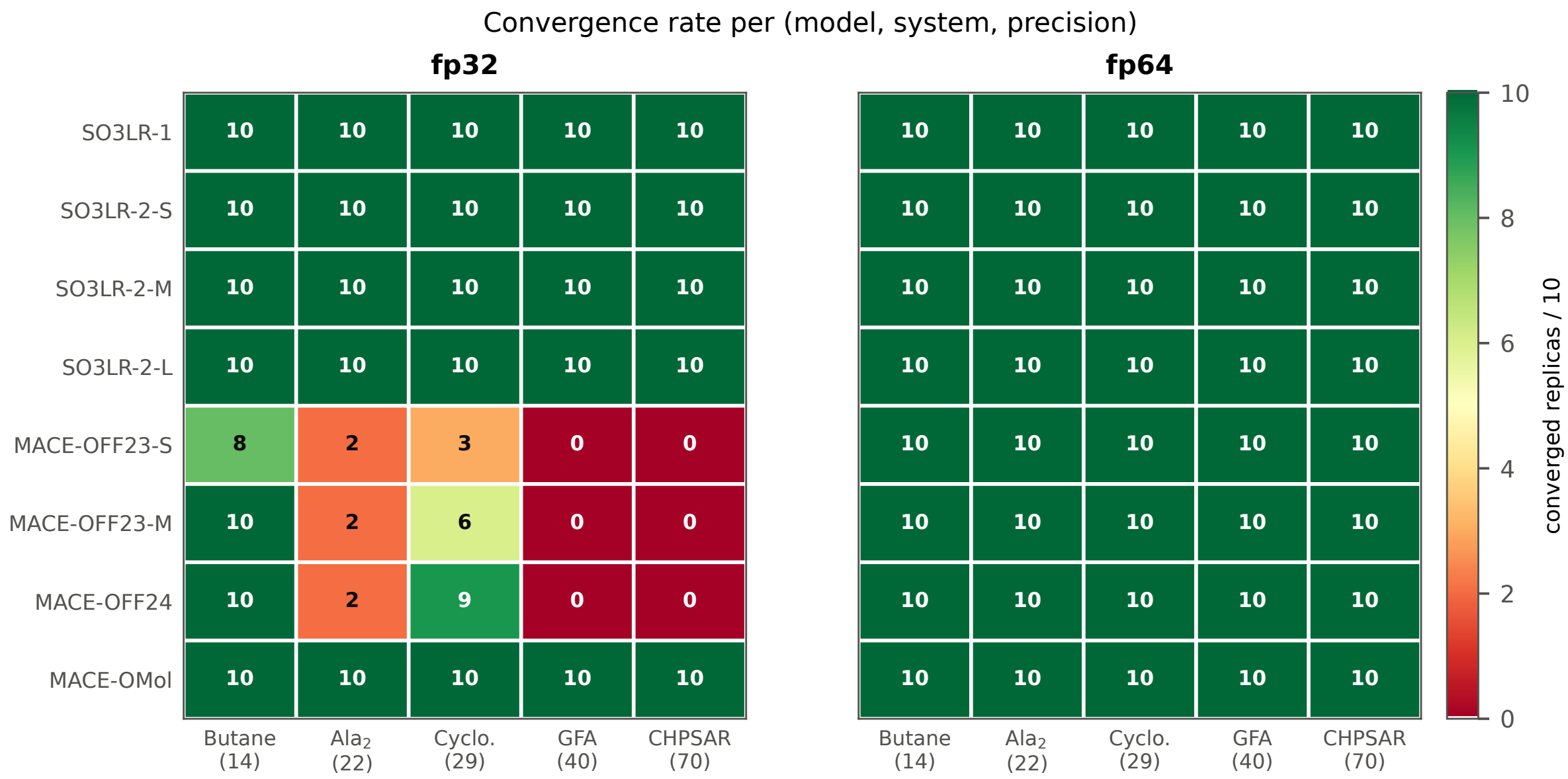

Figure S6: Convergence rate per (model, system, precision). Each cell gives the number of replicas (out of ten) that produced a final ensemble. All four SO3LR variants achieve 10/10 everywhere. MACE-OFF23 is unusable at fp32 for four of the five systems but returns to 10/10 at fp64. MACE-OMol is fully converged at both precisions.

Table S23: Number of converged replicas out of ten per cell. Bold marks ≤ 5 converged replicas.

	Butane		Ala_2		Cyclo.		GFA		CHPSAR	
Model	32	64	32	64	32	64	32	64	32	64
SO3LR-1	10	10	10	10	10	10	10	10	10	10
SO3LR-2-S	10	10	10	10	10	10	10	10	10	10
SO3LR-2-M	10	10	10	10	10	10	10	10	10	10
SO3LR-2-L	10	10	10	10	10	10	10	10	10	10
MACE-OFF23-S	8	10	**2**	10	**3**	10	**0**	10	**0**	10
MACE-OFF23-M	10	10	**2**	10	6	10	**0**	10	**0**	10
MACE-OFF24	10	10	**2**	10	9	10	**0**	10	**0**	10
MACE-OMol	10	10	10	10	10	10	10	10	10	10

S10.2.2 Reproducibility of the lowest-energy structure

Figures S7, S8 and Table S24 report the cross-replica standard deviation of the lowest energy $\sigma_{E_{\min}}$ for every cell with at least two converged replicas. All four SO3LR variants stay below 10^{-2} kcal/mol on the flat/compact systems (butane, Ala_2, GFA) at both precisions, indicating essentially deterministic behaviour. On the more flexible systems (cyclophosphamide, CHPSAR) the SO3LR family shows larger spreads (up to $\sim$ 1.2 kcal/mol for CHPSAR/SO3LR-1/fp32) and, for most variants, fp32 and fp64 values are of the same order of magnitude — this scatter reflects the intrinsic degeneracy of the low-energy manifold sampled by MTD, not a floating-point pathology. The exceptions are SO3LR-2-L on cyclophosphamide and CHPSAR, where fp64 is 17 and 89 times *larger* than fp32 (0.083 against 0.0048 and 0.16 against 0.0018 kcal/mol), and on butane, where fp32 is 21 times larger (0.00043 against 0.00002 kcal/mol), so a precision effect cannot be excluded there, although it does not act in a consistent direction. For MACE-OMol, $\sigma_{E_{\min}}$ is exactly zero at fp32 on the four smaller systems (all ten replicas reach the identical lowest energy), but on CHPSAR it is 0.25 kcal/mol at fp32 against 0.0022 kcal/mol at fp64.

MACE-OFF23 and MACE-OFF24 tell a much sharper story: at fp32, $\sigma_{E_{\min}}$ reaches 1.0 kcal/mol on Ala_2/MACE-OFF23-M, 3.2 kcal/mol on cyclophosphamide/MACE-OFF23-S and 1.65 kcal/mol on cyclophosphamide/MACE-OFF24, and is undefined on GFA/CHPSAR because zero fp32 replicas converged. At fp64 the same cells collapse to $\sigma_{E_{\min}} \leq 2 \times 10^{-3}$ kcal/mol on the smaller systems and remain below 0.4 kcal/mol on the largest ones; for MACE-OFF24 on butane all ten fp64 replicas reach the identical energy. Figure S9 shows this cell-by-cell: every MACE-OFF23 and MACE-OFF24 point lies far below the diagonal (fp32 much worse), while the SO3LR and MACE-OMol points sit on it, apart from the four cells noted above.

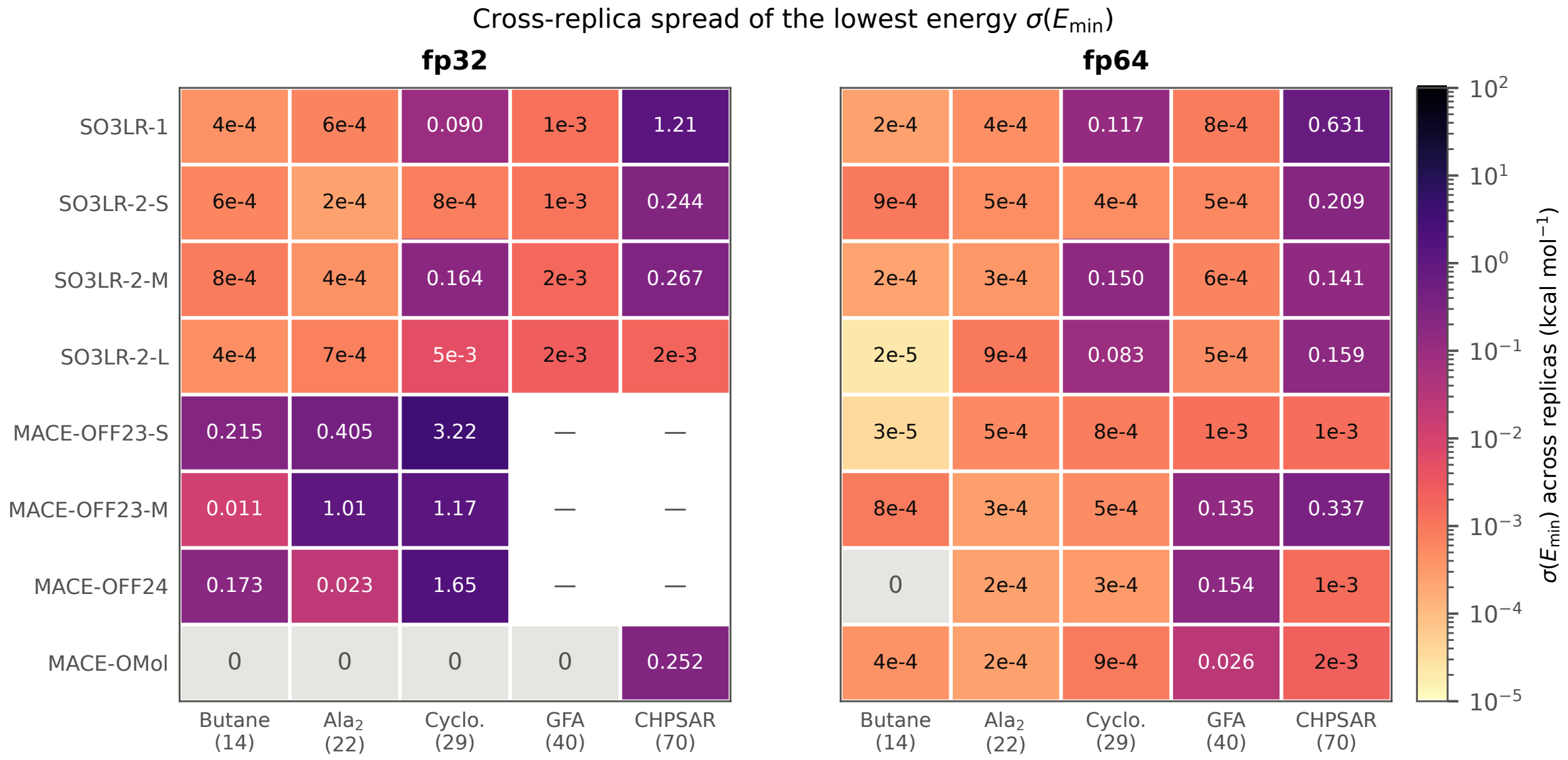

Figure S7: Cross-replica standard deviation of the lowest energy $\sigma_{E_{\min}}$ (log colour scale). Most SO3LR variants and MACE-OMol show similar values at fp32 and fp64. MACE-OFF23 and MACE-OFF24 at fp32 are either undefined (fewer than two replicas converged) or one to four orders of magnitude noisier than at fp64. Cells with $\sigma_{E_{\min}} = 0$ (identical replicas) are shown in grey and cannot be placed on the logarithmic scale.

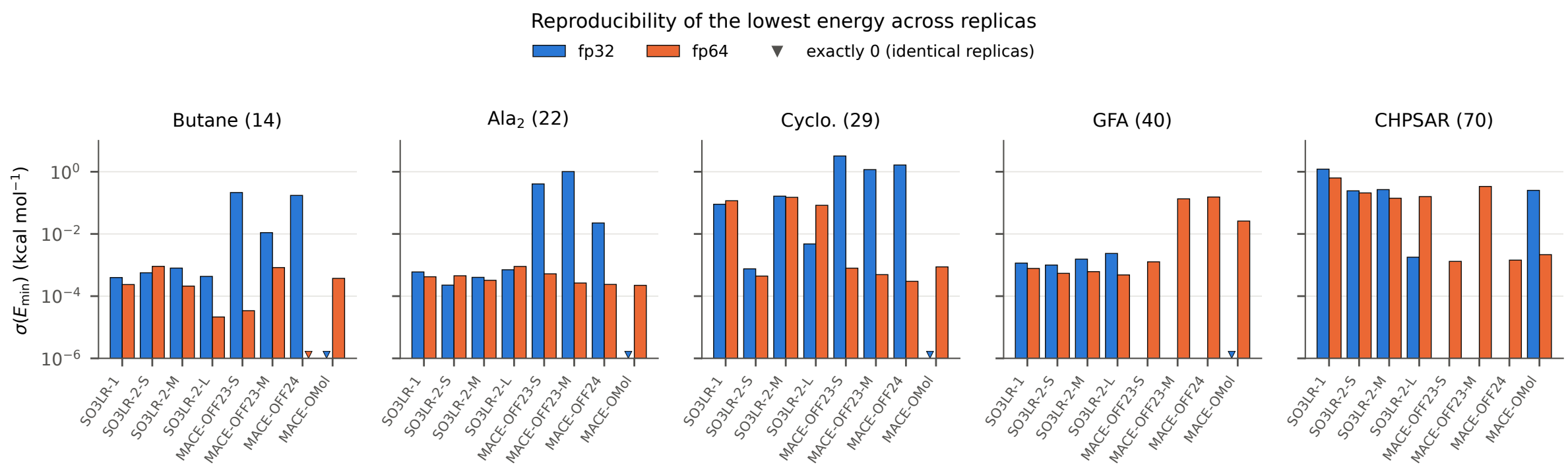

Figure S8: Same information as Figure S7 as grouped bars per system, log scale. Missing bars correspond to cells with fewer than two converged replicas; triangles mark cells in which all replicas gave the identical lowest energy.

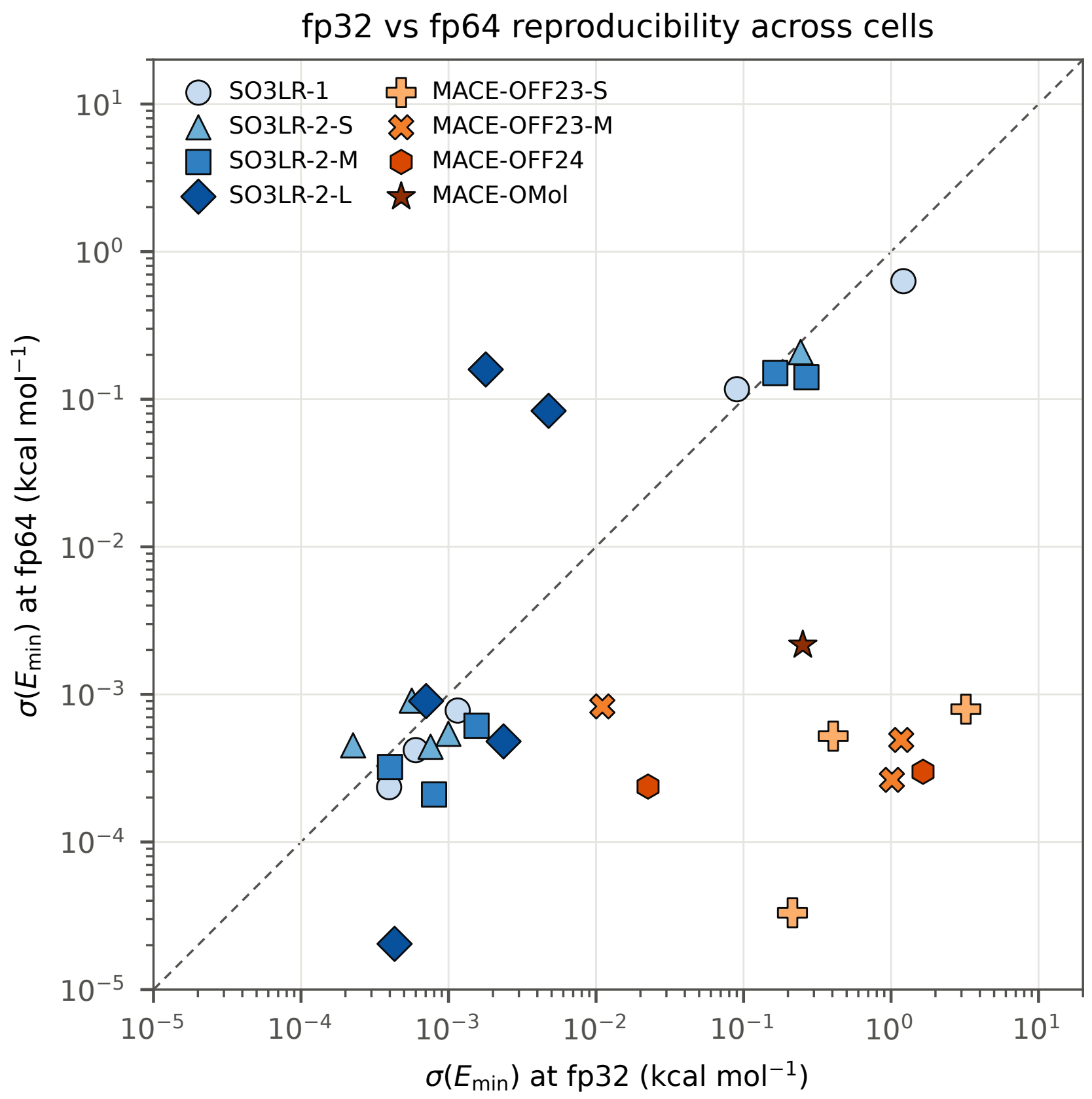

Figure S9: Cell-by-cell fp32 vs fp64 reproducibility. Points on the diagonal indicate identical reproducibility at either precision (most SO3LR variants and MACE-OMol cells); points below the diagonal indicate better reproducibility at fp64 (MACE-OFF23-S/M and MACE-OFF24, by one to four orders of magnitude, and MACE-OMol on CHPSAR); SO3LR-2-L lies above the diagonal on cyclophosphamide and CHPSAR.

Table S24: Cross-replica standard deviation of the lowest energy $\sigma_{E_{\mathrm{min}}}$ (kcal/mol). "–" = fewer than two converged replicas.

	Butane		Ala_2		Cyclo.		GFA		CHPSAR	
Model	32	64	32	64	32	64	32	64	32	64
SO3LR-1	0.0004	0.0002	0.0006	0.0004	0.090	0.117	0.0012	0.0008	1.21	0.631
SO3LR-2-S	0.0006	0.0009	0.0002	0.0005	0.0008	0.0004	0.0010	0.0005	0.244	0.209
SO3LR-2-M	0.0008	0.0002	0.0004	0.0003	0.164	0.150	0.0015	0.0006	0.267	0.141
SO3LR-2-L	0.0004	0.0000	0.0007	0.0009	0.0048	0.083	0.0024	0.0005	0.0018	0.159
MACE-OFF23-S	0.215	0.0000	0.405	0.0005	3.22	0.0008	–	0.0013	–	0.0013
MACE-OFF23-M	0.011	0.0008	1.01	0.0003	1.17	0.0005	–	0.135	–	0.337
MACE-OFF24	0.173	0.0000	0.023	0.0002	1.65	0.0003	–	0.154	–	0.0014
MACE-OMol	0.0000	0.0004	0.0000	0.0002	0.0000	0.0009	0.0000	0.026	0.252	0.0022

S10.2.3 Spread in the number of conformers found

A complementary view is how consistently each cell finds the same *number* of conformers across replicas (Figure S10). For SO3LR and MACE-OMol on small systems the count is fully reproducible or varies by at most one to two conformers. On the flexible/large systems the count distribution widens for every model but stays symmetric with respect to precision. The patho-

logical entries again live in the MACE-OFF23 and MACE-OFF24 fp32 columns, where converged replicas of the four larger systems typically return a single conformer rather than the tens to hundreds that fp64 or SO3LR produce.

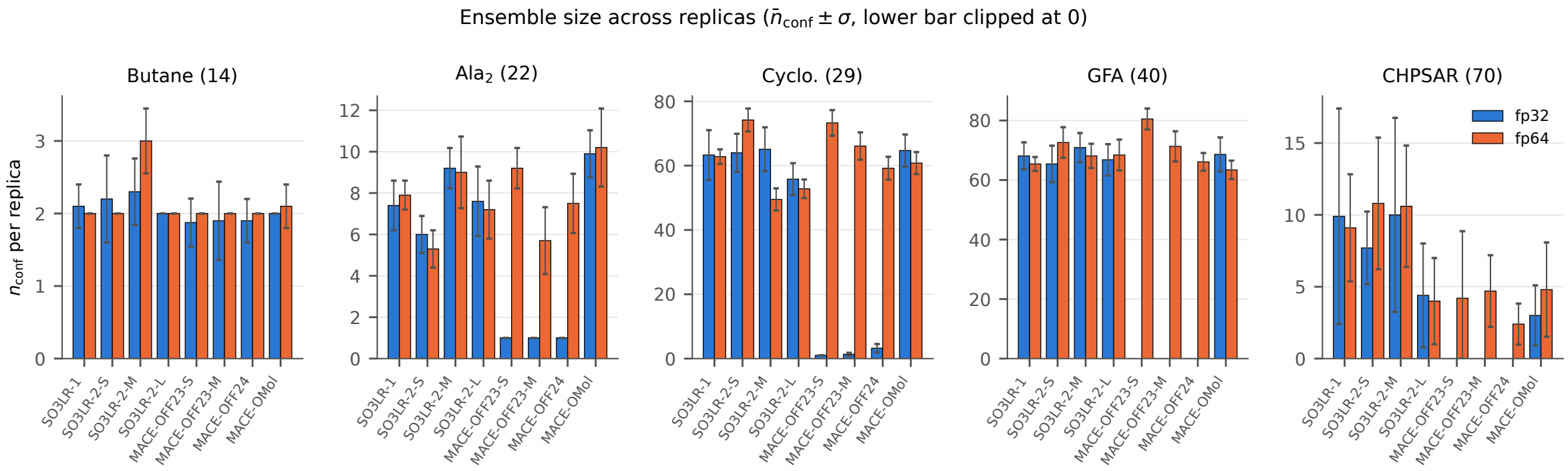

Figure S10: Number of conformers per replica across the eight models, grouped by system. Error bars are the standard deviation over the ten replicas of each cell (the lower bar is clipped at zero). MACE-OFF23 and MACE-OFF24 fp32 (blue) return few conformers for Ala_2/cyclophosphamide/GFA/CHPSAR, while fp64 recovers the SO3LR-like counts.

S10.3 Discussion

Three observations are robust across the eight models and five systems:

1. **Where both precisions converge, the lowest energies agree for SO3LR on the compact systems but not otherwise.** For SO3LR the mean lowest energies at the two precisions agree to within 10^{-3} kcal/mol in 11 of 20 cells and differ by more than 10^{-2} kcal/mol only on cyclophosphamide and CHPSAR (seven cells, up to 0.98 kcal/mol), where $\sigma_{E_{\min}}$ is itself of that size. For MACE-OMol the five cells differ by a median of 0.12 kcal/mol (up to 0.52). For MACE-OFF23 and MACE-OFF24 the nine cells with both precisions converged differ by a median of 0.96 kcal/mol (up to 5.2), and rest on only 2–10 converged fp32 replicas. The precision effect is thus predominantly one of operational stability for SO3LR, but for MACE-OFF it affects the result itself.

2. **MACE-OFF23 fp32 fails in the post-MTD relaxation optimizer, not in the MD.** The initial geometry optimization and metadynamics trajectory both complete; the subsequent optimizer step that relaxes MTD snapshots breaks bonds in ∼20% of snapshots at fp32. A topology pruner discards the broken structures, and after the energy-window cut zero or one structures survive to enter refinement.

3. **SO3LR (all variants) is robust.** The SO3LR-2 variants (*small*, *medium*, *large*) show no operational failures and $\sigma_{E_{\min}}$ symmetric with respect to precision on the flexible systems — the scatter reflects the irreducible degeneracy of the low-energy manifold, not a floating-point pathology.

S10.4 Recommendation

Based on these 800 GPU replicas covering eight MLFF variants and two floating-point precisions, we recommend for production conformational sampling: **MACE-OFF23 and MACE-OFF24 — double precision required**; **SO3LR (all four variants) — fp32 is safe**; **MACE-OMol — fp32 acceptable, fp64 preferred** when ensemble-level reproducibility matters.

S11 Non-covalent benchmark

This section provides the full data underlying the non-covalent sampling benchmark of the main text. The test set [23, 24] comprises 20 biologically relevant hydrogen-bonded dimers spanning neutral and ionic O/N/C–H donors and halogen contacts; for each dimer a conformational ensemble was generated with SO3LR-1, giving 406 conformers in total. Relative conformer energies, ΔE, are referenced to the lowest-energy conformer of each dimer; the three single-conformer dimers do not contribute a relative energy, so $n = 403$ conformers enter every comparison below. Each machine-learned force field is benchmarked against the density functional it is trained against — the SO3LR family (SO3LR-1 and SO3LR-2 S/M/L) against PBE0+MBD, and the MACE family (MACE-OFF23 small/medium, MACE-OFF24, and MACE-OMol) against ωB97M-V/def2-TZVPD — and the functional-to-functional agreement (PBE0+MBD vs ωB97M-V, evaluated on the same geometries) is reported as the irreducible baseline.

S11.1 Dataset

The 20 dimers are drawn from three NCI-ATLAS subsets [23, 24]: the neutral hydrogen-bond set HB375×10 (O–H, N–H and weak C–H donors), the σ-hole/halogen and related contacts HB300SPX×10, and the ionic hydrogen-bond set IHB100×10 (charged donors/acceptors). They span 8–27 atoms and total charges from −2 to +1, with reference interaction energies $E_{\text{int}}^{\text{ref}}$ ranging from −7 to −40 kcal/mol. Table S25 lists, for each system, its subset, interaction motif, total charge, number of atoms, the number of SO3LR-1 conformers entering the benchmark, and the reference interaction energy. Motif codes denote the donor–acceptor pair (e.g. OH–O, NH–N, CH–O; XH–O/N for σ-hole contacts; the "a"/"k" suffixes mark anionic/cationic ionic hydrogen bonds). The full set comprises 406 conformers; the three single-conformer systems do not contribute a relative energy and are therefore excluded from the ΔE statistics ($n = 403$).

Table S25: The 20 `bio_subset_20` dimers used in the non-covalent benchmark (neutral systems first, then ionic). q is the total charge, N_{at} the number of atoms, N_{conf} the number of SO3LR-1 conformers, and $E_{\text{int}}^{\text{ref}}$ the reference interaction energy (kcal/mol).

Subset	System	Motif	q	N_{at}	N_{conf}	$E_{\text{int}}^{\text{ref}}$
HB375x10	5.016	CH–O	+0	19	97	−9.0
HB375x10	5.017	CH–O	+0	18	50	−7.1
HB375x10	4.008	NH–N	+0	24	29	−9.2
HB375x10	4.023	NH–N	+0	21	64	−8.7
HB375x10	2.002	NH–O	+0	19	20	−9.7
HB375x10	2.027	NH–O	+0	23	49	−9.1
HB375x10	3.004	OH–N	+0	18	25	−14.6
HB375x10	3.012	OH–N	+0	21	21	−15.6
HB375x10	1.002	OH–O	+0	17	7	−19.3
HB375x10	1.059	OH–O	+0	16	4	−20.5
HB300SPXx10	1.21	XH–N	+0	15	1	−17.0
HB300SPXx10	2.12	XH–O	+0	11	2	−16.1
IHB100x10	08.001	NH–O (a)	−2	8	9	−26.1
IHB100x10	08.003	NH–O (a)	−1	17	7	−26.4
IHB100x10	05.003	OH–O (a)	−1	11	4	−40.3
IHB100x10	05.008	OH–O (a)	−1	20	3	−32.6
IHB100x10	03.002	NH–N (k)	+1	12	1	−31.7
IHB100x10	03.013	NH–N (k)	+1	27	1	−27.1
IHB100x10	02.002	NH–O (k)	+1	17	5	−37.0
IHB100x10	02.007	NH–O (k)	+1	22	7	−30.1
**Total (20 systems)**					**406**	

S11.2 Relative conformer energies (single point)

All force fields were evaluated as *single points* on the common SO3LR-1-generated geometries, so that the comparison isolates the quality of the potential-energy surface from any difference in the optimized structures. Table S26 lists the mean absolute error (MAE), root-mean-square error (RMSE), maximum absolute error, and Pearson correlation of ΔE against the matched DFT reference. The accuracy increases monotonically with model capacity and training-set coverage; the SO3LR-2 variants reduce the SO3LR-1 error by a factor of ∼2–3 and raise the ranking correlation from Spearman $\rho = 0.79$ (SO3LR-1) to $\rho \approx 0.95$ (SO3LR-2). MACE-OMol reaches agreement statistically indistinguishable from the DFT–DFT baseline, and both SO3LR-2-L and MACE-OFF23 medium approach it. MACE-OFF24 reaches MAE = 0.66 kcal mol^{-1} ($r = 0.933$), improving on MACE-OFF23 small (0.92 kcal mol^{-1}) and on a par with the SO3LR-2 S/M variants, but it does not match MACE-OFF23 medium, SO3LR-2-L, or MACE-OMol.

Table S26: Relative conformer-energy errors of the force fields available in MARS, evaluated as single points on the common SO3LR-1 geometries and referenced to the matched DFT level. The last row is the PBE0+MBD-vs-ωB97M-V functional-to-functional baseline. $n = 403$ conformers.

Method	Reference	n	MAE	RMSE (kcal/mol)	Max	Pearson r
SO3LR-1	PBE0+MBD	403	1.16	1.58	6.84	0.873
SO3LR-2-S	PBE0+MBD	403	0.72	1.00	6.79	0.970
SO3LR-2-M	PBE0+MBD	403	0.58	0.85	6.64	0.973
SO3LR-2-L	PBE0+MBD	403	0.43	0.73	7.05	0.977
MACE-OFF23 S	ωB97M-V	403	0.92	1.47	10.11	0.887
MACE-OFF23 M	ωB97M-V	403	0.53	0.98	5.24	0.945
MACE-OFF24	ωB97M-V	403	0.66	1.11	6.94	0.933
MACE-OMol	ωB97M-V	403	0.14	0.43	5.32	0.989
PBE0+MBD	ωB97M-V	403	0.56	0.70	5.28	0.981

S11.3 Optimized-geometry accuracy

We additionally relaxed every SO3LR-1 conformer with each force field and compared the resulting minima with the DFT-optimized structures by heavy-atom RMSD after Kabsch superposition (Figure S11, Table S27). Two matchings are reported: (i) *index-matched*, comparing each force-field minimum with the DFT minimum obtained from the *same* starting conformer; and (ii) *closest-match*, reporting for each force-field minimum the RMSD to the *nearest* DFT-optimized conformer of the same dimer. The closest-match values are systematically lower, and the gap between the two reflects conformers that relax into different — but individually valid — basins under the force field and under DFT. The 3 ωB97M-V optimizations that did not converge to the tight force criterion are excluded from the DFT-referenced comparisons. The optimized-geometry ordering mirrors the single-point energetics: SO3LR-2-L (0.18 Å index-matched to PBE0+MBD) and MACE-OMol (0.11 Å) reproduce the DFT minima essentially within the 0.11 Å DFT–DFT baseline, whereas the SO3LR-1 minima deviate by 0.77 Å. MACE-OFF24 minima are of the same quality as the MACE-OFF23 ones: relative to ωB97M-V they deviate by 0.55 Å index-matched and 0.21 Å closest-match, against 0.61/0.36 Å for MACE-OFF23 small and 0.52/0.20 Å for MACE-OFF23 medium, and thus remain well above MACE-OMol (0.12/0.05 Å). As for the single-point energetics, MACE-OFF24 therefore improves on MACE-OFF23 small but does not surpass MACE-OFF23 medium.

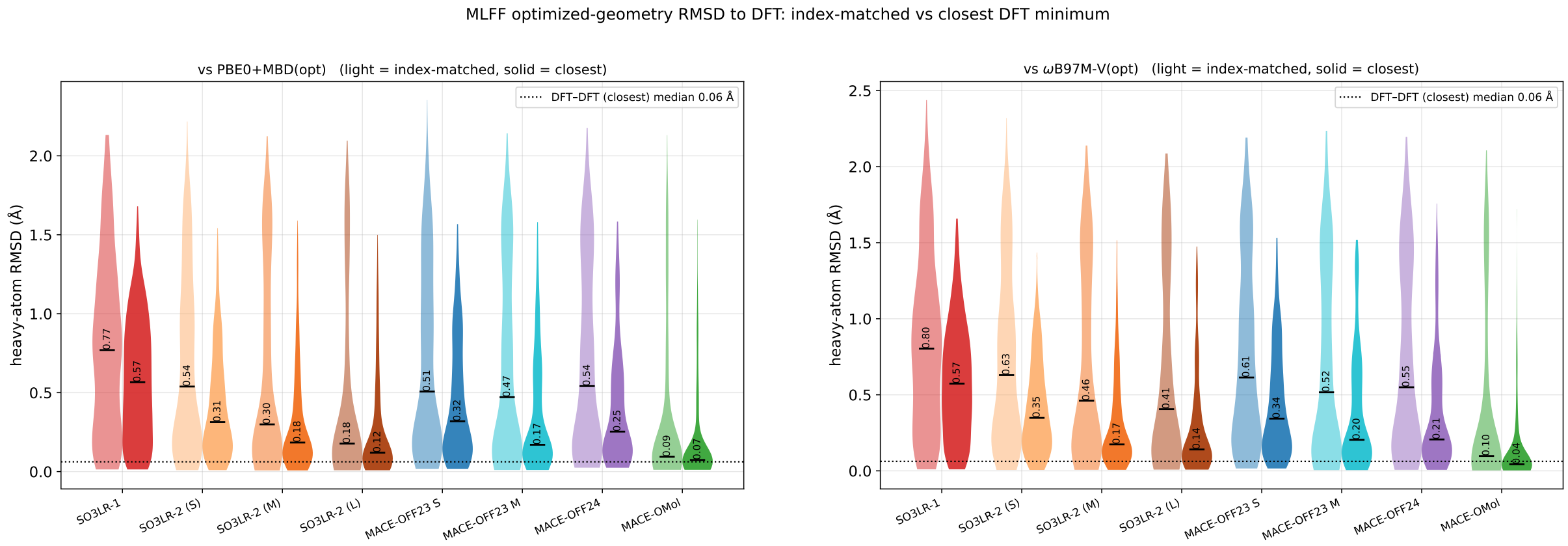

Figure S11: Heavy-atom RMSD of the MLFF-optimized dimer minima to the DFT-optimized structures, versus PBE0+MBD (left) and ωB97M-V (right). For each model the lighter violin is the index-matched RMSD (same starting conformer) and the solid violin is the closest-match RMSD (nearest DFT minimum of the dimer); medians are annotated. The dotted line marks the DFT–DFT (closest-match) baseline.

Table S27: Heavy-atom RMSD (Å) of the MLFF-optimized minima to the DFT-optimized structures, reported both index-matched (same starting conformer) and closest-match (nearest DFT minimum of the dimer), for the two DFT references.

Reference	Method	index-matched median	index-matched mean	closest DFT min. median	closest DFT min. mean
		heavy-atom RMSD (Å)			
PBE0+MBD	SO3LR-1	0.770	0.791	0.566	0.584
PBE0+MBD	SO3LR-2-S	0.539	0.671	0.314	0.416
PBE0+MBD	SO3LR-2-M	0.299	0.621	0.184	0.315
PBE0+MBD	SO3LR-2-L	0.177	0.471	0.120	0.269
PBE0+MBD	MACE-OFF23 S	0.529	0.700	0.325	0.456
PBE0+MBD	MACE-OFF23 M	0.460	0.656	0.174	0.368
PBE0+MBD	MACE-OFF24	0.541	0.701	0.253	0.413
PBE0+MBD	MACE-OMol	0.106	0.346	0.083	0.208
PBE0+MBD	DFT–DFT	0.112	0.451	0.062	0.155
ωB97M-V	SO3LR-1	0.803	0.844	0.574	0.595
ωB97M-V	SO3LR-2-S	0.629	0.730	0.348	0.418
ωB97M-V	SO3LR-2-M	0.461	0.689	0.174	0.315
ωB97M-V	SO3LR-2-L	0.406	0.628	0.141	0.291
ωB97M-V	MACE-OFF23 S	0.614	0.756	0.356	0.449
ωB97M-V	MACE-OFF23 M	0.523	0.691	0.204	0.390
ωB97M-V	MACE-OFF24	0.550	0.719	0.206	0.392
ωB97M-V	MACE-OMol	0.116	0.442	0.047	0.159
ωB97M-V	DFT–DFT	0.112	0.451	0.062	0.126

S12 Infrared benchmark

This section details the harmonic-IR benchmark summarised in Section 4 of the main text. Following our previous work [25], predicted spectra are compared to the experimental NIST35 gas-phase archive [26] through the shift-tolerant Pearson correlation $r^{\star}_{1/2}$ with x0.97 frequency scaling factor ; statistics are reported over the 4155-molecule subset common to all methods, with B3LYP-3c from Pracht *et al.* [27] as the external hybrid-DFT reference (no timing available). Wall times are per molecule on a single NVIDIA V100 GPU, and scaling exponents p come from a linear fit of $\log T$ against $\log N$ across the full set.

B3LYP-3c gives a median $r^{\star}_{1/2} = 0.73$ (IQR 0.64–0.80). SO3LR-1 reaches $r^{\star}_{1/2} = 0.63$ (0.52–0.74), confirming that the analytical MARS-IR Hessian reproduces the finite-difference reference within the benchmark uncertainty, and the three SO3LR-2 sizes improve monotonically with capacity (0.66 / 0.67 / 0.69 for S/M/L), closing the gap to B3LYP-3c to within 0.04 in the median. dxtb-GFN2 (0.63, 0.50–0.76) is on par with SO3LR-1 but with a broader distribution, and dxtb-GFN1 is the least accurate (0.60, 0.50–0.71). The two families scale very differently: the dxtb variants are near-quadratic ($p = 1.43$), whereas all four SO3LR variants are effectively size-independent ($p = 0.06$ for SO3LR-1 and 0.13, 0.14, 0.22 for SO3LR-2-S/M/L), the runtime being dominated by kernel and Hessian-assembly overhead rather than atom count for these small molecules.

Table S28: Harmonic-IR accuracy and scaling on the NIST35 gas-phase set (4155 molecules). $r^{\star}_{1/2}$ is the median shift-tolerant Pearson correlation with experiment (interquartile range in parentheses); p is the fitted wall-time scaling exponent ($T \propto N^p$). B3LYP-3c [27] is the external reference (no timing).

Method	$r^{\star}_{1/2}$ (IQR)	scaling p
B3LYP-3c (reference)	0.73 (0.64–0.80)	—
SO3LR-1	0.63 (0.52–0.74)	0.06
SO3LR-2-S	0.66 (0.55–0.75)	0.13
SO3LR-2-M	0.67 (0.57–0.76)	0.14
SO3LR-2-L	0.69 (0.60–0.77)	0.22
dxtb-GFN1	0.60 (0.50–0.71)	1.43
dxtb-GFN2	0.63 (0.50–0.76)	1.43

S12.1 Anharmonic spectra from molecular dynamics

MARS also computes infrared spectra from molecular dynamics through the dipole autocorrelation function (Section S3.3); such spectra contain anharmonicity, mode coupling and thermal broadening explicitly, at the cost of a trajectory. This subsection compares the two routes on a 100-molecule subset of the NIST35 gas-phase set, with the four SO3LR models and the same shift-tolerant-Pearson scoring as the rest of this section, including the same 0.97 scale factor applied to the MD frequencies.

S12.1.1 Molecular-dynamics protocol

Spectra were computed with `mars ir --md` in double precision on one NVIDIA V100 GPU per run. For each molecule and model, ten independent replicas were propagated simultaneously from the input geometry. Initial velocities were drawn from a Maxwell–Boltzmann distribution at 600 K, twice the target temperature, which compensates for the equipartition of kinetic into potential energy when starting from a minimum, and the replicas were equilibrated for 5 ps with a Langevin thermostat at 300 K (friction ≈ 1 ps^{-1}). Production then ran for 25 ps in the microcanonical ensemble with velocity Verlet and a 0.5 fs time step, recording the molecular dipole every 2.5 fs. The dipole is $\boldsymbol{\mu} = \sum_i q_i \mathbf{r}_i$ with the geometry-dependent partial charges predicted

by SO3LR. In total each molecule was sampled for 300 ps (ten replicas of 5 ps equilibration and 25 ps production)

S12.1.2 Results

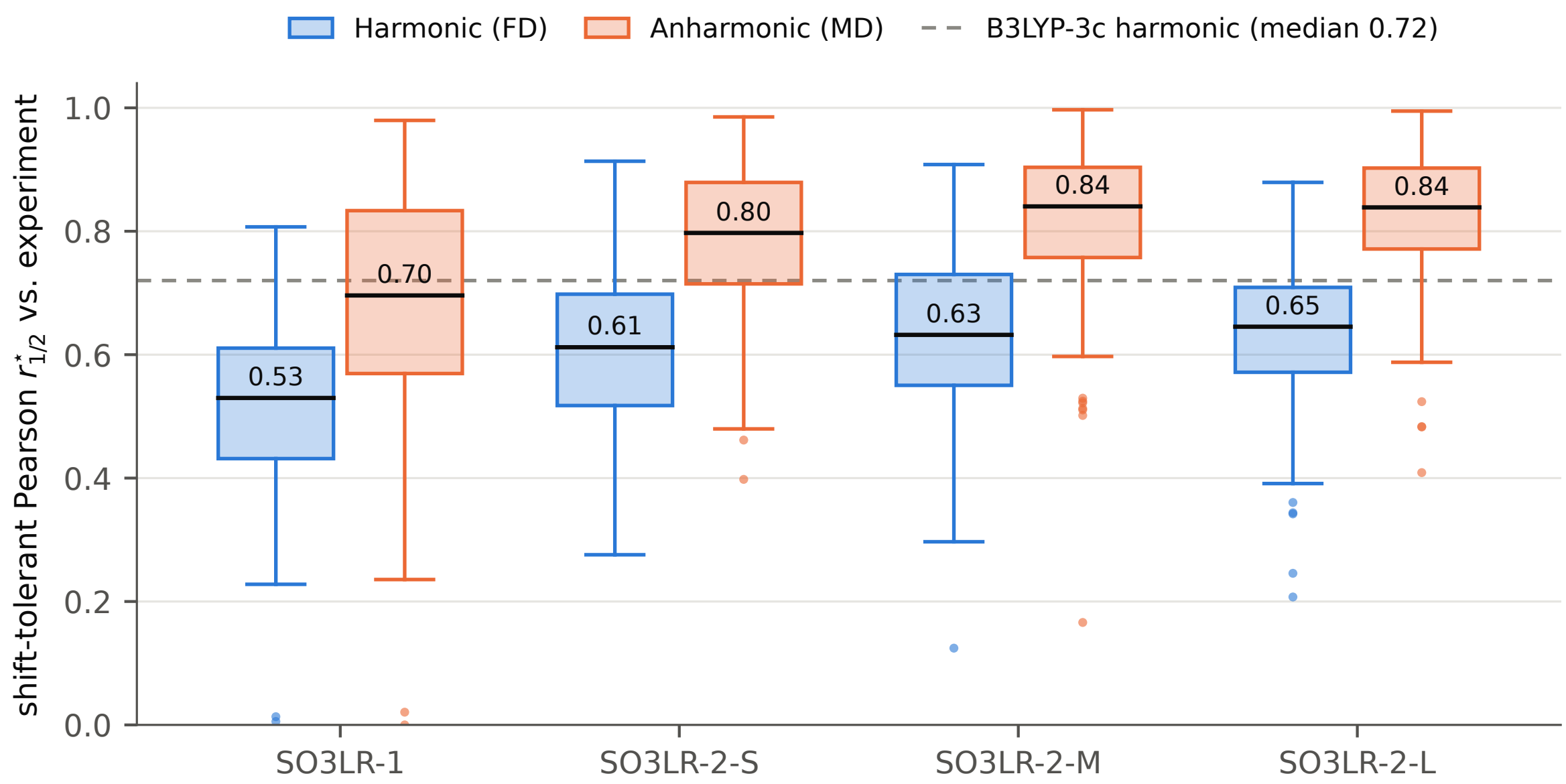

Figure S12: Agreement with experiment of harmonic and MD-based (anharmonic) infrared spectra for the 100-molecule subset, for the four SO3LR models, both scaled by 0.97. Shift-tolerant Pearson correlation $r^{\star}_{1/2}$ with the NIST35 gas-phase spectra; boxes give the interquartile range, whiskers 1.5×IQR, points are outliers, and medians are annotated. The dashed line marks the median of harmonic B3LYP-3c [27] on the same molecules.

Table S29: Harmonic versus MD-based spectra on the 100-molecule subset, both scaled by 0.97. $r^{\star}_{1/2}$: median shift-tolerant Pearson correlation with experiment (interquartile range); Δ: median paired difference MD − harmonic; "MD better": molecules on which the MD spectrum scores higher; p: two-sided Wilcoxon signed-rank test; r: median plain Pearson coefficient without shift; shift: median optimal rigid shift in cm^{-1}, positive when the computed bands lie above experiment. B3LYP-3c [27] is harmonic and given for reference.

	$r^{\star}_{1/2}$					r	shift
Model	harm.	MD	Δ	MD better	p	harm. / MD	harm. / MD
SO3LR-1	0.53 (0.43–0.61)	0.70 (0.57–0.83)	+0.17	95/100	4×10^{-16}	0.33 / 0.50	−6 / −9
SO3LR-2-S	0.61 (0.52–0.70)	0.80 (0.71–0.88)	+0.18	98/100	5×10^{-18}	0.47 / 0.71	+5 / −1
SO3LR-2-M	0.63 (0.55–0.73)	0.84 (0.76–0.90)	+0.17	99/100	4×10^{-18}	0.53 / 0.77	+6 / +1
SO3LR-2-L	0.65 (0.57–0.71)	0.84 (0.77–0.90)	+0.17	96/100	4×10^{-17}	0.55 / 0.78	+7 / +1
B3LYP-3c (harmonic)	0.72 (0.61–0.78)	—	—	—	—	—	—

With both routes scaled by the same factor, the MD spectra agree better with experiment than the harmonic ones for every model (Figure S12 and Table S29). The median $r^{\star}_{1/2}$ increases by 0.17–0.18, the MD spectrum scores higher on 95–99 of the 100 molecules, and the paired differences are significant for all four models ($p \leq 4 \times 10^{-16}$). The SO3LR-2 models reach a median MD $r^{\star}_{1/2}$ of 0.80–0.84, above harmonic B3LYP-3c on the same molecules (0.72); SO3LR-1 reaches 0.70. The ranking of the models is preserved, SO3LR-2-L and SO3LR-2-M being the

most accurate in both routes. The harmonic medians on this subset (0.53–0.65) are lower than on the full set above; all comparisons here are paired on identical molecules.

Figure S13 shows three molecules with SO3LR-2-L, chosen as the largest gain, a case near the median gain, and the largest loss; the NIST35 reference spectra are licensed and are not reproduced here, so only the two computed spectra are shown, each scored against the (unplotted) experimental spectrum. For methyl isothiocyanate, the largest gain, the harmonic spectrum places a single narrow and far too intense N=C=S stretch, whereas the MD spectrum reproduces its breadth and relative weight. 1-Methyl-1H-pyrrole, close to the median gain, is well described by both routes, and the MD spectrum improves the relative intensities across the fingerprint region. For octyl thioglycolate, the largest loss, the harmonic route places a similarly too-narrow, too-intense band near 1750 cm^{-1} that happens to fall close to the reference position, while the MD spectrum spreads the corresponding intensity into the broad, weaker fingerprint pattern typical of a flexible 33-atom chain, which scores lower under the shift-tolerant correlation despite being the qualitatively more realistic line shape.

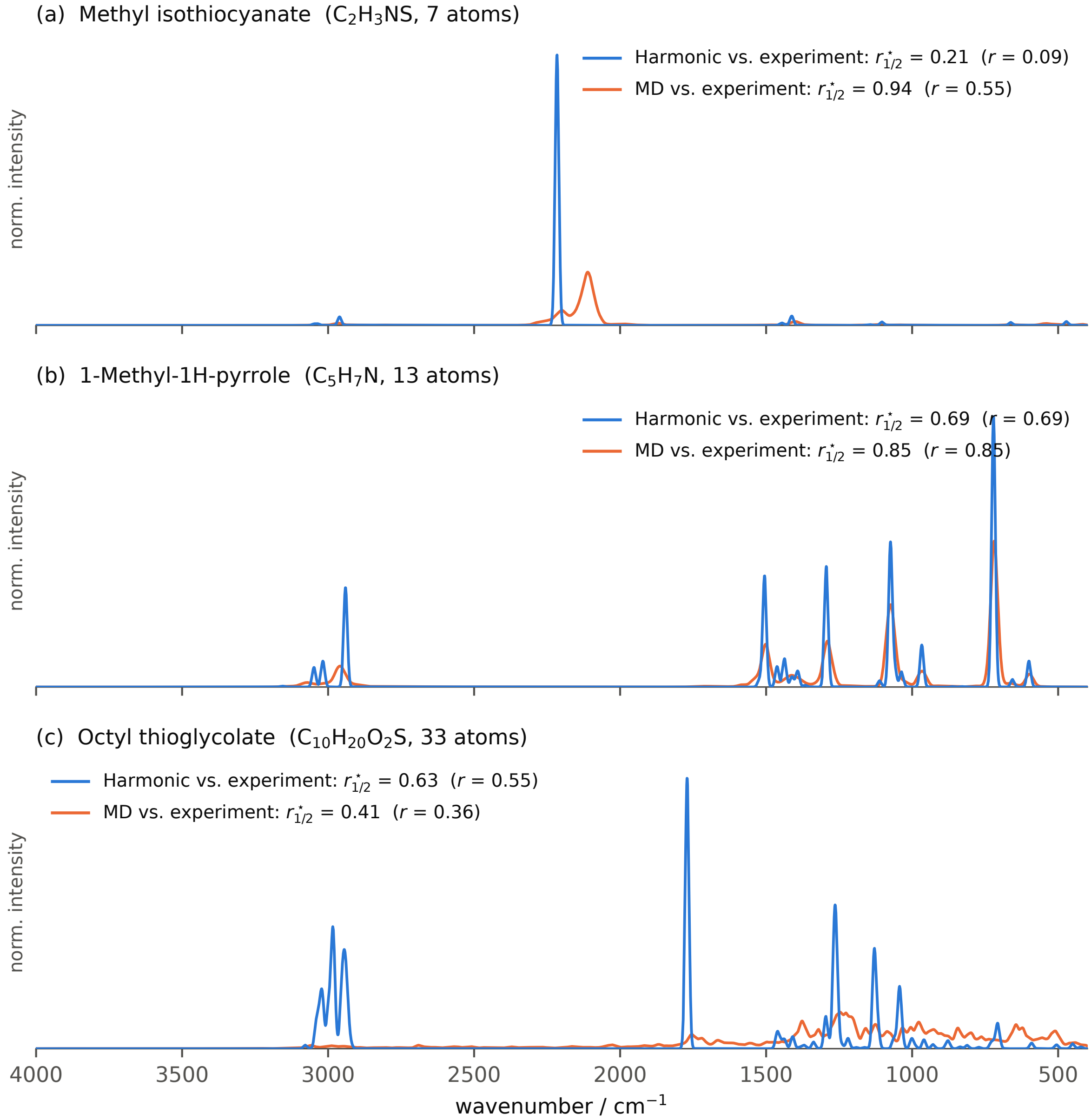

Figure S13: Harmonic (blue) and MD-based (orange) infrared spectra computed with SO3LR-2-L, both scaled by 0.97, on a common wavenumber grid, each normalised to unit area as scored. The NIST35 experimental spectra are not reproduced here (licensed database); the legend gives the shift-tolerant Pearson correlation $r^{\star}_{1/2}$ and, in parentheses, the plain Pearson coefficient r, both against the unplotted experimental spectrum. (a) Methyl isothiocyanate, the largest gain from MD; (b) 1-methyl-1H-pyrrole, close to the median gain; (c) octyl thioglycolate, the largest loss.

S12.1.3 Computational cost

Table S30 gives the total wall time of the full 4155-molecule NIST35-common set for the harmonic route, finite differences against the analytical Hessian, for all four SO3LR models; Table S31 gives the same comparison, plus MD, on the 100-molecule subset. The molecules of the subset are listed below, with the shift-tolerant Pearson correlation $r^{\star}_{1/2}$ of the harmonic (H) and MD spectra (both scaled by 0.97) for SO3LR-1 and SO3LR-2-L. Names are those of the NIST record; N is the number of atoms and rot. the number of rotatable bonds from the MARS topology heuristic.

Table S30: Total wall time (GPU-hours, one NVIDIA V100) for the harmonic route on the full 4155-molecule NIST35-common set, finite differences (FD) versus the analytical Hessian. n_{FD}, n_{an}: molecules with a valid timing log for each method (SO3LR-2-L failed to converge the analytical Hessian on 34 molecules due to memory limitations; each column's total is summed only over its own valid entries).

Model	FD (h)	analytical (h)	analytical / FD	n_{FD}	n_{an}
SO3LR-1	73.7	90.5	1.23×	4155	4155
SO3LR-2-S	41.2	53.1	1.29×	4155	4155
SO3LR-2-M	45.5	59.4	1.31×	4155	4155
SO3LR-2-L	53.0	68.4	1.29×	4155	4121

Table S31: Total wall time (GPU-hours, one NVIDIA V100) for the 100-molecule subset: harmonic finite differences (FD), harmonic analytical Hessian, and MD (ten replicas, 300 ps in total per molecule, Section S12.1.1).

Model	FD (h)	analytical (h)	MD (h)	MD / FD	MD / analytical
SO3LR-1	1.78	2.19	9.68	5.4×	4.4×
SO3LR-2-S	0.96	1.25	7.68	8.0×	6.2×
SO3LR-2-M	1.09	1.43	9.70	8.9×	6.8×
SO3LR-2-L	1.26	1.67	14.68	11.6×	8.8×

Table S32: The 100 molecules of the MD-IR subset with their scores

						SO3LR-1		SO3LR-2-L	
#	CAS	Name	Formula	N	rot.	H	MD	H	MD
1	74-86-2	Acetylene	C_2H_2	4	1	0.63	0.86	0.68	0.88
2	75-02-5	Fluoroethene	C_2H_3F	6	0	0.36	0.61	0.57	0.90
3	463-49-0	1,2-Propadiene	C_3H_4	7	0	0.35	0.73	0.40	0.81
4	556-61-6	Methyl isothiocyanate	C_2H_3NS	7	2	0.01	0.00	0.21	0.94
5	624-83-9	Methylisocyanate	C_2H_3NO	7	2	0.34	0.90	0.59	0.97
6	107-19-7	2-Propyn-1-ol	C_3H_4O	8	3	0.58	0.84	0.57	0.84
7	290-87-9	1,3,5-Triazine	$C_3H_3N_3$	9	0	0.58	0.82	0.65	0.86
8	75-08-1	Ethanethiol	C_2H_6S	9	2	0.71	0.86	0.59	0.67
9	67-64-1	Acetone	C_3H_6O	10	0	0.54	0.78	0.50	0.74
10	1003-31-2	2-Thiophenecarbonitrile	C_5H_3NS	10	0	0.37	0.56	0.65	0.84
11	922-67-8	Methyl propiolate	$C_4H_4O_2$	10	2	0.60	0.81	0.74	0.95
12	624-92-0	Dimethyl disulfide	$C_2H_6S_2$	10	3	0.54	0.69	0.47	0.61
13	540-54-5	1-Chloropropane	C_3H_7Cl	11	2	0.73	0.89	0.69	0.66
14	151-18-8	β-Aminopropionitrile	$C_3H_6N_2$	11	3	0.49	0.81	0.42	0.82
15	16879-02-0	6-Chloro-2-pyridinol	C_5H_4ClNO	12	0	0.54	0.75	0.62	0.82
16	513-37-1	1-Chloro-2-methyl-1-propene	C_4H_7Cl	12	0	0.48	0.66	0.62	0.86
17	5875-24-1	3-Chloropropionamide	C_3H_6ClNO	12	1	0.64	0.88	0.64	0.89
18	2809-69-0	2,4-Hexadiyne	C_6H_6	12	5	0.26	0.26	0.34	0.64
19	1003-10-7	γ-Thiobutyrolactone	C_4H_6OS	12	3	0.79	0.98	0.79	0.97
20	628-20-6	4-Chlorobutanenitrile	C_4H_6ClN	12	3	0.29	0.40	0.39	0.62
21	108-98-5	Thiophenol	C_6H_6S	13	0	0.51	0.68	0.62	0.89
22	77-77-0	Vinyl sulfone	$C_4H_6O_2S$	13	0	0.61	0.78	0.76	0.90
23	16839-97-7	2-Methoxythiophene	C_5H_6OS	13	1	0.53	0.83	0.58	0.87
24	96-54-8	1-Methyl-1H-pyrrole	C_5H_7N	13	1	0.61	0.87	0.69	0.85
25	868-85-9	Dimethyl phosphite	$C_2H_7O_3P$	13	4	0.23	0.56	0.55	0.85
26	616-45-5	2-Pyrrolidinone	C_4H_7NO	13	3	0.78	0.97	0.77	0.97
27	2346-00-1	4,5-Dihydro-2-methylthiazole	C_4H_7NS	13	3	0.57	0.71	0.51	0.67
28	348-54-9	2-Fluorobenzenamine	C_6H_6FN	14	0	0.49	0.71	0.56	0.88
29	1711-07-5	3-Fluorobenzoyl chloride	C_7H_4ClFO	14	0	0.39	0.44	0.61	0.75
30	404-71-7	3-Fluorophenyl isocyanate	C_7H_4FNO	14	1	0.80	0.94	0.84	0.98
31	592-57-4	1,3-Cyclohexadiene	C_6H_8	14	1	0.45	0.69	0.53	0.66
32	5006-22-4	Cyclobutanecarboxylic acid chloride	C_5H_7ClO	14	4	0.67	0.88	0.63	0.82
33	110-56-5	1,4-Dichlorobutane	$C_4H_8Cl_2$	14	3	0.60	0.72	0.50	0.66
34	1885-14-9	Phenyl chloroformate	$C_7H_5ClO_2$	15	0	0.35	0.45	0.48	0.65
35	345-35-7	1-(Chloromethyl)-2-fluorobenzene	C_7H_6ClF	15	0	0.43	0.56	0.79	0.93
36	2190-48-9	3-Chloro-3-methyl-1-butene	C_5H_9Cl	15	2	0.46	0.63	0.39	0.48
37	2396-63-6	1,6-Heptadiyne	C_7H_8	15	6	0.55	0.90	0.66	0.93
38	625-84-3	2,5-Dimethylpyrrole	C_6H_9N	16	0	0.30	0.39	0.36	0.63
39	5402-55-1	2-Thiopheneethanol	C_6H_8OS	16	2	0.57	0.78	0.54	0.83
40	121-45-9	Trimethyl phosphite	$C_3H_9O_3P$	16	6	0.45	0.24	0.84	0.93
41	10133-34-3	4-Nitrobenzo[b]thiophene	$C_8H_5NO_2S$	17	0	0.49	0.57	0.71	0.90
42	673-32-5	1-Propynylbenzene	C_9H_8	17	2	0.42	0.65	0.66	0.77
43	443-82-3	3-Fluoro-o-xylene	C_8H_9F	18	0	0.54	0.64	0.79	0.88
44	563-79-1	2,3-Dimethyl-2-butene	C_6H_{12}	18	0	0.66	0.88	0.62	0.90
45	936-46-9	4-Phenylisothiazole	C_9H_7NS	18	1	0.55	0.64	0.67	0.88
46	459-47-2	p-Ethylfluorobenzene	C_8H_9F	18	1	0.49	0.65	0.79	0.92
47	623-81-4	Diethyl sulfite	$C_4H_{10}O_3S$	18	6	0.37	0.62	0.72	0.95
48	6415-12-9	Tetramethylhydrazine	$C_4H_{12}N_2$	18	5	0.66	0.86	0.61	0.87
49	584-84-9	2,4-Diisocyanato-1-methylbenzene	$C_9H_6N_2O_2$	19	2	0.78	0.95	0.81	0.99
50	51575-85-0	7-Chloro-3-heptyne	$C_7H_{11}Cl$	19	6	0.60	0.83	0.69	0.76
51	1745-81-9	2-(2-Propenyl)phenol	$C_9H_{10}O$	20	0	0.51	0.79	0.45	0.82
52	1746-13-0	Allylphenyl ether	$C_9H_{10}O$	20	1	0.69	0.90	0.68	0.92
53	101-05-3	Anilazine	$C_9H_5Cl_3N_4$	21	0	0.28	0.39	0.76	0.84
54	938-95-4	p-Chloro-methylphenyl acetic acid	$C_9H_9ClO_2$	21	1	0.65	0.87	0.70	0.93
55	1605-65-8	Bis(dimethylamino)phosphinic chloride	$C_4H_{12}ClN_2OP$	21	6	0.40	0.64	0.70	0.89
56	92-85-3	Thianthrene	$C_{12}H_8S_2$	22	0	0.49	0.53	0.84	0.91
57	344-62-7	N-[2-(Trifluoromethyl)phenyl]acetamide	$C_9H_8F_3NO$	22	0	0.40	0.49	0.63	0.82
58	145-73-3	Endothal	$C_8H_{10}O_5$	23	8	0.65	0.68	0.65	0.64
59	6298-72-2	2,5-Bis(chloromethyl)-p-xylene	$C_{10}H_{12}Cl_2$	24	0	0.37	0.55	0.65	0.79
60	822-06-0	1,6-Diisocyanatohexane	$C_8H_{12}N_2O_2$	24	9	0.57	0.83	0.73	0.98
61	4426-83-9	1-Isothiocyanatoheptane	$C_8H_{15}NS$	25	8	0.01	0.02	0.25	0.91
62	831-91-4	[(Phenylmethyl)thio]benzene	$C_{13}H_{12}S$	26	1	0.63	0.72	0.64	0.82
63	553-27-5	N,N-Bis(2-chloroethyl)benzenamine	$C_{10}H_{13}Cl_2N$	26	4	0.65	0.83	0.62	0.84
64	494-19-9	10,11-Dihydro-5H-dibenz[b,f]azepine	$C_{14}H_{13}N$	28	1	0.68	0.83	0.81	0.94
65	15570-10-2	4-(1,1-Dimethylethyl)-2-methylbenzenethiol	$C_{11}H_{16}S$	28	3	0.61	0.68	0.72	0.85
66	27314-13-2	Norflurazon	$C_{12}H_9ClF_3N_3O$	29	2	0.44	0.51	0.68	0.78
67	52222-87-4	6-Benzoyl-2-naphthol	$C_{17}H_{12}O_2$	31	0	0.53	0.76	0.60	0.79
68	2741-38-0	Allyldiphenylphosphine	$C_{15}H_{15}P$	31	1	0.55	0.50	0.57	0.70

#	CAS	Name	Formula	N	rot.	H	MD	H	MD
69	22424-58-4	5-Benzyloxy-2-nitrotoluene	$C_{14}H_{13}NO_3$	31	1	0.52	0.72	0.65	0.83
70	139-40-2	Propazine	$C_9H_{16}ClN_5$	31	6	0.30	0.41	0.68	0.84
71	119-91-5	2,2'-Biquinoline	$C_{18}H_{12}N_2$	32	0	0.40	0.51	0.53	0.69
72	959-02-4	5,12-Dihydrotetracene	$C_{18}H_{14}$	32	0	0.41	0.63	0.70	0.81
73	3624-90-6	o-(p-Toluylsulfonamido)aniline	$C_{13}H_{14}N_2O_2S$	32	1	0.43	0.70	0.62	0.85
74	6375-27-5	Acetoacet-2,5-dimethoxyanilide	$C_{12}H_{15}NO_4$	32	2	0.49	0.69	0.34	0.52
75	1026-92-2	Diallyl terephthalate	$C_{14}H_{14}O_4$	32	2	0.49	0.69	0.63	0.89
76	18719-43-2	Diethyl (3-chloropropyl)malonate	$C_{10}H_{17}ClO_4$	32	7	0.73	0.89	0.79	0.96
77	334-56-5	1-Fluorodecane	$C_{10}H_{21}F$	32	9	0.78	0.88	0.73	0.88
78	3933-94-6	4-Phenoxy-1,1'-biphenyl	$C_{18}H_{14}O$	33	0	0.76	0.90	0.88	0.94
79	1705-85-7	6-Methylchrysene	$C_{19}H_{14}$	33	0	0.60	0.70	0.67	0.79
80	574-06-1	Benzoin acetate	$C_{16}H_{14}O_3$	33	1	0.66	0.86	0.78	0.92
81	7664-80-4	Octyl thioglycolate	$C_{10}H_{20}O_2S$	33	9	0.56	0.69	0.63	0.41
82	2128-93-0	4-Benzoylbiphenyl	$C_{19}H_{14}O$	34	0	0.41	0.52	0.54	0.65
83	3379-38-2	1,3-Diphenoxybenzene	$C_{18}H_{14}O_2$	34	0	0.68	0.84	0.69	0.91
84	3678-70-4	Diphenyl-2-pyridylmethane	$C_{18}H_{15}N$	34	0	0.51	0.66	0.63	0.78
85	4325-74-0	1,2'-Binaphthyl	$C_{20}H_{14}$	34	0	0.49	0.50	0.62	0.61
86	112-51-6	Dipentyl disulfide	$C_{10}H_{22}S_2$	34	11	0.81	0.91	0.80	0.91
87	613-42-3	4-Benzylbiphenyl	$C_{19}H_{16}$	35	0	0.48	0.60	0.67	0.81
88	519-73-3	Triphenylmethane	$C_{19}H_{16}$	35	0	0.49	0.63	0.69	0.82
89	487-10-5	1,1'-Azonaphthalene	$C_{20}H_{14}N_2$	36	1	0.51	0.65	0.67	0.76
90	3695-77-0	Triphenylmethylmercaptan	$C_{19}H_{16}S$	36	1	0.46	0.64	0.64	0.75
91	1572-46-9	9-Benzylfluorene	$C_{20}H_{16}$	36	1	0.53	0.72	0.68	0.81
92	33245-39-5	Fluchloralin	$C_{12}H_{13}ClF_3N_3O_4$	36	5	0.54	0.67	0.48	0.59
93	2638-94-0	4,4-Azobis-4-cyanovaleric acid	$C_{12}H_{16}N_4O_4$	36	11	0.46	0.80	0.46	0.79
94	5824-40-8	Tritylamine	$C_{19}H_{17}N$	37	1	0.58	0.75	0.59	0.83
95	13171-21-6	Phosphamidon	$C_{10}H_{19}ClNO_5P$	37	9	0.58	0.78	0.71	0.82
96	1071-31-4	2,2,7,7-Tetramethyloctane	$C_{12}H_{26}$	38	11	0.79	0.90	0.86	0.96
97	58-40-2	Promazine	$C_{17}H_{20}N_2S$	40	6	0.39	0.54	0.81	0.91
98	102-85-2	Tributyl phosphite	$C_{12}H_{27}O_3P$	43	15	0.51	0.57	0.74	0.87
99	5717-37-3	(Carbethoxyethylidine)triphenyl phosphora	$C_{23}H_{23}O_2P$	49	2	0.32	0.43	0.41	0.48
100	1663-45-2	1,2-Bis(diphenylphosphino)ethane	$C_{26}H_{24}P_2$	52	3	0.58	0.36	0.71	0.80

S13 Alanine dipeptide microsolvation: model comparison

Table S33 reports the relative conformer energies of alanine dipeptide across all benchmarked potentials (SO3LR-1; SO3LR-2-S/M/L; MACE-off small/medium; MACE-OFF24; MACE-OMol) in the gas phase, ether, and water, compared with the PCM MP2/cc-pVTZ//MP2/6-31G** reference of Wang and Duan [28] and with the PBE0+MBD and ωB97M-V reference used throughout this work in gas phase, ether and water (implicit solvent on the benchmark conformer geometry in ether/water; see the table note). Gas-phase values are single-point energies on the benchmark conformer geometries; water and ether values are means over five microsolvation replicas with the standard deviation in parentheses (the two DFT references are single calculations, not replica means). All values use the single-point per-atom (embedded) solute energy, which preserves conformer identity (a full relaxation collapses the non-minimum β_2 and α conformers).

Table S33: Relative conformer energies of alanine dipeptide (kcal/mol, referenced to C7eq) from MARS single-point embedded energies, compared with the PCM MP2/cc-pVTZ//MP2/6-31G** reference of Wang and Duan [28] and with the PBE0+MBD and ωB97M-V references. Vacuum values are from the gas-phase conformer benchmark; water and ether values are means over five microsolvation replicas with the standard deviation in parentheses (PBE0+MBD and ωB97M-V water/ether values are single implicit-solvent calculations.. Standard deviations are evaluated from replica energies referenced to the per-replica conformer mean, so the reference conformer (C7eq) also carries an uncertainty.

Method	C7eq	C5	C7ax	β_2	α_L	α
Gas phase						
Wang 2004 (PCM)	0.00	1.47	2.50	3.25	4.52	3.27
PBE0+MBD	0.00	1.48	2.14	3.49	5.42	7.05
ωB97M-V	0.00	1.28	2.41	3.17	4.82	6.46
SO3LR-1	0.00	1.36	1.94	3.59	4.94	5.80
SO3LR-2-S	0.00	1.34	1.61	3.57	4.96	6.06
SO3LR-2-M	0.00	0.99	2.24	3.09	4.70	5.79
SO3LR-2-L	0.00	1.11	2.44	3.21	5.21	6.36
MACE-OFF23 S	0.00	0.85	1.77	2.43	4.04	5.05
MACE-OFF23 M	0.00	1.22	2.00	3.15	4.85	6.06
MACE-OFF24	0.00	1.20	1.91	2.83	4.71	5.90
MACE-OMol	0.00	1.23	2.44	3.16	4.85	6.40
Ether						
Wang 2004 (PCM)	0.00	0.10	1.40	2.16	2.73	1.75
PBE0+MBD	0.00	0.46	1.94	1.37	3.55	3.32
ωB97M-V	0.00	0.43	2.10	1.29	2.96	3.14
SO3LR-1	0.00 (0.9)	0.76 (2.0)	2.08 (1.0)	1.15 (1.1)	3.99 (1.8)	4.10 (0.8)
SO3LR-2-S	0.00 (0.5)	0.45 (1.2)	1.40 (1.1)	1.29 (1.2)	3.54 (2.1)	4.27 (0.8)
SO3LR-2-M	0.00 (0.4)	0.20 (1.1)	1.35 (0.5)	1.12 (0.9)	2.81 (1.9)	3.88 (0.8)
SO3LR-2-L	0.00 (0.4)	1.13 (1.7)	1.36 (1.1)	1.46 (0.9)	3.75 (2.0)	4.22 (1.2)
MACE-OFF23 S	0.00 (1.2)	1.42 (2.6)	0.50 (1.1)	0.40 (1.8)	2.53 (3.1)	2.55 (0.8)
MACE-OFF23 M	0.00 (1.1)	0.58 (1.8)	0.55 (1.1)	0.96 (1.7)	3.10 (3.2)	3.34 (1.4)
MACE-OFF24	0.00 (0.7)	0.32 (1.7)	0.49 (1.6)	1.01 (1.5)	3.27 (3.3)	3.78 (1.7)
MACE-OMol	0.00 (0.6)	0.86 (1.5)	1.49 (1.2)	1.43 (1.3)	3.25 (2.3)	4.16 (1.0)
Water						
Wang 2004 (PCM)	0.00	-0.92	1.77	0.35	0.35	-0.84
PBE0+MBD	0.00	-0.18	1.96	-0.24	1.76	1.98
ωB97M-V	0.00	0.15	1.91	0.44	1.87	2.34
SO3LR-1	0.00 (1.5)	0.96 (1.5)	3.39 (0.9)	0.57 (1.6)	5.01 (3.2)	4.52 (2.0)
SO3LR-2-S	0.00 (1.4)	0.52 (0.9)	3.47 (1.3)	-0.14 (1.6)	4.91 (2.8)	4.77 (1.8)
SO3LR-2-M	0.00 (2.0)	0.52 (1.6)	5.21 (1.4)	0.73 (2.2)	5.96 (3.3)	6.04 (2.6)
SO3LR-2-L	0.00 (1.3)	0.71 (1.5)	4.28 (0.8)	0.26 (1.8)	5.31 (3.3)	6.43 (2.2)
MACE-OFF23 S	0.00 (1.2)	1.19 (2.7)	1.27 (1.0)	-1.83 (0.9)	3.62 (3.8)	2.83 (1.4)
MACE-OFF23 M	0.00 (2.5)	1.19 (2.4)	7.13 (1.4)	1.26 (2.2)	6.09 (3.8)	8.38 (2.9)
MACE-OFF24	0.00 (2.4)	1.94 (2.2)	8.61 (1.9)	1.60 (1.9)	8.40 (3.8)	10.32 (2.8)
MACE-OMol	0.00 (1.3)	0.86 (1.6)	6.74 (1.1)	0.36 (1.6)	5.19 (3.2)	7.50 (2.9)

The PBE0+MBD and ωB97M-V water/ether values use an implicit (continuum) solvent (FHI-aims MPE, ORCA CPCM) on the benchmark conformer geometry.

Beyond the six discrete benchmark conformers, we scanned the full ϕ/ψ backbone Ramachandran surface at 5° resolution (72×72 = 5184 points) for both solvents. At each grid point the solute backbone dihedrals were set by rigid rotation of a single common conformation and held fixed while the surrounding microsolvation shell was relaxed with SO3LR-1 , so it is identical across models by construction and serves as a control. Every one of the 5184 SO3LR-relaxed geometries per solvent was then re-scored by single point with every other potential, giving the

same ϕ/ψ surface as seen by each model. Figures S14 and S15 show E_{embed} (solute atomic-sum energy) on this grid for all eight potentials.

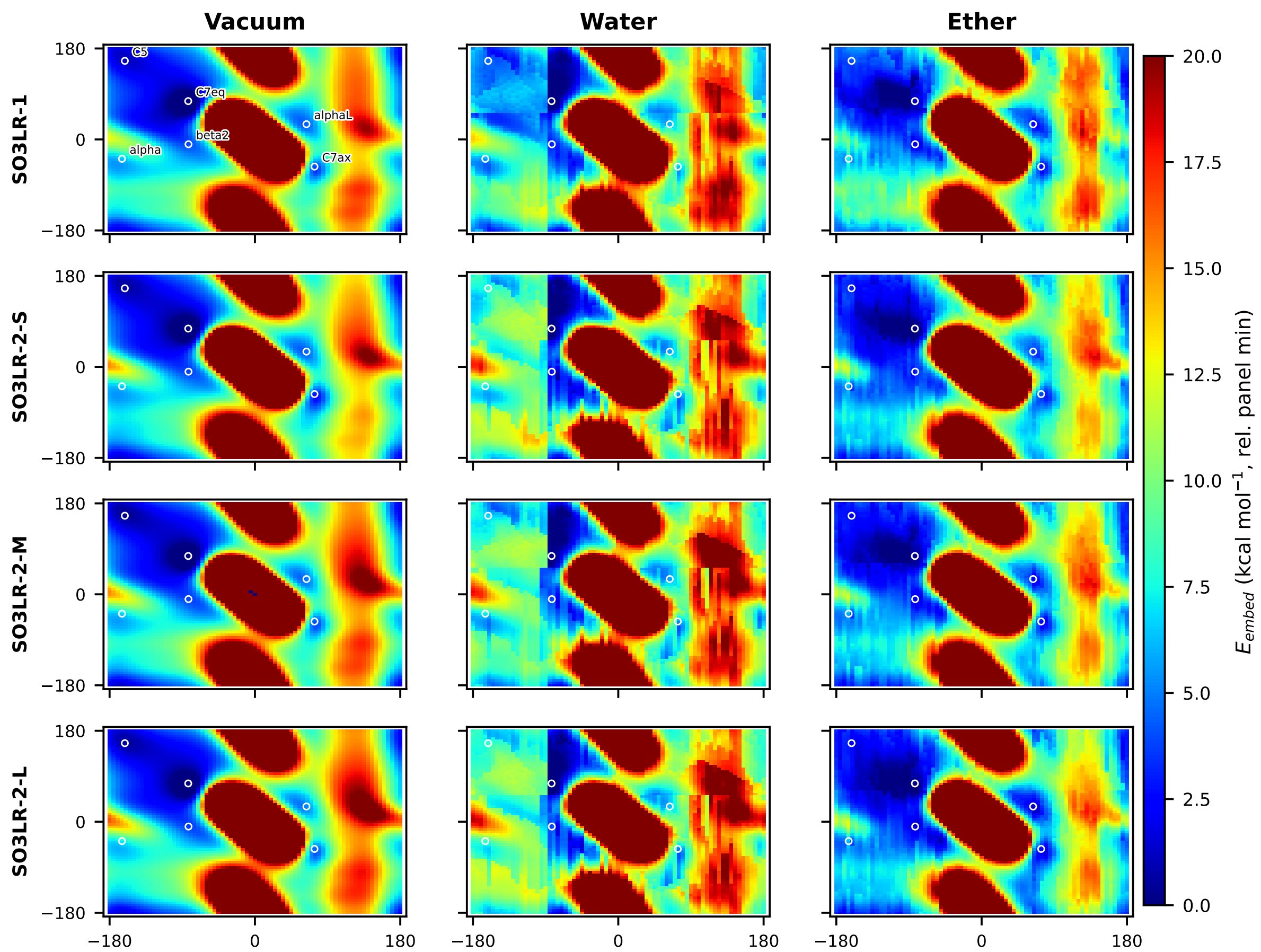

Figure S14: Solute atomic-sum energy E_{embed} (kcal/mol, relative to each panel's own 1st-percentile value, capped at 20 kcal/mol) on the 5° ϕ/ψ scan described in the text, for SO3LR-1 and SO3LR-2-S/M/L (rows), in the gas phase, water and ether (columns). White circles mark the six benchmark conformers (labeled in the top-left panel). Water and ether geometries are SO3LR-1-relaxed at every grid point; the gas-phase column is the shared rigid solute and is identical across all rows.

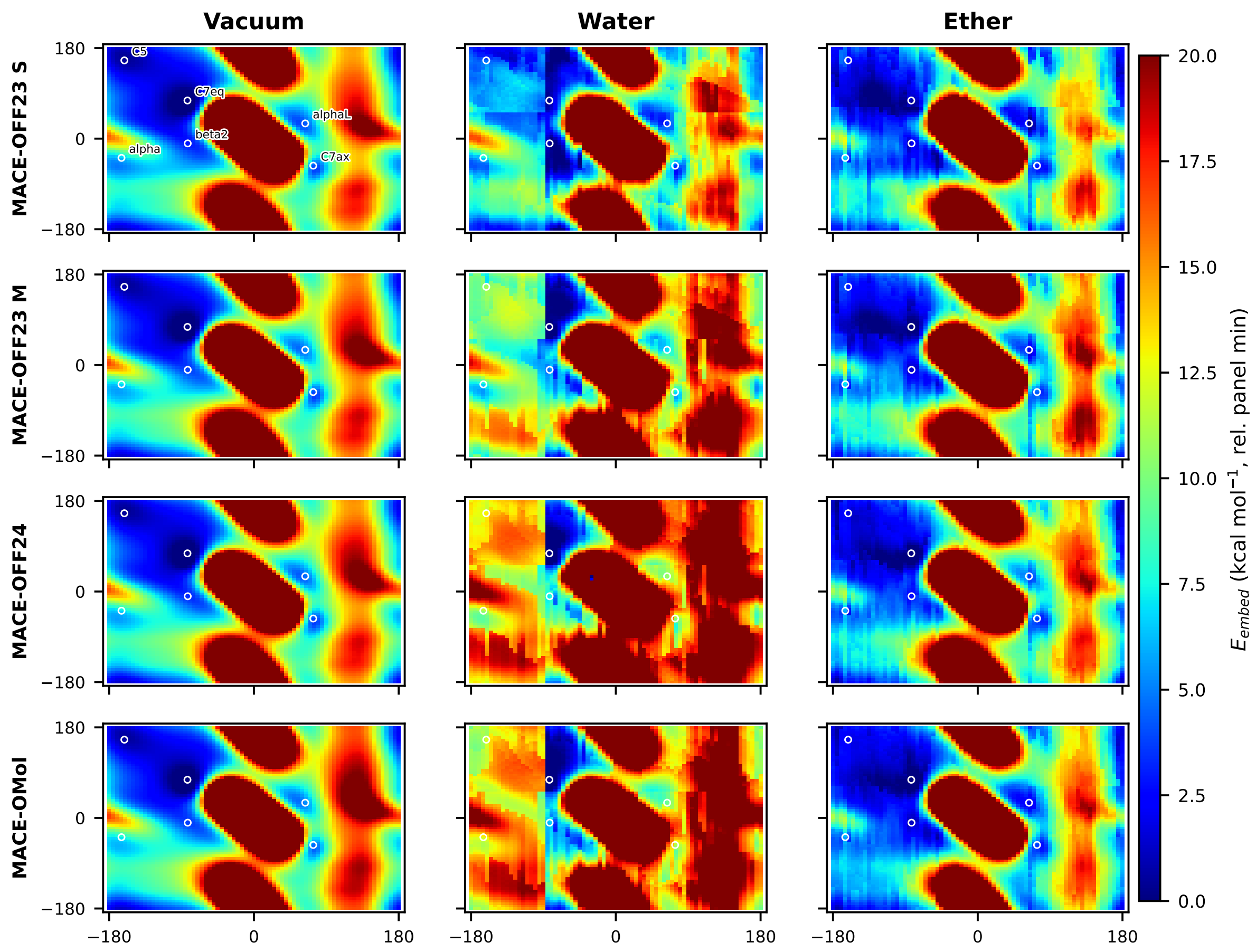

Figure S15: As Figure S14, for MACE-OFF23 S/M, MACE-OFF24 and MACE-OMol.

S13.1 Geometric versus barostat microsolvation

MARS provides two builders for the explicit solvent shell (Section S4.7 and the preceding subsections): the default *geometric* layer-by-layer builder, which packs concentric shells around the solute and relaxes the whole cluster, and the *barostat* builder, which compresses a solute-free solvent droplet inside a spherical moving wall and then swaps the solute, kept frozen, into a central cavity. All solvation results reported in the main text (alanine dipeptide and 2-methoxycyclohexanone) were obtained with the geometric builder. To document how this choice affects the results, the alanine dipeptide benchmark was rebuilt with the barostat builder using the same target solvent counts (100 H_2O or 25 Et_2O molecules), the same six conformers and five independent replicas per conformer, and both sets of clusters were evaluated with SO3LR-1, the potential that builds all clusters, on each protocol's own geometry.

Energetics are reported as the embedded solute energy E_{embed} (sum of the 22 solute atomic energies, as in the main text) relative to C7eq of the same replica, with mean and standard deviation over replicas (Figure S16A, Table S35). Because the two builders treat the solute geometry differently, we also report $E_{\text{embed}} - E_{\text{isolated}}$, where E_{isolated} is the SO3LR-1 energy of the solute alone at the same geometry, which isolates the solvent-induced shift of the solute energy (Figure S16B). Compactness is quantified by (i) the radial solvent mass-density profile around the solute centre of mass (Figure S16C), (ii) the radius of gyration R_g of the whole cluster and the effective density ρ_{eff} (cluster mass divided by the volume of the uniform sphere with the same R_g, i.e. $R = \sqrt{5/3}\, R_g$), (iii) the largest solvent-atom distance $R_{\max}$ from the solute centre of mass, and (iv) the number of first-shell solvent molecules (any atom within 3.5 Å of a solute atom) (Table S34). ρ_{eff} is only meaningful as a relative measure between the two builders: for

small, non-uniform clusters it exceeds the bulk density of either solvent.

In water the two builders give relative embedded energies that agree within 1.0 kcal mol^{-1} for all conformers, well inside the replica-to-replica scatter (SD of 0.8–3.2 kcal mol^{-1}). In ether they differ by at most 1.5 kcal mol^{-1} for C5, C7ax and β_2, and by 3.4 and 3.3 kcal mol^{-1} for α_L and α, for which the barostat builder gives higher energies (+7.4 against +4.0 and +7.4 against +4.1 kcal mol^{-1}). This difference comes mostly from the solute geometry: the barostat builder keeps the solute frozen at the benchmark geometry, where the isolated α_L and α solutes lie 4.9 and 5.8 kcal mol^{-1} above C7eq, whereas the geometric builder relaxes the solute together with the shell (isolated-solute energies of α_L and α of +3.8 and +3.2 kcal mol^{-1} in water and +2.6 and +4.5 kcal mol^{-1} in ether). When the isolated-solute energy is subtracted (Figure S16B and lower block of Table S35), the differences between the builders are at most 2.1 kcal mol^{-1} in water and 2.3 kcal mol^{-1} in ether.

In water the barostat clusters are slightly more compact than the geometric ones (R_g 6.6 against 6.9 Å, R_{max} 9.9 against 11.2 Å, ρ_{eff} 1.18 against 1.07 g cm^{-3}), with a similar number of first-shell molecules (36 against 37). In diethyl ether the barostat clusters are markedly more compact: R_g is 6.9 against 7.6 Å, the outer radius 10.1 against 13.4 Å, ρ_{eff} 1.11 against 0.85 g cm^{-3}, and 17.9 against 13.8 solvent molecules contact the solute; the barostat clusters are also more reproducible (replica SD of R_g of 0.03 against 0.28 Å). The geometric ether shell has a diffuse tail beyond 10 Å, whereas the barostat shell is truncated by the wall and shows a density maximum close to it (Figure S16C).

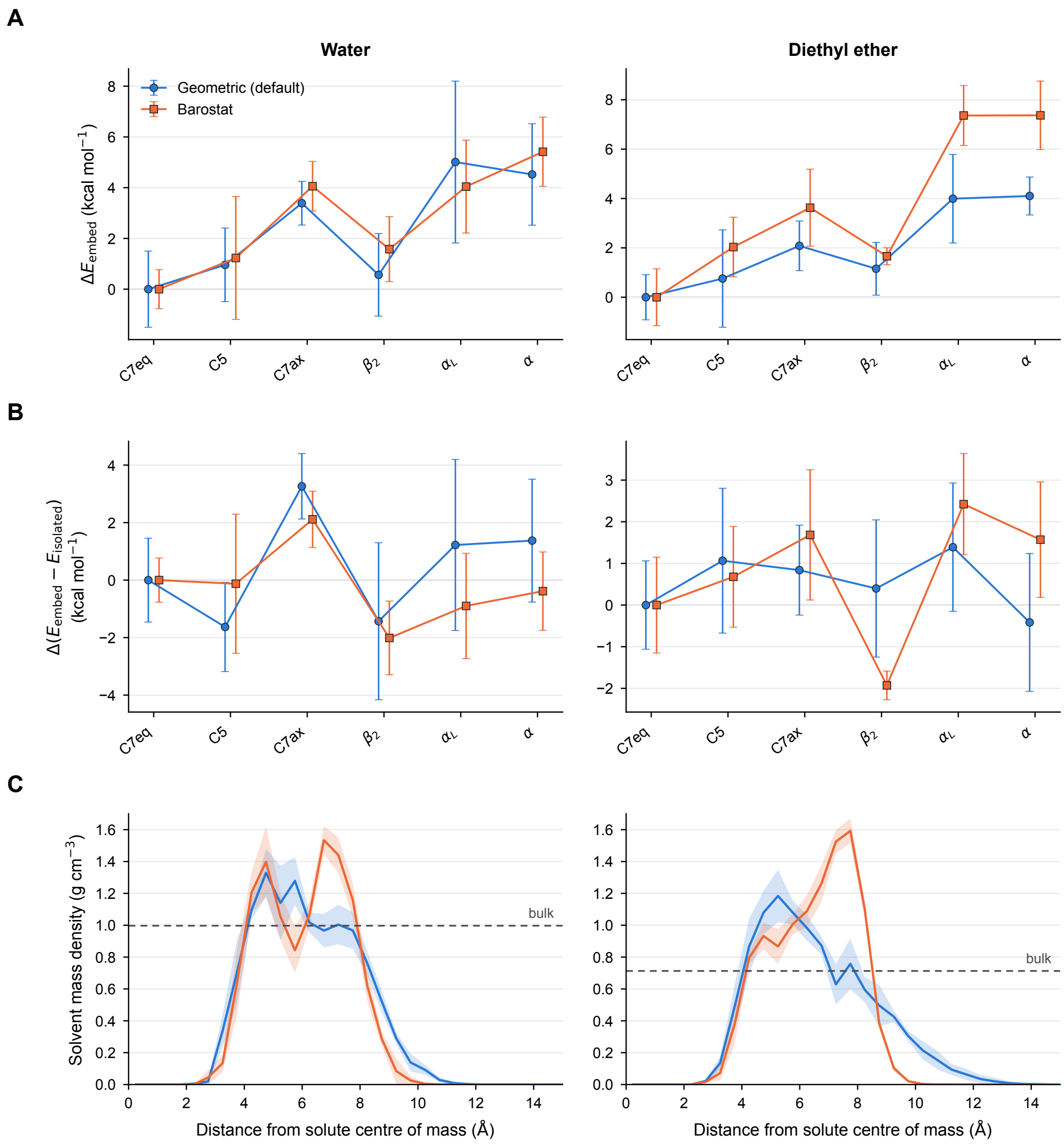

Figure S16: Geometric (default, blue) versus barostat (orange) microsolvation of alanine dipeptide in water (left) and diethyl ether (right), SO3LR-1 on each builder's own geometries (five replicas per conformer, mean ± SD). (A) Relative embedded solute energy $\Delta E_{\mathrm{embed}}$, referenced to C7eq. (B) Relative solvent-induced shift $\Delta(E_{\mathrm{embed}} - E_{\mathrm{isolated}})$ with the energy of the isolated solute at the same geometry subtracted. (C) Solvent mass-density profile around the solute centre of mass (mean of the replica-averaged profiles, band = SD over replicas; bulk density dashed).

Table S34: Compactness of the microsolvated clusters for the two builders (mean over replicas, replica SD in parentheses): number of solvent molecules N_{solv} (range over replicas), radius of gyration R_g, effective density ρ_{eff} (uniform sphere of equal R_g; relative measure only), largest solvent-atom distance R_{max} from the solute centre of mass, and number of first-shell solvent molecules (any atom within 3.5 Å of the solute).

Solvent	Build	N_{solv}	R_g (Å)	ρ_{eff} (g cm^{-3})	R_{max} (Å)	first-shell molecules
Water	Geometric	100	6.94 (0.06)	1.07 (0.03)	11.2 (0.4)	37.1 (1.0)
	Barostat	91–100	6.61 (0.08)	1.18 (0.02)	9.9 (0.4)	36.2 (1.5)
Diethyl ether	Geometric	25	7.60 (0.28)	0.85 (0.09)	13.4 (0.8)	13.8 (1.0)
	Barostat	25	6.92 (0.03)	1.11 (0.01)	10.1 (0.1)	17.9 (0.6)

Table S35: Relative embedded solute energies (kcal mol^{-1}, SO3LR-1, mean over five replicas with the replica SD in parentheses) of alanine dipeptide for the two builders, referenced to C7eq. Upper block: ΔE_{embed}; lower block: the same quantity with the energy of the isolated solute at the same geometry subtracted. The barostat builder keeps the solute frozen at the benchmark geometry (for the isolated solute, α is +5.8 kcal mol^{-1} above C7eq), the geometric builder relaxes it. The geometric clusters were built from the earlier reference geometry.

Solvent	Build	C7eq	C5	C7ax	β_2	α_L	α
ΔE_{embed}							
Water	Geometric	0.0 (1.5)	1.0 (1.5)	3.4 (0.9)	0.6 (1.6)	5.0 (3.2)	4.5 (2.0)
	Barostat	0.0 (0.8)	1.2 (2.4)	4.1 (1.0)	1.6 (1.3)	4.0 (1.8)	5.4 (1.4)
Diethyl ether	Geometric	0.0 (0.9)	0.8 (2.0)	2.1 (1.0)	1.2 (1.1)	4.0 (1.8)	4.1 (0.8)
	Barostat	0.0 (1.2)	2.0 (1.2)	3.6 (1.6)	1.7 (0.3)	7.4 (1.2)	7.4 (1.4)
$\Delta(E_{\text{embed}} - E_{\text{isolated}})$							
Water	Geometric	0.0 (1.5)	-1.6 (1.6)	3.3 (1.1)	-1.4 (2.7)	1.2 (3.0)	1.4 (2.1)
	Barostat	0.0 (0.8)	-0.1 (2.4)	2.1 (1.0)	-2.0 (1.3)	-0.9 (1.8)	-0.4 (1.4)
Diethyl ether	Geometric	0.0 (1.1)	1.1 (1.7)	0.8 (1.1)	0.4 (1.6)	1.4 (1.5)	-0.4 (1.7)
	Barostat	0.0 (1.2)	0.7 (1.2)	1.7 (1.6)	-1.9 (0.3)	2.4 (1.2)	1.6 (1.4)

S14 2-Methoxycyclohexanone: model comparison

This section collects the per-model data underlying the 2-methoxycyclohexanone panels of the main text. Relative conformer energies across the benchmarked potentials (SO3LR-1; SO3LR-2-S/M/L; MACE-OFF23 S/M; MACE-OFF24; MACE-OMol) in the gas phase, CCl_4 and CH_3CN are compared with the B3LYP Onsager reference of Freitas *et al.* [29] in Table S36; the C=O band relative intensities of the SO3LR models against the experimental component ratios are given in Table S37; and the agreement of every potential with the PBE0+MBD reference on the explicit clusters (mean-centered total-cluster energies, MAE and SD per solvent) is summarized in Table S38. SO3LR-1 anti-correlates with PBE0+MBD in CCl_4 because of a poor description of the chlorinated solvent (reflected in the large SD in Table S38 and the wide violin in the main text); its embedded solute energies, and hence the conformer energetics, are not affected.

Table S36: Relative conformer energies of 2-methoxycyclohexanone (kcal mol^{-1}, referenced to the mean energy of the most stable chair conformer in each environment) from single-point embedded energies of the benchmarked potentials, compared with the B3LYP/6-311+G(d,p) Onsager reference of Freitas *et al.* [29]. Solvated values are means over five microsolvation replicas with the standard deviation in parentheses; SD is evaluated from replica energies referenced to the per-replica conformer mean, so the reference conformer also carries an uncertainty. Gas-phase values have no replicas. In CH_3CN, MACE-OFF24 places eq-g^- (rather than eq-g^+) lowest, so all its values are referenced to that conformer.

Method	eq-g^+	ax-g^+	eq-anti	ax-g^-	eq-g^-
Gas phase					
B3LYP (ref.)	0.02	0.00	2.67	1.42	2.52
SO3LR-1	0.00	0.37	1.22	1.49	2.15
SO3LR-2-S	0.34	0.00	1.23	1.41	2.02
SO3LR-2-M	0.36	0.00	1.97	1.48	2.35
SO3LR-2-L	0.82	0.00	2.59	1.41	3.25
MACE-OFF23 S	0.00	0.35	1.49	1.11	2.79
MACE-OFF23 M	0.16	0.00	1.76	1.19	2.66
MACE-OFF24	0.28	0.00	1.51	0.99	2.59
MACE-OMol	0.34	0.00	2.31	1.65	2.86
CCl_4					
B3LYP (ref.)	0.00	0.20	2.55	1.42	2.09
SO3LR-1	0.00 (0.9)	1.75 (1.1)	2.02 (0.6)	2.94 (1.2)	2.36 (1.1)
SO3LR-2-S	0.00 (0.6)	1.22 (0.3)	1.19 (0.5)	2.40 (1.3)	0.83 (0.3)
SO3LR-2-M	0.00 (0.9)	2.44 (0.6)	0.51 (0.2)	2.85 (1.1)	1.09 (0.4)
SO3LR-2-L	0.00 (0.4)	0.39 (0.8)	0.73 (0.5)	1.21 (1.3)	1.26 (0.5)
MACE-OFF23 S	0.00 (0.5)	2.13 (0.8)	0.85 (0.7)	1.74 (1.1)	1.27 (1.0)
MACE-OFF23 M	0.00 (0.5)	1.47 (0.7)	0.13 (0.6)	1.68 (1.2)	0.75 (0.7)
MACE-OFF24	0.00 (0.4)	0.99 (0.7)	0.87 (0.3)	1.21 (0.7)	1.68 (0.6)
MACE-OMol	0.00 (0.8)	0.69 (0.9)	1.24 (0.9)	1.68 (0.8)	0.63 (0.5)
CH_3CN					
B3LYP (ref.)	0.00	0.48	2.39	1.44	1.52
SO3LR-1	0.00 (0.9)	2.55 (1.1)	1.63 (1.0)	3.58 (1.1)	0.59 (0.9)
SO3LR-2-S	0.00 (0.5)	2.30 (0.8)	1.23 (0.9)	2.74 (0.6)	0.44 (0.5)
SO3LR-2-M	0.00 (0.7)	1.49 (1.0)	0.74 (0.7)	1.40 (0.4)	0.66 (0.3)
SO3LR-2-L	0.00 (0.6)	1.50 (0.7)	1.15 (0.7)	2.37 (0.3)	0.36 (0.4)
MACE-OFF23 S	0.00 (1.0)	2.34 (0.9)	2.43 (1.2)	2.53 (1.3)	0.22 (0.4)
MACE-OFF23 M	0.00 (0.7)	3.10 (0.7)	2.21 (0.8)	3.99 (0.8)	0.18 (0.5)
MACE-OFF24	0.09 (0.7)	3.18 (0.7)	1.14 (1.3)	4.28 (0.8)	0.00 (0.5)
MACE-OMol	0.00 (0.6)	3.03 (0.7)	2.36 (1.0)	4.11 (0.9)	0.37 (0.5)

Table S37: C=O stretching band relative intensities $I/\sum I$ of 2-methoxycyclohexanone from the SO3LR models (population-weighted intrinsic carbonyl intensities over the experimentally resolved bands), compared with the experimental ratios reported by Freitas *et al.* [29]. A dash marks bands not resolved experimentally.

Method	ax-g^+	eq-g^+	ax-g^-	eq-g^-
CCl_4				
Exp. (ref.)	0.08	0.84	0.08	–
SO3LR-1	0.05	0.95	0.01	–
SO3LR-2-S	0.12	0.86	0.02	–
SO3LR-2-M	0.02	0.98	0.01	–
SO3LR-2-L	0.37	0.53	0.10	–
CH_3CN				
Exp. (ref.)	0.06	0.89	0.01	0.04
SO3LR-1	0.01	0.78	0.00	0.22
SO3LR-2-S	0.01	0.66	0.01	0.32
SO3LR-2-M	0.07	0.55	0.09	0.30
SO3LR-2-L	0.04	0.62	0.01	0.33

Table S38: Agreement of each potential with the PBE0+MBD reference for the explicit 2-methoxycyclohexanone clusters: mean absolute error (MAE) and standard deviation (SD) of the mean-centered total-cluster energy over 80 clusters (16 conformers × 5 replicas) per solvent. SO3LR-1 anti-correlates with PBE0+MBD in CCl_4 owing to poor description of the chlorinated solvent; its embedded solute energies are unaffected.

Method	MAE_{CCl_4}	SD_{CCl_4}	MAE_{CH_3CN}	SD_{CH_3CN}
SO3LR-1	9.16	11.59	2.62	3.14
SO3LR-2-S	3.19	3.85	1.45	1.85
SO3LR-2-M	2.85	3.50	1.46	1.71
SO3LR-2-L	2.58	3.18	1.46	1.68
MACE-OFF23 S	2.79	3.44	2.65	3.07
MACE-OFF23 M	3.24	4.04	2.23	2.95
MACE-OFF24	2.53	3.16	1.85	2.27
MACE-OMol	2.11	2.63	0.69	0.88

S15 Backbone conformational preferences of capped alanine peptides

To probe how each machine-learned potential samples peptide backbone conformational space, we generated MARS ensembles for four capped alanine peptides of increasing length—ACE–$(\mathrm{Ala})_5$–NME (Ala5), and the C-terminally lysine-capped ACE–$(\mathrm{Ala})_5$–LysH^+ ($\mathrm{Ala5LysH}^+$), ACE–$(\mathrm{Ala})_{10}$–LysH^+ ($\mathrm{Ala10LysH}^+$), and ACE–$(\mathrm{Ala})_{15}$–LysH^+ ($\mathrm{Ala15LysH}^+$)—with eight potentials: SO3LR-1, the three SO3LR-2-S/M/L, MACE-OFF23 small and medium, MACE-OFF24, and MACE-OMol. For every conformer of every ensemble, the backbone dihedral angles $\phi_i = (\mathrm{C}_{i-1}, \mathrm{N}_i, \mathrm{C}\alpha_i, \mathrm{C}_i)$ and $\psi_i = (\mathrm{N}_i, \mathrm{C}\alpha_i, \mathrm{C}_i, \mathrm{N}_{i+1})$ were computed for each alanine residue, and the residue identity of every atom was assigned purely from connectivity.

Figure S17 collects the resulting Ramachandran maps as a 8×4 grid (one row per potential, one column per peptide). Points are coloured by the relative energy $\Delta E = E - E_{\mathrm{min}}$ of their conformer on a single global scale shared by all panels, and the global energy minimum of each ensemble is marked with a star. The canonical right-handed α-helix and β-sheet regions are overlaid for reference. All seven potentials concentrate the low-energy conformers in the upper-left (β/PPII) and lower-left (α_{R}) basins expected for L-alanine, with the longer lysine-capped peptides showing a progressively tighter clustering around the α_{R} region. The MACE-OFF23 medium and MACE-OMol ensembles for the two longest peptides ($\mathrm{Ala10LysH}^+$ and $\mathrm{Ala15LysH}^+$) were not converged due to time or memory limits and are shown as empty panels; the MACE-OFF24 runs for $\mathrm{Ala5LysH}^+$ and $\mathrm{Ala15LysH}^+$ stopped with GPU out-of-memory errors before the final ensemble was written and are likewise empty.

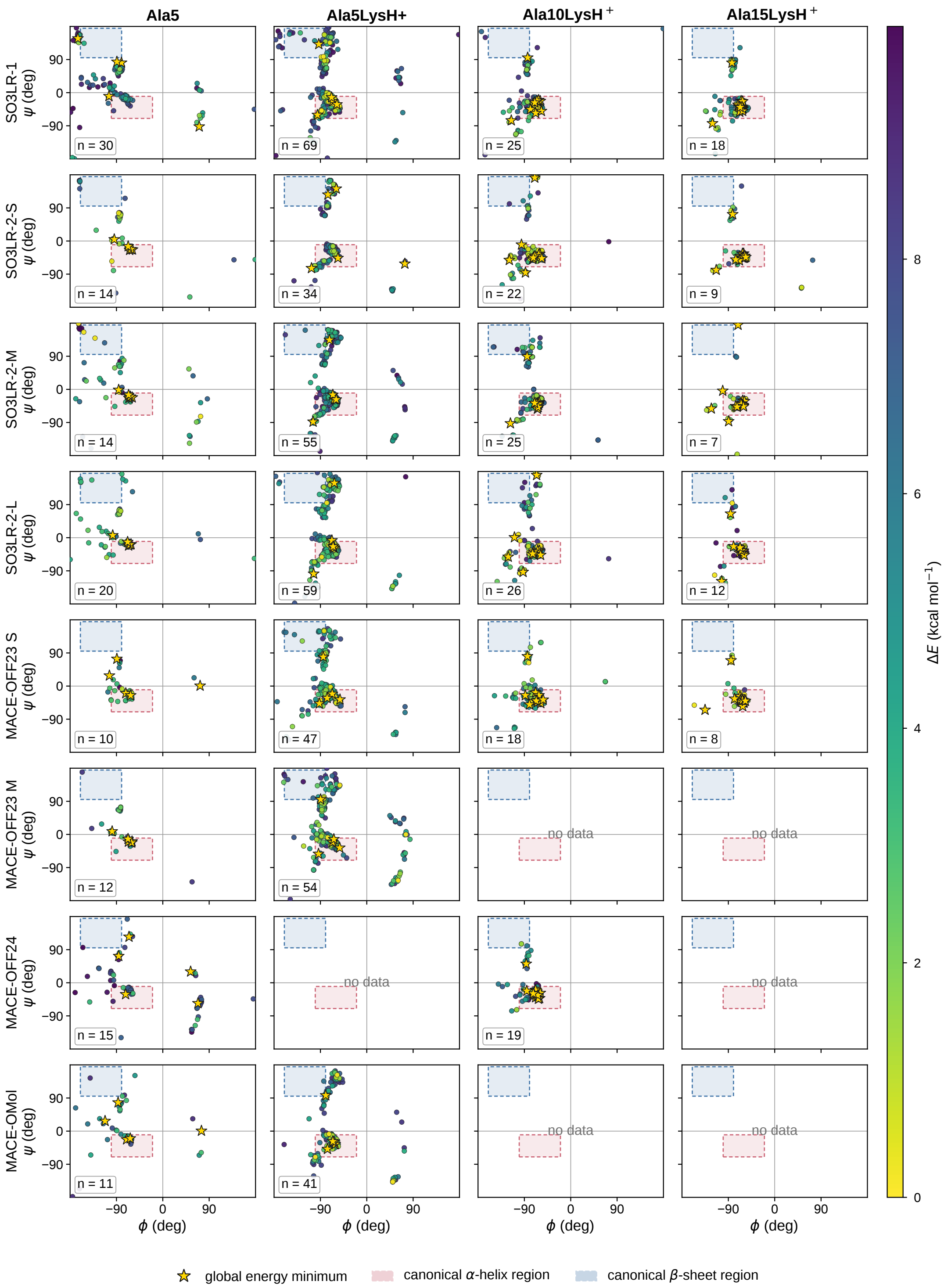

Figure S17: Backbone Ramachandran maps (ϕ vs. ψ) of the MARS-generated conformer ensembles for four capped alanine peptides (columns) sampled with eight machine-learned potentials (rows). Each point is one alanine residue in one conformer, coloured by the conformer relative energy $\Delta E = E - E_{\min}$ on a global scale shared across all panels; the gold star marks the global energy minimum of each ensemble and n is the number of conformers. Shaded dashed boxes indicate the canonical right-handed α-helix and β-sheet regions. Empty panels correspond to non-convergent runs.

S16 Proline/β-cyclodextrin inclusion: model comparison

To assess how strongly the proline/β-cyclodextrin results depend on the choice of machine-learned potential, we repeated the analysis across the MARS potential family. Starting from the same SO3LR-1 conformer ensembles used in the main text (no re-optimization), we recomputed, as single points on the common lowest-energy geometry, the charge-partitioned inclusion (dimer) interaction energy $\Delta E_{\text{int}} = E_{\text{complex}} - E_{\beta\text{-CD}} - E_{\text{guest}}$ for neutral, cationic and anionic proline, with SO3LR-1, the three SO3LR-2 models (S/M/L) and four MACE models (MACE-OFF23 small and medium, MACE-OFF24, and MACE-OMol. For SO3LR this uses the same dimer protocol as the main text—the bound complex versus a frame with the guest translated 10^4 Å away, evaluated in a single pass so that the charge equilibration assigns the correct per-fragment charge (long-range cutoff 1000 Å)—whereas for MACE, which has no long-range charge equilibration, the fragments are separated and each carries its own total charge. Harmonic IR spectra were recomputed with SO3LR-1 and the three SO3LR-2 models (finite-difference Hessian, `float64`) for all seven species and 300 K Boltzmann-averaged over each ensemble. MACE-OFF23 and MACE-OFF24 are not charge-aware, so for the charged guests the guest charge is ignored (flagged in Figure S18); SO3LR-1 and SO3LR-2 and MACE-OMol honour it.

The neutral interaction energy is remarkably model-robust, spanning only −19 to −27 kcal/mol across all eight potentials (Figure S18), with SO3LR-1 giving −25.7 kcal/mol as in the main text. The charged complexes are more model-dependent but internally consistent among the charge-aware potentials: SO3LR-1/SO3LR-2 and MACE-OMol predict strong charge-assisted binding of −67 to −74 kcal/mol for the cation and −99 to −112 kcal/mol for the anion (SO3LR-1: −69.9 and −112.5 kcal/mol, matching the main text), consistent with hydrogen bonding to the β-CD hydroxyl rim. The charge-unaware MACE-OFF23 and MACE-OFF24 models markedly underestimate both charged complexes (−44 to −52 kcal/mol for the cation and −71 to −76 kcal/mol for the anion) because they treat the guest as neutral. The Boltzmann-averaged IR spectra (Figure S19) are mutually consistent across the SO3LR versions for all seven species, reproducing the same band envelopes—in particular the dominant β-CD O–H manifold and the guest marker bands—with only minor version-dependent shifts in peak position. Taken together, the inclusion energetics and the vibrational signatures are transferable across the MARS potential family, provided a charge-aware potential is used for the charged guests.

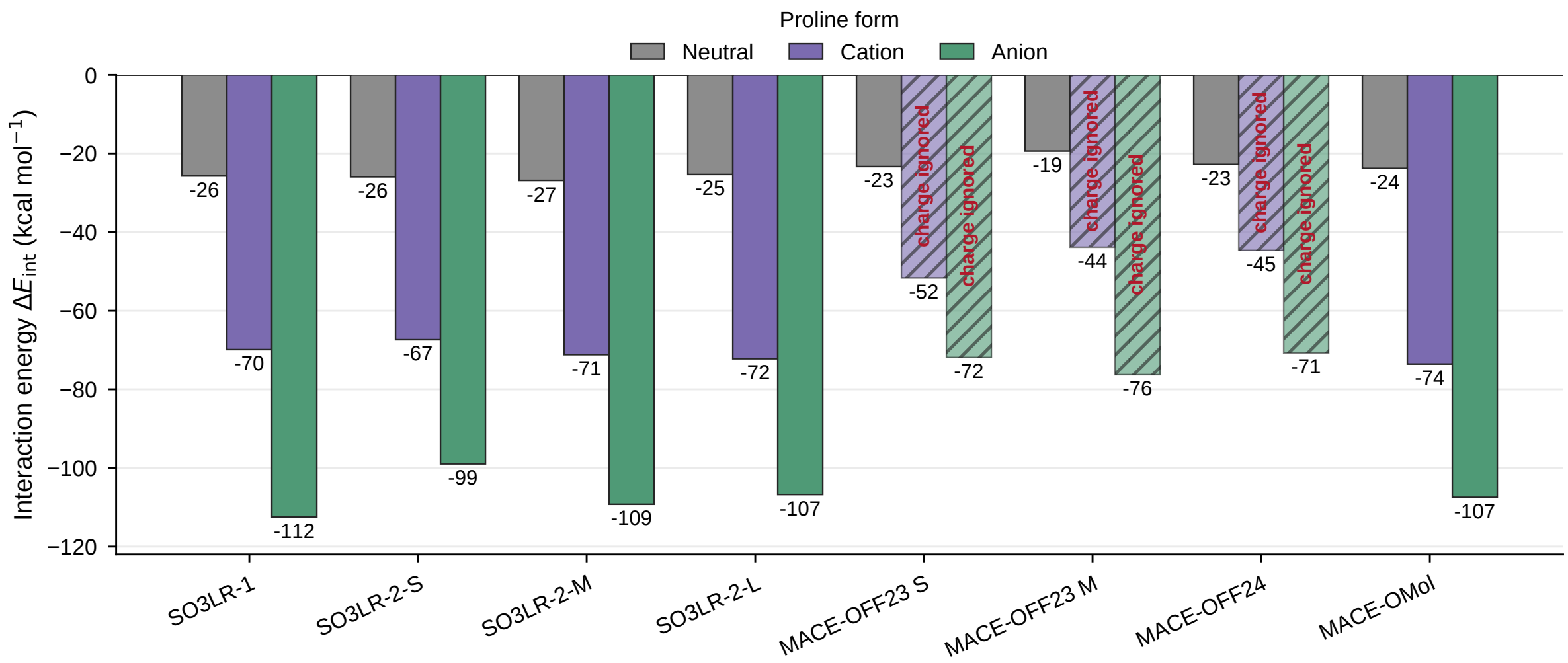

Figure S18: Charge-partitioned inclusion (dimer) interaction energy $\Delta E_{\text{int}} = E_{\text{complex}} - E_{\beta\text{-CD}} - E_{\text{guest}}$ of neutral, cationic and anionic proline in β-cyclodextrin, as single points on the common SO3LR-1 lowest-energy geometry for each MARS potential (values in kcal/mol). SO3LR uses the dimer protocol (bound complex vs. the guest translated 10^4 Å away, with per-fragment charge equilibration at a 1000 Å long-range cutoff); MACE uses separated fragments each carrying its own total charge. MACE-OFF23 and MACE-OFF24 are not charge-aware and ignore the guest charge for the ions (hatched, marked). The neutral value is model-robust; among the charge-aware potentials (SO3LR-1/SO3LR-2 and MACE-OMol) the charged complexes are mutually consistent, and SO3LR-1 reproduces the main-text values (-25.7, -69.9 and -112.5 kcal/mol).

S17 Redundant Internal Coordinates

The optimizers of Section S2 work in Cartesian coordinates. Quantum-chemistry geometry optimizers conventionally use redundant internal coordinates (RIC) together with a chemically informed initial Hessian, which is well established to reduce the number of optimization steps [30, 31, 32, 33]. Both are implemented in MARS as an opt-in alternative on `mars optimize` (`--coords internal`, `--init-hessian`), in order to quantify what they contribute for machine-learned force fields, where a gradient costs milliseconds rather than a self-consistent-field solution and the linear algebra of the coordinate transformation is therefore no longer negligible against it.

The coordinate set comprises bond stretches, valence bends, linear-bend pairs, proper torsions and Wilson out-of-plane bends, with translation–rotation internal coordinates (TRIC) [34] adding rigid-body coordinates per fragment for multi-fragment systems. Steps are taken by rational function optimization and back-transformed to Cartesian displacements by the iterative Pulay–Fogarasi scheme [30]. The initial Hessian follows the Lindh model [6], whose damping is defined for any atom pair and so also covers interfragment and hydrogen-bonded contacts, unlike the bonded-topology models of Schlegel [35] and Fischer–Almlöf [36]. Convergence is tested on the Cartesian maximum force component, identically to the Cartesian optimizers, so that f_{max} stays comparable across coordinate systems.

Three arms were compared on the curated conformer-energy benchmark set of Section S9 and Table 1 of the main text (36 molecules, 14–116 atoms, 454 conformers; SO3LR-1, double precision, one NVIDIA V100) at $f_{\text{max}} = 0.01$ eV/Å, and on the complete conformational-search workflow for the same 36 molecules. On the molecules used here, `jit` compilation cost is 18–20 s. Step counts are reported for the protocol in which every conformer is optimized in its own call; wall times and memory are reported for the batched protocol, which is how the workflow

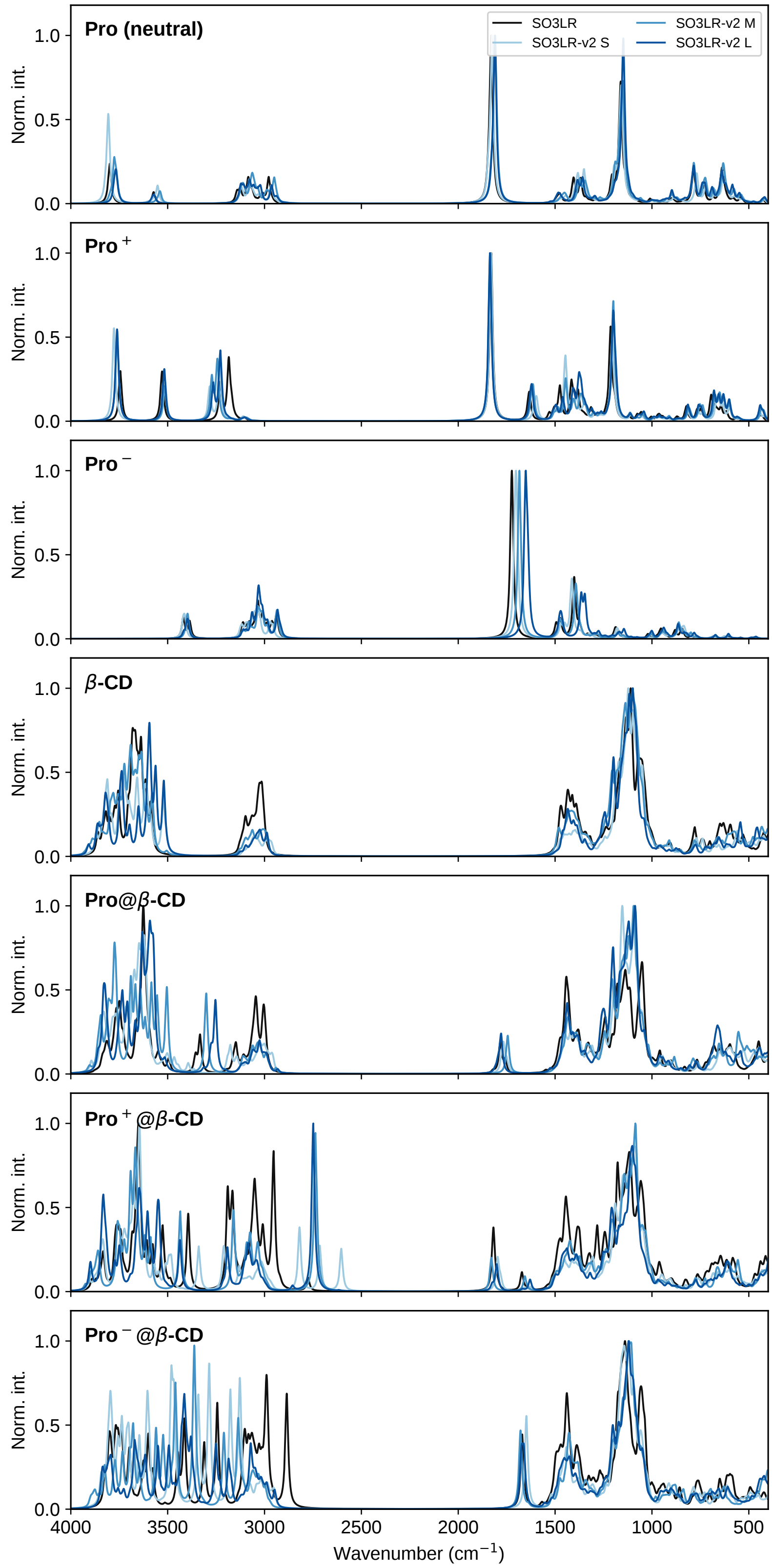

Figure S19: 300 K Boltzmann-averaged harmonic IR spectra of free proline (neutral, cationic and anionic), β-cyclodextrin, and the three inclusion complexes, computed with SO3LR-1 and the three SO3LR-2 models (S/M/L) on the common SO3LR-1 conformer ensembles (finite-difference Hessian, unscaled harmonic frequencies, Lorentzian broadening, FWHM = 18 cm^{-1}, intensities normalized per panel). The band envelopes—including the dominant β-CD O–H manifold and the guest marker bands—are reproduced consistently across SO3LR versions.

optimizes an ensemble. Internal coordinates with the Lindh Hessian then converge in a median of 10 steps against 311, with a step that is "cheaper", 24 against 81 ms, so that one batched optimization takes 0.37 s against 73 s

However, RIC has some limitations to take into account. **Precision:** internal coordinates are unusable in single precision, the back-transformation residual leaves a spurious force larger than a tight threshold, so MARS refuses them without `--float64`. **Memory:** the RFO stepper carries dense matrices of side n_{int} per structure on top of the doubling double precision imposes on the potential itself, which means fewer structures per batch, more passes. **A shared coordinate set:** a batch needs one coordinate specification, and for 5 of the 36 molecules the bond detection assigns different bonds to different conformers, so it falls back to Cartesian coordinates; those molecules are excluded below.

The complete conformational search nevertheless takes the same time in all three arms, because it is sampling-bound; the geometry optimization stage is under three per cent of its wall time, so Cartesian L-BFGS remains its default.

Table S39: Redundant internal coordinates versus the Cartesian L-BFGS default, on the curated conformer-energy benchmark set of Section S9 (SO3LR-1, double precision, one V100, $f_{\max} = 0.01$ eV/Å). Steps and the number of converged structures are medians and totals over the 454 reference conformers optimized one per call. The cost of a step excludes the compilation, which is listed separately; both are medians over the 31 molecules for which internal coordinates were used. The wall time in geometry optimization is the median over those 31 molecules of one batched optimization of all conformers of a molecule, and the additional memory is the extra peak GPU memory per structure for the largest system (YIVNOG, 116 atoms). The conformer-search wall time is the median over the 36 molecules and the ratio is the geometric mean of the per-molecule ratios against the Cartesian baseline.

		Convergence		Geometry optimization			Search
Coordinates	$\mathbf{H}_0$	Steps	ms/step	compile (s)	wall (s)	Δmem (MB)	ratio
Cartesian	identity (L-BFGS)	311	81	20.1	73.3	—	1.000
Internal	identity	156	24	18.4	0.74	+16.3	0.994
Internal	Lindh	**10**	24	19.2	**0.37**	+16.4	0.986

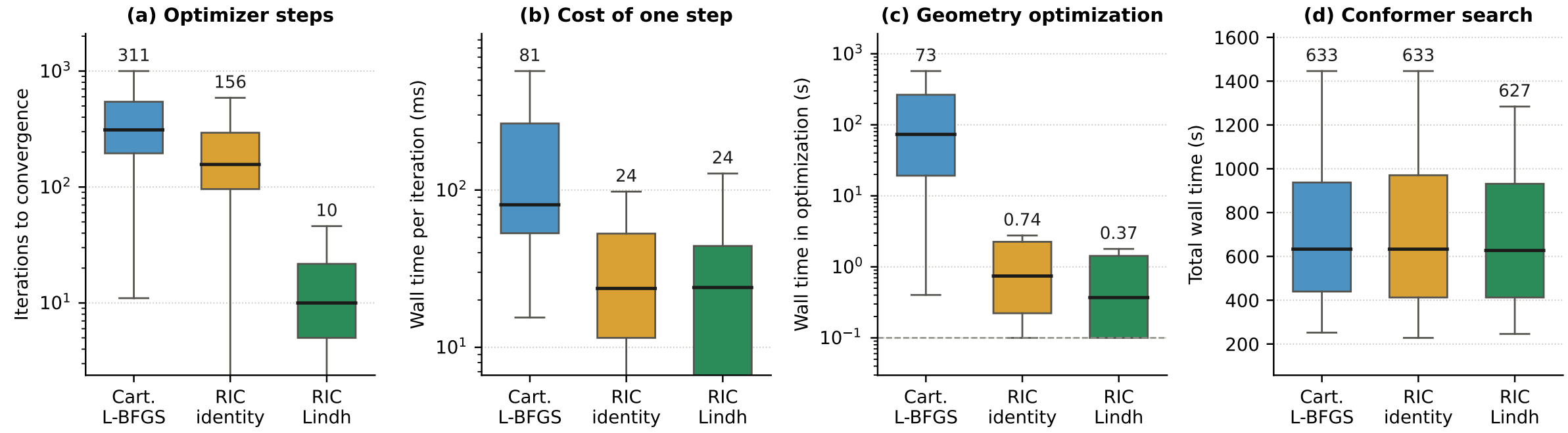

Figure S20: Redundant internal coordinates versus Cartesian L-BFGS with SO3LR-1 at $f_{\max} = 0.01$ eV/Å, on the curated conformer-energy benchmark set. (a) Optimizer steps to convergence, over the 454 reference conformers optimized one per call. (b) Wall time of one optimizer step, with the compilation excluded, over the 31 molecules for which internal coordinates were used. (c) Wall time spent in geometry optimization when all conformers of a molecule are optimized in one batched call, over the same 31 molecules; the dashed line marks 0.1 s, below which the subtraction of the fixed cost no longer resolves the optimization and the points are drawn at the floor. (d) Total wall time of the complete conformational-search workflow, over the 36 molecules. Boxes give the interquartile range, whiskers 1.5×IQR, and medians are annotated.

S18 Overlap between benchmark molecules and machine-learning training data

To determine whether train–test leakage could contribute to the reported performance, we searched for overlaps between the molecules used in our benchmarks and case studies and those contained in the training datasets of the machine-learned potentials examined here. The models considered were SO3LR [8], the unpublished SO3LR-2-S/M/L variants, MACE-OFF23 and MACE-OFF24 [11], and MACE-OMol, which is trained on the Open Molecules 2025 dataset [37]. For SO3LR-2 and the MACE-OMol/OFF models, we estimated overlap using publicly accessible datasets that match the reported training compositions of these models. Any conclusions regarding data overlap should therefore be interpreted in light of this constraint, which is discussed further below.

S18.1 Scope and definition of overlap

We quantify overlap at the *molecule level*, i.e., two entries are considered overlapping if they correspond to the same molecular graph irrespective of their conformation. This is the relevant definition for the present study because all benchmark targets are *relative* quantities, including conformer energy differences, geometry-retention metrics, and harmonic vibrational spectra. These quantities are evaluated against *independent* high-level references (PBE0+MBD, ωB97M-V, DLPNO-CCSD(T), or experiment) rather than against training labels that may have been seen by a given potential.

Furthermore, the benchmark geometries are generated specifically for this work, either from SO3LR-derived conformer ensembles or from the reference DFT optimizations. Exact agreement between a benchmark structure and a training-set conformation is therefore highly unlikely by construction. Consequently, we do not attempt an explicit conformer-level comparison and instead report molecule-level overlap, which provides a conservative *upper bound* on any possible conformer-level leakage.

Molecular identity is defined through the standard RDKit InChIKey. Overlap is determined using only the connectivity block (the first 14 characters), thereby making the matching procedure insensitive to molecular conformation and numerical variations in three-dimensional

coordinates. For non-covalent assemblies, such as dimers, ion pairs, and microsolvation clusters, structures are decomposed into their covalently connected fragments and each fragment is matched separately ("component-level" matching), consistent with the fragment-based definition of the benchmark interaction energies. Under this protocol, the benchmark contains 4568 unique molecules.

S18.2 Method

Given the large size of the training datasets relative to the benchmark collection, overlap analysis was carried out using a streaming search procedure. All benchmark molecules were first stored in an in-memory query index, after which each training dataset was examined in a single sequential pass. For every training structure, a canonical molecular identifier was generated and tested against the benchmark index. To improve efficiency, costly molecular-graph reconstruction was deferred until a structure satisfied the preliminary matching criteria, meaning that only a small fraction of entries required full processing. The methodology is described in three steps: assembly of the benchmark index, definition of the canonical identifier, and execution of the streaming overlap analysis.

S18.2.1 Benchmark query index

The benchmark index is constructed from the exported benchmark structures provided with this Supporting Information as multi-frame XYZ files, where each frame corresponds to a benchmarked conformer and carries its associated dataset, subset, and system identifiers. For each distinct *system*, a single representative structure is selected and converted into a molecular identity as described in Section S18.2.2.

Non-covalent systems, including the NCI dimers, QUID dimers, and microsolvation clusters, are treated at the component level. Each structure is therefore decomposed into its covalently connected fragments, and every fragment is indexed as an independent molecule. This choice is consistent with the definition of the benchmark targets, which are reported per component (e.g. interaction energies or solute-specific energetics). For microsolvation clusters, fragment identities are collected across all exported frames to ensure that every solvent species present in the dataset, including water, diethyl ether, CH_3CN, and CCl_4, is represented in the index.

Molecular identities for the TorsionNet500 benchmark are obtained directly from the canonical SMILES strings supplied in the reference dataset. For all other benchmark sets, molecular identities are derived from the corresponding three-dimensional structures. Molecules occurring in multiple benchmark collections, such as systems shared between the IR and torsional benchmarks, are merged through de-duplication on the canonical molecular key. This procedure yields a total of **4568** unique benchmark molecules.

The resulting index is stored as a hierarchical mapping of the form $\{\text{formula} \rightarrow \{\text{key} \rightarrow \text{benchmark molecule}\}\}$, together with the complete set of benchmark molecular formulas. Each indexed molecule retains the list of associated `(dataset, subset, system)` identifiers, enabling overlap statistics to be attributed to individual benchmark collections.

S18.2.2 Canonical molecular key

Molecular identity is defined using the standard **InChIKey** generated with RDKit/InChI. Unless stated otherwise, two structures are considered identical when they share the **connectivity block** of the InChIKey (the first 14 characters). This portion of the identifier encodes molecular constitution through the connectivity, molecular formula, and fixed-hydrogen layers, making the matching procedure invariant to atom ordering, molecular conformation, and minor variations in three-dimensional coordinates while remaining sensitive to changes in chemical composition or bonding. The complete InChIKey is retained for traceability.

As a supplementary analysis, we also evaluate overlap using a protonation- and tautomer-insensitive representation. Benchmark molecules are first normalized using the RDKit `Uncharger` and then converted to the canonical form returned by the `TautomerEnumerator`. Matches obtained from these normalized identifiers are recorded as secondary ("loose") overlaps, capturing cases where molecules differ only by protonation state or tautomeric form (e.g. a carboxylic acid and its corresponding carboxylate). Throughout this work, however, overlap statistics are reported primarily using the stricter connectivity-block criterion.

S18.2.3 Streaming semi-join

Each training dataset is read once (Algorithm S21). Every training entry follows one of two paths to a key:

- **Identity-based pathway.** For datasets that explicitly provide chemical identities, such as SMILES strings (including atom-mapped variants) or molecular connection tables stored in SDF files, molecular identifiers are obtained directly from the supplied representation without reference to atomic coordinates. Atom-mapping annotations are removed prior to processing. Structures whose SMILES strings cannot be parsed are excluded from the analysis and recorded separately; the failure rate is below 0.2%.

- **Geometry-based pathway.** For datasets containing only atomic numbers and Cartesian coordinates, molecular identification is performed through a multi-stage filtering procedure. First, the Hill formula is computed directly from the atomic composition, an $O(N_{\text{atoms}})$ operation that does not require bond perception. This formula is then compared against the set of formulas present in the benchmark index. Only structures sharing a benchmark formula proceed to the next stage, where the covalent graph is reconstructed from the coordinates using RDKit `DetermineBonds`. Because bond assignment depends on the molecular charge, the stored charge is attempted first, followed by neutral and ± 1 charge states when necessary. The resulting molecular graph is subsequently converted into an InChIKey for overlap testing

  The formula-based pre-screening eliminates the need for graph reconstruction for the vast majority of training structures. Since benchmark formulas represent only a small subset of all formulas encountered in the training data, this filtering step substantially reduces computational cost and enables efficient single-pass analysis of datasets containing millions of structures.

Training structures containing multiple disconnected species, such as ion pairs or solvated clusters, are decomposed into their covalently connected fragments prior to overlap analysis. Fragmentation is performed by constructing a neighbor graph using a KD-tree and assigning a bond between atoms i and j whenever their separation is smaller than $1.3\,(r_i^{\text{cov}}+r_j^{\text{cov}})$. Connected components are then identified using a union–find procedure, and each fragment is processed independently to generate its molecular key. A fragment is counted as a *strict* overlap when its 14-character InChIKey connectivity block is present in the benchmark index. Matches obtained only through the protonation- and tautomer-normalized representation are recorded separately as *loose* overlaps. For every benchmark molecule, we additionally track the number of matching training structures together with the corresponding dataset, subset, and provenance information. For the largest geometry-only datasets, computational cost is controlled through a per-formula graph-perception limit of 60 distinct geometries. Once this threshold is reached, additional structures sharing the same molecular formula are not processed further. Because this restriction can only prevent the detection of additional overlaps, the reported counts for affected datasets should be regarded as conservative lower bounds.

Algorithm S21 Streaming semi-join for one training dataset

```
Input: benchmark index Q (formula → keys), formula set F
for each entry e in the training dataset (streamed once) do
    if e ships an identity string s then
        k ← InChIKey(Parse(s));   TestAndRecord(k)
    else                                                ▷ geometry only: (Z, R)
        for each covalent fragment (Z_f, R_f) of (Z, R) do
            φ ← HillFormula(Z_f)                        ▷ cheap; no bonds
            if φ ∈ F and per-formula cap not reached then
                m ← DetermineBonds(Z_f, R_f, charge)
                k ← InChIKey(m);   TestAndRecord(k)
            end if
        end for
    end if
end for
```

TestAndRecord(k): if k[:14] $\in Q$ record a strict hit on that benchmark molecule; else if the normalized k matches, record a loose hit.

S18.2.4 Per-dataset readers

The identity- and geometry-based workflows are connected to the individual training datasets through lightweight, format-specific readers (Table S40). Datasets that provide an explicit molecular representation are processed through the identity-based pathway, whereas geometry-only datasets are handled through the geometry-based pathway.

QCML requires a dedicated reader because its public `dft_metadata` release is distributed as sharded TensorFlow-record files. These shards are streamed and parsed directly as protocol-buffer messages, avoiding any TensorFlow dependency. The per-record `chemical_formula` field, whose numerical subscripts are normalized from Unicode to ASCII, is used for the formula-based pre-screening step, while the corresponding `smiles` entry is accessed only for records that pass this filter.

Open Molecules 2025 (OMol25) is stored as ASE database entries serialized as zlib-compressed JSON objects within LMDB shards. Each record provides atomic numbers, Cartesian coordinates, molecular charge, and a `source` label. Alongside overlap detection, the `source` field is aggregated to validate the reported composition of the OMol25 training set.

GEOM is handled through its file naming convention alone. In the local copy used for this study, every conformer file is named by its InChIKey, allowing benchmark molecules to be matched directly against the directory listing without opening or parsing the underlying files.

Table S40: Per-dataset readers. "Identity" = the fast path (a stored SMILES/bond block canonicalized straight to an InChIKey); "geometry" = the funnel path (Hill-formula pre-filter, then bond perception from coordinates). "Scanned" is the number of records/structures actually read.

Dataset	On-disk format	Identity source (path)	Scanned
SPICE v1	HDF5 (per-molecule group)	mapped SMILES (identity)	19,238
SPICE v2	HDF5 (per-molecule group)	mapped SMILES (identity)	113,986
DES (370K+15K)	CSV (dimer rows)	monomer SMILES (identity)	392 uniq. monomers
AQM	Zip → CSV	SMILES (identity)	1,653
QMugs	Tar.gz of per-compound SDFs	SDF bond block (identity)	664,303 compounds
QM7-X	8 xz-compressed HDF5	geometry, 1 opt. conf./mol.	6,950 molecules
GEMS	SQLite (int32/float32 blobs)	geometry (funnel)	2,725,800
SO3LR (dipep., DES15k)	HDF5 (atNUM/atXYZ)	geometry (funnel)	46,563
OMol25	LMDB, zlib+JSON ASE rows	geometry + `source` tag	3.9,M (val+train)
QCML	TFRecord (protobuf shards)	SMILES after formula pre-filter	33,496,171
GEOM	Tar; files named by InChIKey	InChIKey (filename)	16,865 members

Table S41 maps these datasets onto the potentials. All were read from local copies except QCML, streamed from the public `dft_metadata` release. Two coverage limitations are stated explicitly. (i) The local OMol25 copy is the neutral train+validation subset; we scanned all 80 validation shards and 9 of the 10 available training shards directly (∼3.9 million structures; one training shard was truncated on disk and skipped) and, in addition, decomposed OMol25 into its documented constituent sources—confirmed by the `source` tags of the records we read (ANI-2x, Transition1x, GEOM, OrbNet-Denali, SPICE)—each of which is scanned here. The direct OMol25 count is therefore a lower bound and the union over its constituent sources an upper bound; an exhaustive conformer-level scan of the full ∼25M-record release is impractical and, on the strongly distorted OMol25 geometries, less reliable than this provenance decomposition. (ii) The QCell dataset used by SO3LR-2 is not publicly available and could not be scanned, so the SO3LR-2 figures omit any QCell-only molecules.

Table S41: Training datasets scanned for each benchmarked potential. SPICE-dipep. is the SPICE dipeptide subset shipped with the SO3LR training data.

Potential	Training datasets scanned
MACE-OFF23 (S/M)	SPICE v1
MACE-OFF24	SPICE v2
SO3LR-1	SPICE-dipeptides, QM7-X, GEMS, AQM, DES (SO3LR training set)
MACE-OMol	OMol25 (direct scan + constituent sources)
SO3LR-2 (S/M/L)	OMol25, QM7-X, QCML *(QCell not public)*

S18.2.5 Validation

The reliability of the geometry-to-InChIKey conversion used in the geometry-based workflow was assessed on the 500 TorsionNet500 molecules, for which a reference SMILES is shipped alongside the geometry: the InChIKey obtained by perceiving the covalent graph from the 3D coordinates was compared against the InChIKey obtained directly from the reference SMILES. Exact agreement was found for 498 of the 500 molecules (99.6%); the two discrepancies are protonation-state/tautomeric ambiguities, precisely the class of differences absorbed by the normalized-key analysis described above. Additional sanity checks were performed using both positive and negative controls. As expected, benchmark molecules known to be present in reference training datasets were successfully detected: alanine dipeptide in SPICE, small alkanes such as butane in QM7-X, and the amino acids cysteine and serine in the protein-fragment (GEMS) and drug-like (QMugs) datasets. For the negative controls, molecules well outside the molecular-size range of the size-limited small-molecule sets were correctly returned as absent from them: β-cyclodextrin ($C_{42}H_{70}O_{35}$) and the MPCONF196 macrocycles are not found in SPICE, DES, AQM, or QM7-X. Consistent with the breadth of the largest corpora—and not an error—β-cyclodextrin is nonetheless present in OMol25, and a few MPCONF196 macrocycles appear in OMol25/QMugs (Table S42); these are the only large case-study species detected anywhere. Together, these results indicate that the workflow correctly identifies both the presence and the absence of benchmark molecules in the training datasets.

S18.3 Results

Table S42: Molecule-level (constitutional) overlap between each benchmark set and each locally scanned training dataset. Entries are the number of *unique* benchmark molecules (component-level InChIKey, connectivity layer) that also occur in the training set; the "Unique" column (col. 2) is the number of distinct molecules in that benchmark set. GEMS is a training source of SO3LR-1; QCML, OMol25, QM7-X of SO3LR-2; SPICE v1/v2 of MACE-OFF23/24; GEOM is shown as an OMol25 constituent (provenance) source, not a stand-alone model training set.

Benchmark set	Unique	SPICE v1	SPICE v2	DES	AQM	QMugs	QM7-X	GEMS	SPICE-dipep.	OMol25	QCML	GEOM
Conformational benchmark	36	5	5	6	4	7	9	8	4	15	11	18
Non-covalent benchmark (monomers)	16	10	10	10	3	4	4	11	9	11	8	11
Infrared benchmark (NIST35)	4031	150	162	141	92	348	446	148	85	818	712	1507
TorsionNet500	495	2	2	2	0	19	7	5	0	18	15	9
CCCBDB torsion scans	34	23	24	23	9	3	12	14	21	30	29	25
QUID dimers (monomers)	11	3	3	2	10	8	0	2	2	3	1	2
Case: capped alanine peptides	3	0	0	0	0	0	0	0	0	0	0	0
Case: proline/β-cyclodextrin	2	0	0	0	0	1	0	1	0	2	0	1
Case: alanine dipeptide microsolvation	3	3	3	3	2	0	1	2	2	3	2	1
Case: 2-methoxycyclohexanone	3	2	2	1	1	1	1	0	1	2	2	2
Case: methanol IR demo	1	1	1	1	1	0	0	1	1	1	1	1
**All (unique)**	4568	162	174	152	107	383	451	159	92	860	739	1527

Table S43: Molecule-level overlap aggregated per benchmarked potential (union of the training datasets available to each model). Values are unique benchmark molecules present in the training data of each model (out of the total unique count), with the percentage of that benchmark set in parentheses.

Benchmark set	Unique	MACE-OFF23 (S/M)	MACE-OFF24	MACE-OMol	SO3LR-1	SO3LR-2 (S/M/L)
Conformational benchmark	36	5 (13.9%)	5 (13.9%)	15 (41.7%)	15 (41.7%)	20 (55.6%)
Non-covalent benchmark (monomers)	16	10 (62.5%)	10 (62.5%)	11 (68.8%)	12 (75.0%)	11 (68.8%)
Infrared benchmark (NIST35)	4031	150 (3.7%)	162 (4.0%)	818 (20.3%)	580 (14.4%)	1291 (32.0%)
TorsionNet500	495	2 (0.4%)	2 (0.4%)	18 (3.6%)	9 (1.8%)	31 (6.3%)
CCCBDB torsion scans	34	23 (67.6%)	24 (70.6%)	30 (88.2%)	25 (73.5%)	31 (91.2%)
QUID dimers (monomers)	11	3 (27.3%)	3 (27.3%)	3 (27.3%)	11 (100.0%)	3 (27.3%)
Case: capped alanine peptides	3	0 (0.0%)	0 (0.0%)	0 (0.0%)	0 (0.0%)	0 (0.0%)
Case: proline/β-cyclodextrin	2	0 (0.0%)	0 (0.0%)	2 (100.0%)	1 (50.0%)	2 (100.0%)
Case: alanine dipeptide microsolvation	3	3 (100.0%)	3 (100.0%)	3 (100.0%)	3 (100.0%)	3 (100.0%)
Case: 2-methoxycyclohexanone	3	2 (66.7%)	2 (66.7%)	2 (66.7%)	1 (33.3%)	2 (66.7%)
Case: methanol IR demo	1	1 (100.0%)	1 (100.0%)	1 (100.0%)	1 (100.0%)	1 (100.0%)
**All (unique)**	4568	162 (3.5%)	174 (3.8%)	860 (18.8%)	610 (13.4%)	1343 (29.4%)

Tables S42 and S43 summarize the overlap between the 4568 unique benchmark molecules and the training data of the different models. The fraction of benchmark molecules found in the training data ranges from 3.5–3.8% for the SPICE-only MACE-OFF23/24 models (162 and 174 molecules) to 13.4% for SO3LR v1 (610), 18.8% for MACE-OMol (860), and 29.4% for SO3LR-2 (1343). The larger overlap observed for the OMol25- and QCML-based models is primarily a consequence of the size and breadth of their underlying corpora: OMol25 incorporates ANI-2x, Transition1x, GEOM, OrbNet-Denali, and SPICE, while QCML contains approximately 33.5 million small-molecule structures. When OMol25 is traced through its constituent public sources, the number of benchmark molecules with a corresponding entry increases from 860 in the direct scan of the local neutral subset, which provides a lower bound, to 2107 in the union of the constituent datasets, which provides an upper bound. More generally, all counts relying on 3D bond perception or the QCML formula pre-filter should be regarded as lower bounds. The observed overlap is dominated by the small, common organic molecules in the NIST35 infrared set, including simple alkanes, alcohols, amines, and other drug-like fragments that recur across large small-molecule datasets such as SPICE, QM7-X, QCML, and OMol25. This overlap is therefore expected for a gas-phase IR benchmark composed largely of common laboratory compounds. In contrast, substantially less overlap is observed for datasets targeting less common chemistry or larger molecular systems: TorsionNet500 (495 molecules) yields only 19 molecule-level matches with any individual training set, while the macrocyclic and larger case-study systems, including the MPCONF196

macrocycles, β-cyclodextrin, and the longer capped peptides, largely fall outside the size range of the size-limited small-molecule training sets (SPICE, DES, AQM, QM7-X, GEMS) and are absent from them. The broadest corpora do contain a few of these larger species—β-cyclodextrin occurs in OMol25, and isolated MPCONF196 macrocycles in OMol25/QMugs—but they remain out of range of the size-restricted training sets. One notable exception is the capped peptide Ace-Ala$_{15}$-LysH$^+$ ($C_{53}H_{92}N_{17}O_{18}$), which is present in the GEMS dataset used to train SO3LR; this overlap is genuine and expected because GEMS contains minima-hopping ensembles generated specifically for this peptide. The three shorter peptides, Ace-Ala$_5$-NMe, Ace-Ala$_5$-LysH$^+$, and Ace-Ala$_{10}$-LysH$^+$, are distinct molecules and are not present in GEMS.

S18.4 Interpretation

Molecule-level familiarity does not, by itself, imply an artificially favorable benchmark, for several reasons. First, the reported metrics are *relative* quantities evaluated against independent high-level references: models are assessed on the *shape* of torsional profiles or on the *ordering* of conformers relative to PBE0+MBD, ωB97M-V, or CCSD(T), rather than on absolute molecular energies. Thus, having encountered a molecule during training does not provide the reference quantity being evaluated. Second, the benchmark conformers and reference geometries are generated independently in this work and are not training snapshots, such that even molecules whose connectivity is represented in a training set are evaluated on previously unseen geometries. Third, where the overlap is largest it is also least informative. It is dominated by small, ubiquitous species—the NIST35 infrared compounds, and the common solvents and small solutes that make up the microsolvation and host–guest case studies (water, diethyl ether, CH_3CN, proline)—whose presence in any broad training corpus is expected and which say nothing about the relative solvation shifts or conformational preferences those case studies actually measure on their freshly generated cluster geometries. Overlap is genuinely small precisely where the chemistry is less common and transferability is most stressed, most notably TorsionNet500 (at most 19 of 495 molecules in any single training set). For the production model (SO3LR v1), the one substantive large-system coincidence is Ace-Ala$_{15}$-LysH$^+$ in its GEMS training data; here, the assessment is based exclusively on relative backbone conformational preferences against the other potentials, and does not involve comparison with a training energy or geometry. These observations indicate that the identified train–test overlap is unlikely to be a major source of the reported benchmark performance and does not alter the qualitative conclusions of Section S15.

References

[1] W. Kabsch. A solution for the best rotation to relate two sets of vectors. *Acta Crystallogr., Sect. A*, 32(5):922–923, 1976.

[2] James Bradbury, Roy Frostig, Peter Hawkins, Matthew James Johnson, Chris Leary, Dougal Maclaurin, George Necula, Adam Paszke, Jake VanderPlas, Skye Wanderman-Milne, and Qiao Zhang. JAX: Composable transformations of Python+NumPy programs. https://github.com/jax-ml/jax, 2018.

[3] Mark E. Tuckerman, José Alejandre, Roberto López-Rendón, Andrea L. Jochim, and Glenn J. Martyna. A Liouville-operator derived measure-preserving integrator for molecular dynamics simulations in the isothermal–isobaric ensemble. *J. Phys. A: Math. Gen.*, 39(19):5629–5651, 2006.

[4] Dong C. Liu and Jorge Nocedal. On the limited memory BFGS method for large scale optimization. *Math. Program.*, 45(1):503–528, 1989.

[5] Erik Bitzek, Pekka Koskinen, Franz Gähler, Michael Moseler, and Peter Gumbsch. Structural relaxation made simple. *Phys. Rev. Lett.*, 97(17):170201, 2006.

[6] Roland Lindh, Anders Bernhardsson, Gunnar Karlström, and Per-Åke Malmqvist. On the use of a Hessian model function in molecular geometry optimizations. *Chem. Phys. Lett.*, 241(4):423–428, 1995.

[7] Xuan Liu, Sarathkrishna Swaminathan, Dmitry Zubarev, Brandi Ransom, Nathaniel Park, Kristin Schmidt, and Huimin Zhao. AccFG: Accurate functional group extraction and molecular structure comparison. *J. Chem. Inf. Model.*, 65(16):8593–8602, 2025.

[8] Adil Kabylda, J. Thorben Frank, Sergio Suárez-Dou, Almaz Khabibrakhmanov, Leonardo Medrano Sandonas, Oliver T. Unke, Stefan Chmiela, Klaus-Robert Müller, and Alexandre Tkatchenko. Molecular simulations with a pretrained neural network and universal pairwise force fields. *J. Am. Chem. Soc.*, 147(37):33723–33734, 2025.

[9] J. Thorben Frank, Oliver T. Unke, Klaus-Robert Müller, and Stefan Chmiela. A Euclidean transformer for fast and stable machine learned force fields. *Nat. Commun.*, 15(1):6539, 2024.

[10] Ilyes Batatia, Dávid Péter Kovács, Gregor N. C. Simm, Christoph Ortner, and Gábor Csányi. MACE: Higher order equivariant message passing neural networks for fast and accurate force fields. In *Adv. Neural Inf. Process. Syst.*, volume 35, pages 11423–11436, 2022.

[11] Dávid Péter Kovács, J. Harry Moore, Nicholas J. Browning, Ilyes Batatia, Joshua T. Horton, Yixuan Pu, Venkat Kapil, William C. Witt, Ioan-Bogdan Magdău, Daniel J. Cole, and Gábor Csányi. MACE-OFF: Short-range transferable machine learning force fields for organic molecules. *J. Am. Chem. Soc.*, 147(21):17598–17611, 2025.

[12] Marvin Friede, Christian Hölzer, Sebastian Ehlert, and Stefan Grimme. dxtb—an efficient and fully differentiable framework for extended tight-binding. *J. Chem. Phys.*, 161(6):062501, 2024.

[13] David Gruzman, Amir Karton, and Jan M. L. Martin. Performance of ab initio and density functional methods for conformational equilibria of C_nH_{2n+2} alkane isomers ($n = 4$–8). *J. Phys. Chem. A*, 113(43):11974–11983, 2009.

[14] Young Kee Kang and Hae Sook Park. Puckering transitions in cyclohexane: Revisited. *Chem. Phys. Lett.*, 702:82–89, 2018.

[15] Yue Liang, DingDing Lv, Guanjun Wang, Xiaoqing Zeng, Weixing Li, and Mingfei Zhou. Conformational landscapes of 2,3-, 2,4-, 2,5-, and 2,6-difluorobenzaldehyde unveiled by rotational spectroscopy. *J. Phys. Chem. A*, 128(39):8305–8311, 2024.

[16] Lincan Fang, Esko Makkonen, Milica Todorović, Patrick Rinke, and Xi Chen. Efficient amino acid conformer search with Bayesian optimization. *J. Chem. Theory Comput.*, 17(3):1955–1966, 2021.

[17] Rubicelia Vargas, Jorge Garza, Benjamin P. Hay, and David A. Dixon. Conformational study of the alanine dipeptide at the MP2 and DFT levels. *J. Phys. Chem. A*, 106(13):3213–3218, 2002.

[18] Jan Řezáč, Daniel Bím, Ondrej Gutten, and Lubomír Rulíšek. Toward accurate conformational energies of smaller peptides and medium-sized macrocycles: MPCONF196 benchmark energy data set. *J. Chem. Theory Comput.*, 14(3):1254–1266, 2018.

[19] Justyna Wielińska, Justyna Bednarko, Henryk Myszka, Beata Liberek, and Andrzej Nowacki. Cyclophosphamide and isophosphamide – DFT conformational studies in the gas phase and solution. *J. Mol. Graphics Modell.*, 90:243–257, 2019.

[20] Brajesh K. Rai, Vishnu Sresht, Qingyi Yang, Ray Unwalla, Meihua Tu, Alan M. Mathiowetz, and Gregory A. Bakken. TorsionNet: A deep neural network to rapidly predict small-molecule torsional energy profiles with the accuracy of quantum mechanics. *J. Chem. Inf. Model.*, 62(4):785–800, 2022.

[21] National Institute of Standards and Technology. NIST computational chemistry comparison and benchmark database, NIST standard reference database number 101, release 22. https://cccbdb.nist.gov/, 2022.

[22] Mirela Puleva, Leonardo Medrano Sandonas, Balázs D. Lőrincz, Jorge Charry, David M. Rogers, Péter R. Nagy, and Alexandre Tkatchenko. Extending quantum-mechanical benchmark accuracy to biological ligand-pocket interactions. *Nat. Commun.*, 16(1):8583, 2025.

[23] Jan Řezáč. Non-covalent interactions atlas benchmark data sets: Hydrogen bonding. *J. Chem. Theory Comput.*, 16(4):2355–2368, 2020.

[24] Jan Řezáč. Non-covalent interactions atlas benchmark data sets 2: Hydrogen bonding in an extended chemical space. *J. Chem. Theory Comput.*, 16(10):6305–6316, 2020.

[25] Sergio Suárez-Dou, Miguel Gallegos, Kyunghoon Han, Florian N. Brünig, Joshua T. Berryman, and Alexandre Tkatchenko. Quantum-accurate vibrational and conformational dynamics from small molecules to proteins with machine-learned force fields. arXiv preprint arXiv:2601.09845, 2026.

[26] Stephen E. Stein. NIST/EPA gas-phase infrared database (JCAMP format), NIST standard reference database 35. National Institute of Standards and Technology, Gaithersburg, MD, 2008.

[27] Philipp Pracht, David F. Grant, and Stefan Grimme. Comprehensive assessment of GFN tight-binding and composite density functional theory methods for calculating gas-phase infrared spectra. *J. Chem. Theory Comput.*, 16(11):7044–7060, 2020.

[28] Zhi-Xiang Wang and Yong Duan. Solvation effects on alanine dipeptide: A MP2/cc-pVTZ//MP2/6-31G** study of (Φ, Ψ) energy maps and conformers in the gas phase, ether, and water. *J. Comput. Chem.*, 25(14):1699–1716, 2004.

[29] M. P. Freitas, C. F. Tormena, and R. Rittner. Infrared spectroscopy and theoretical calculations as tools for the conformational analysis of 2-methoxycyclohexanone. *Spectrochim. Acta, Part A*, 59(6):1177–1182, 2003.

[30] Peter Pulay and Géza Fogarasi. Geometry optimization in redundant internal coordinates. *J. Chem. Phys.*, 96(4):2856–2860, 1992.

[31] Jon Baker. Techniques for geometry optimization: A comparison of cartesian and natural internal coordinates. *J. Comput. Chem.*, 14(9):1085–1100, 1993.

[32] Chunyang Peng, Philippe Y. Ayala, H. Bernhard Schlegel, and Michael J. Frisch. Using redundant internal coordinates to optimize equilibrium geometries and transition states. *J. Comput. Chem.*, 17(1):49–56, 1996.

[33] Vebjørn Bakken and Trygve Helgaker. The efficient optimization of molecular geometries using redundant internal coordinates. *J. Chem. Phys.*, 117(20):9160–9174, 2002.

[34] Lee-Ping Wang and Chenchen Song. Geometry optimization made simple with translation and rotation coordinates. *J. Chem. Phys.*, 144(21):214108, 2016.

[35] H. Bernhard Schlegel. Estimating the Hessian for gradient-type geometry optimizations. *Theor. Chim. Acta*, 66(5):333–340, 1984.

[36] Thomas H. Fischer and Jan Almlöf. General methods for geometry and wave function optimization. *J. Phys. Chem.*, 96(24):9768–9774, 1992.

[37] Daniel S. Levine, Muhammed Shuaibi, Evan Walter Clark Spotte-Smith, Michael G. Taylor, Muhammad R. Hasyim, Kyle Michel, Ilyes Batatia, Gábor Csányi, Misko Dzamba, Peter Eastman, Nathan C. Frey, Xiang Fu, Vahe Gharakhanyan, Aditi S. Krishnapriyan, Joshua A. Rackers, Sanjeev Raja, Ammar Rizvi, Andrew S. Rosen, Zachary Ulissi, Santiago Vargas, C. Lawrence Zitnick, Samuel M. Blau, and Brandon M. Wood. The Open Molecules 2025 (OMol25) dataset, evaluations, and models. arXiv preprint arXiv:2505.08762, 2026.
